# Investigating associations between JAK inhibition and venous thromboembolism by systematic mining of large-scale datasets

**DOI:** 10.1007/s10787-025-01677-2

**Published:** 2025-02-24

**Authors:** Stine Rabech Haysen, Ane Langkilde-Lauesen Nielsen, Per Qvist, Tue Wenzel Kragstrup

**Affiliations:** 1 Department of Biomedicine, Aarhus University, Aarhus, Denmark; 2 Centre for Genomics and Personalized Medicine, CGPM, Aarhus University, Aarhus, Denmark; 3 Department of Molecular Medicine, Aarhus University Hospital, Aarhus, Denmark; 4 Rheumatology Sector, Medical Diagnostic Center, Silkeborg Regional Hospital, Silkeborg, Denmark

**Keywords:** Janus kinase (JAK) inhibitor, Disease-modifying antirheumatic drug (DMARD), Side effects, Special warnings, Venous Thromboembolism (VTE), Cyclin-dependent kinases (CDKs)

## Abstract

Janus kinase inhibitors (JAKi) have been associated with an increased risk of venous thromboembolism (VTE) limiting the use of JAKi-based therapy. To improve risk stratification and drug development, it is crucial to understand the implication of dysregulated JAK-Signal Transducers and Activators of Transcription (STAT) signaling in the pathogenesis of VTE. The objective of this study is to clarify the putative genomic vulnerability to dysregulated JAK–STAT signaling in VTE through systematic mining of large-scale datasets generated from studies comparing VTE patients with healthy controls. Particularly, we assess the representation of entities of the JAK–STAT signaling pathway including STAT target genes among sets of miRNA, mRNA, and proteins differentially abundant in VTE patients, and we explore the putative cumulative genetic association of JAK–STAT signaling gene sets to VTE. Genes related to the JAK–STAT pathway were found significantly altered in VTE patients compared to healthy controls, indicating that genes under transcriptional control of STAT may be dysregulated in VTE. In support of this notion, we find a significant overrepresentation of predicted STAT target genes among genes downregulated in VTE patients, and promoter sequences of differentially regulated genes were significantly enriched with STAT transcription factor binding site motifs. Further linking STAT signaling to the molecular signature of VTE, genes targeted by miRNAs differentially regulated in patients are significantly enriched with STAT target genes and genes acting in the JAK–STAT signaling pathway. Together, our findings indicate that disruptions in the JAK–STAT pathway contribute to the molecular profile of VTE. This offers hope for identifying ways to interact with the JAK–STAT pathway that do not carry the risk of VTE.

**Supplementary Information:**

The online version contains supplementary material available at 10.1007/s10787-025-01677-2.

## Introduction

Immune-mediated inflammatory diseases (IMIDs) are a heterogeneous group of conditions such as rheumatoid arthritis (RA), psoriatic arthritis, and inflammatory bowel disease characterized by persistent systemic immune activation and tissue inflammation (Xin, Xu et al. 2020). Anti-inflammatory therapies targeting cytokines, receptors, or interfering with downstream signal transduction pathways have revolutionized the treatment of IMIDs (Schwartz, Bonelli et al. 2016). Janus kinase inhibitors (JAKi) are one of the therapeutic options. These drugs act by inhibiting the Janus Kinase-Signal Transducer and Activator of Transcription (JAK–STAT) pathways and have the potential to mediate immune modulatory benefits across a range of pathologies resulting in a wide anti-inflammatory effect (Nash, Kerschbaumer et al. 2021, Kragstrup, Glintborg et al. 2022, Yang, Kragstrup et al. 2023). JAKi are effective and equal to or even more efficacious than treatment with biological drugs in some IMIDs (Kotyla 2018). However, JAKi therapy has been associated with an increased risk of venous thromboembolism (VTE) including deep vein thrombosis (DVT) and pulmonary embolism (PE) (Ibrahim and Scott 2020, Mease, Charles-Schoeman et al. 2020, Maqsood, Weber et al. 2022, Ytterberg, Bhatt et al. 2022). So far, the underlying mechanisms have remained unclear, and it is not known whether the association between JAKi treatment and VTE is due to a genomic vulnerability to dysregulated JAK–STAT signaling in VTE.

The JAK–STAT pathway is a classic pathway for several cytokines and growth factors. The binding of cytokines or growth factors initiates JAK-mediated cross-phosphorylation and recruitment of one or more STATs, causing a phosphorylation cascade (Jamilloux, El Jammal et al. 2019). This mediates a dimerization and translocation of STAT to the nucleus where it recognizes specific DNA sequences and in turn regulates the transcription of downstream target genes (Banerjee, Biehl et al. 2017). The intracellular tyrosine kinases called JAKs includes JAK1, JAK2, JAK3, and TYK2, and the STAT family includes STAT1, STAT2, STAT3, STAT4, STAT5a, STAT5b, and STAT6. Each cytokine uses a specific combination of JAK and STAT molecules to transduce a unique signal. Under normal conditions, the JAK–STAT signaling is essential for cell growth regulation and the development and function in both innate and adaptive immunity. The signaling is tightly regulated at multiple levels by activators and inhibitors in a complex network that intersect with many other intracellular pathways (O'Shea and Plenge 2012, Yan, Gibson et al. 2018). In response to growth factor stimulation, several JAK–STAT target genes including cyclins will be regulated. Cyclin D and cyclin-dependent kinase 4/6 (CDK4/6) regulate cell progression in early gap 1 phase (G1) in the cell cycle. CDK4/6 is responsible for the hyperphosphorylation of retinoblastoma (RB) proteins resulting in dissociation of the transcription factor E2F (Narasimha, Kaulich et al. 2014). The activation of E2F target genes is needed to allow the cell to proceed the cell cycle and division (Lukasik, Zaluski et al. 2021).

Here, we want to examine the putative converging of molecular VTE risk factors on JAK–STAT signaling, including STAT target genes, to understand the connection between JAKi-based therapy and the increased rates of VTE. This might improve risk stratification and development of future drugs with less side effects.

## Methods

**Fig. 1:**
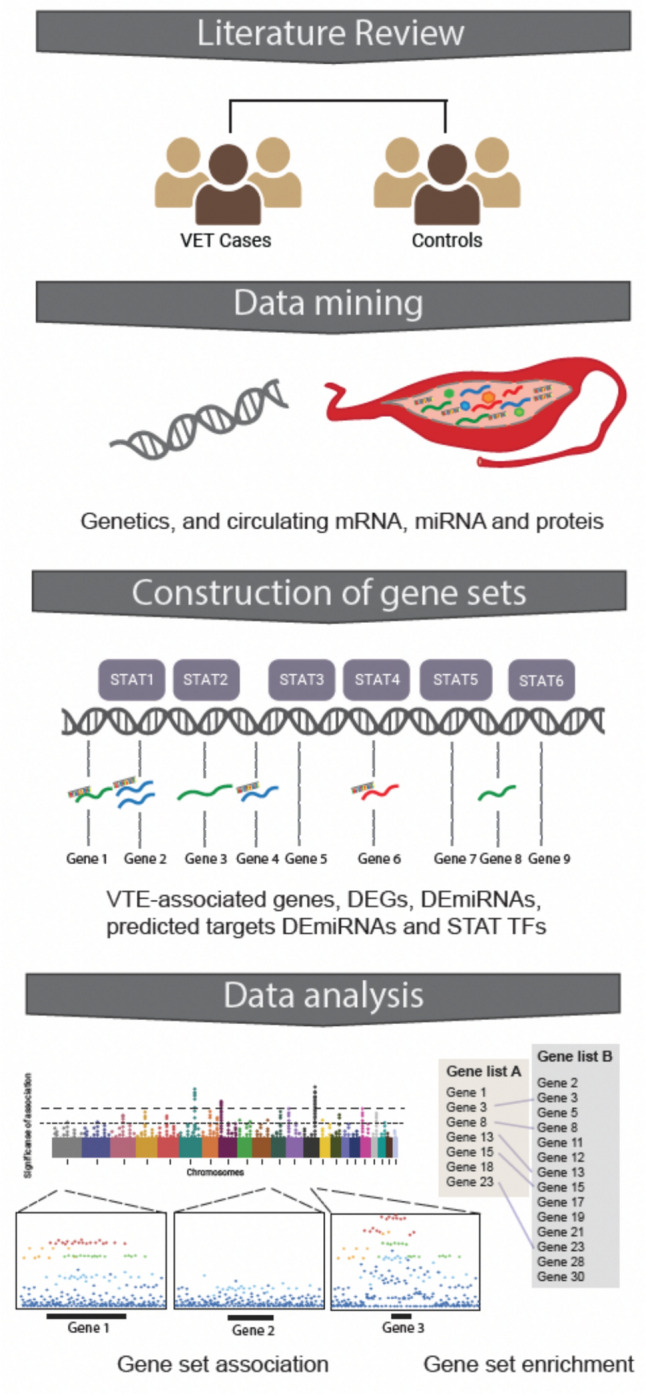
Methodological overview. Created with BioRendor.com

This study was based on integrative bioinformatics analyses of publically available data identified through literature and data mining. For an overview of the study setup, see Fig. 1.

### Large-scale datasets

A literature search was performed in the NCBI’s PubMed database (https://pubmed.ncbi.nlm.nih.gov, with last access December 2023) for studies specifically aimed at selection and validation of molecular entities linked to VTE. The literature was screened according to the PRISMA (Preferred Reporting Items of Systematic Reviews and Meta-Analyses) guidelines (Liberati, Altman et al. 2009). Datasets were found by combining the MeSH (Medical Subject Heading) terms: *((GWAS) OR (gene expression) OR (microRNA) OR (transcriptomics) OR (proteomics) AND (venous thromboembolism))*. We excluded papers not fulfilling the following criteria based on filters in PubMed: 1) species: human; 2) study language: English; 3) age: adult ($\ge$ 18 years); and 4) study comparing VTE cases and healthy controls. We then read title, abstract and full text and excluded papers not investigating: 1) Single-Nucleotide Polymorphisms (SNPs); 2) Differentially Expressed Genes (DEGs); 3) differentially expressed Micro-Ribonucleic Acid (miRNAs) (DEmiRNAs); or 4) Differentially Abundant Proteins (DAPs). Further exclusion criteria were participants with the following VTE risk factors: active cancer, antiphospholipid syndrome, COVID-19, recent surgery or trauma, use of contraceptive pills or hormonal therapy, immobilization, and pregnancy. Finally, all studies were filtered after the relevance of the topic and data validation leaving four original studies, which we deemed eligible and were selected as the final set of papers (Figure S1).

### Gene sets

*JAK–STAT gene set:* The JAK–STAT gene set includes genes that encode all entities of the pathway including cytokines, growth factors, cell-surface receptors, and intracellular signaling molecules. Genes annotated to the JAK–STAT pathway were defined by Kyoto Encyclopedia of Genes and Genomes (KEGG: https://www.genome.jp/entry/pathway+hsa04630) which is a bioinformatics resource for linking large-scale molecular datasets to biological pathways (Table S1).

*JAK–STAT target gene set:* Genes containing STAT deoxyribonucleic acid (DNA) consensus motifs within promotors [transcription start site (TSS) – 2000 bp] and thereby potentially governed by STAT transcription factors (TFs) were identified using the tool PWMScan (Ambrosini et al. 2018). Available TF-binding sites (TFBSs) linked to STAT1, STAT1:STAT2 heterodimer, and STAT3 (STAT target genes) were defined by the JASPER database (Rauluseviciute, Riudavets-Puig et al. 2023) (Table S2).

*VTE-associated genes:* Genes located in genome-wide associated loci identified in a VTE GWAS meta-analysis, as defined by independent significant SNPs in the GWAS summary statistics (Lindstrom, Wang et al. 2019), were extracted, and mapped using the tool Functional Mapping and Annotation (FUMA) of GWAS (Watanabe, Taskesen et al. 2017). Finally, regional association plots of loci for selected genes were generated with LocusZoom.js (Boughton, Welch et al. 2021).

*Target gene sets of miRNAs dysregulated in VTE:* Predicted target genes of miRNA found differentially expressed in VTE patients were identified using three different databases: MiRSystem (http://mirsystem.cgm.ntu.edu.tw/), MiRTarBase (https://mirtarbase.cuhk.edu.cn/~miRTarBase/miRTarBase_2022/php/index.php), and miRDB version 4.0 (https://mirdb.org), available for free academic use.

The Biomart community portal hosted by Ensembl (Cunningham, Allen et al. 2022) was used to convert all gene IDs to gene symbols, facilitating a common nomenclature space across studies.

### Gene-set enrichment analysis

For the analysis of overlap between JAK–STAT genes, STAT family TFBSs containing genes and genes in VTE-associated loci, DEGs, miRNA target genes, and DAPs, we used a web-based Venn diagram tool hosted by VIB/Ghent University (Bioinformatics & Evolutionary Genomics: https://bioinformatics.psb.ugent.be/webtools/Venn/). Significance of overlaps was determined using Chi-squared test statistics with a significance cut-off set at *p* value < 0.05.

Gene-set enrichment analysis were performed on DEG list using a designated tool for predicting and analyzing transcription factor-binding sites (CiiiDER) (Gearing, Cumming et al. 2019). CiiiDER extracts promoter sequence data (TSS – 2000 bp) for individual genes in query and background gene sets and compares the distribution of TFBSs extracted from the JASPAR database (Sandelin, Alkema et al. 2004) among query gene promoter sequences to the distribution of non-query (background) genes. Thereby it is possible to identify TFBSs that are statistically overrepresented among query genes. For background gene set, we used 5000 randomly selected non-differentially expressed genes represented on the microarrays used for the transcriptomic studies.

### Gene-set association analysis

Genetic variants in the VTE GWAS summary statistics (Lindstrom, Wang et al. 2019) locating to JAK–STAT and STAT target genes were included in a gene-set association analysis in MAGMA (de Leeuw, Mooij et al. 2015) using default settings, while excluding imputed SNPs with info score < 0.8. Analysis was based on summary statistics from the above-mentioned VTE GWAS meta-analysis (Lindstrom, Wang et al. 2019). MAGMA is a bioinformatic tool that calculates the accumulative trait association of a query gene set.

## Results

### Putative molecular risk factors in VTE

Four studies linking molecular entities to VTE fulfilled the inclusion criteria and were included as a source for large-scale data for the study (Figure S1 and Table 1**)**.

**Table 1:** Combined list of the included VTE data sets on different genomic levels

VTE data sets	GWAS Lindstrom, S., et al. (2019)	DEGs Lu, S., et al. (2021)	miRNA target genes Wang, X., et al. (2019)	DAPs Jensen, S.B., et al. (2018)
Setting	Multi-ancestry GWAS meta-analysis of VTE from the INVENT consortium based on prospective cohort and case–control data from 18 studies	Expression profiling by microarray based on a case–control study with GEO-numbers: GSE48000 and GSE19151 from the Thrombosis and Hemostasis Centers Research, University of North Carolina	miRNA data based on a prospective population-based study conducted from the Malmö Thrombophilia Study (MATS), South Sweden	Patients recruited from the fourth survey of the Tromsø study, University Hospital of North Norway
Participants	Adult men and women of European or African American ancestry. 30,234 VTE cases and 17,122healthy controls Females: 64.1% Mean age (years): 58.7	177 VTE cases and 88 healthy controls Females: 53% Mean age (years): 50.4	39 VTE cases and 39 healthy controls Females: 41% Mean age (years): 65.5	Adults older than 24 years. 100 VTE cases and 100 healthy controls Females: 57.7% Mean age (years): 65
Study material	Whole blood and liver tissue	RNA isolated from whole blood	Circulating miRNA from plasma samples	Blood plasma
Diagnosis	VTE event (DVT or PE)	GSE48000: 1) one or more provoked VTE, 2) single unprovoked VTE and 3) ≥ 2 unprovoked VTE GSE19151: single or recurrent VTE	Recurrent VTE	VTE (DVT or PE)
Methods	Analysis of SNPs	Microarrays	qPCR	Mass spectrometry

*Genetics:* We included summary statistics of a large-scale GWAS meta-analysis based on 18 studies from the International Network Against Venous Thrombosis (INVENT) (Lindstrom, Wang et al. 2019). The study included a total of 30,234 cases with VTE and 17,122 healthy controls. The participants were 64.1% females with a mean age of 58.7 years. The study identified 16 novel susceptibility loci for VTE (Lindstrom, Wang et al. 2019). Using summary statistics from the GWAS meta-analysis, we recreated the reported genetic association landscape of VTE (Figure S2) and extracted a list of 147 genes that are annotated to the identified 16 genome-wide significant (GWS) loci (Table S3).

*Transcriptomics:* We included DEGs from a meta-analysis of data from two independent transcriptomic studies of VTE by Lewis et al. (Lewis, Stashenko et al. 2011, Lewis, Suchindran et al. 2015, Lu, Lijuan et al. 2021). The study comprised a total of 177 cases with venous thromboembolism (VTE) and 88 healthy controls predominantly comprising females (53%) with a mean age of 50.4 years. The RNA was isolated from whole blood samples and DEGs were found by microarrays. One study included three categories of VTE cases: 1) those with one or more provoked VTE, 2) individuals with a single unprovoked VTE, and 3) those with two or more unprovoked VTE. The other encompassed cases with either single or recurrent VTE. The meta-analysis reported a list of 125 upregulated genes and 48 downregulated genes in VTE across the three categories (Table S4).

In addition, we included differentially expressed miRNA (DEmiRNAs) from a prospective population-based investigation conducted as part of the Malmö Thrombophilia Study (MATS) (Wang, Sundquist et al. 2019). The study comprised 39 individuals with recurrent venous thromboembolism (VTE) and 39 healthy controls. Among the participants, 41% were females with a mean age of 65.5 years. miRNA expression levels were measured in plasma samples by quantitative polymerase chain reaction (qPCR). 12 DEmiRNAs were reported in this study (Table S5). In addition to the DEmiRNAs themselves, we further included lists of genes predicted to be targeted by the identified miRNAs (Table S5).

*Proteomics:* We included DAPs from a project using the the Tromsø study (Jensen, Hindberg et al. 2018). The inclusion criteria comprised adults aged 24 years and older, with a total of 100 individuals with venous thromboembolism (VTE) cases and 100 healthy controls. Among the participants 57.7% were females and the mean age was 65 years. Plasma samples were analyzed by mass spectrometry and 46 DAPs were identified (Table S6).

### Genetic VTE association of genes implicated in JAK–STAT signaling

**Fig. 2:**
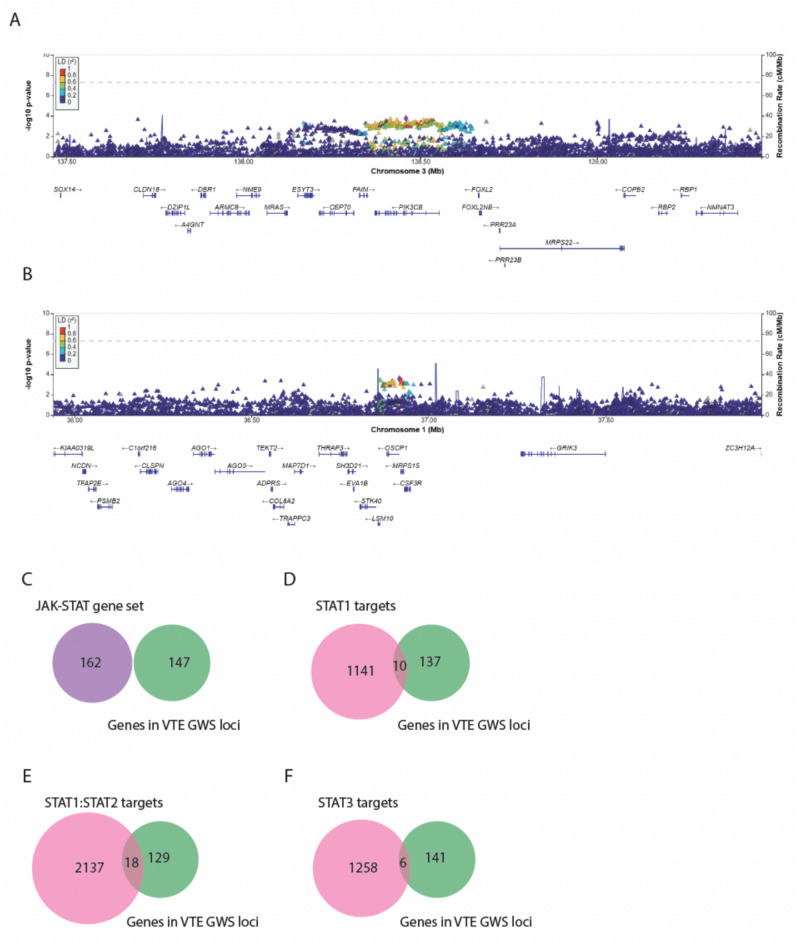
**A**–**B** Manhattan plot by chromosome of the VTE-associated loci. **C**–**F** Venn diagram of the overlap between GWAS VTE-associated genes and JAK–STAT genes and STAT target genes

We first independently assessed the representation of genes annotated to the JAK–STAT pathway as well as the list of STAT target genes among the 147 genes annotated to VTE-associated loci, but no significant overlap was found. Genetic loci displaying genome-wide significance, however, account for only the most significant, small fraction of the total heritability of VTE. Hence, to further explore the genetic VTE burden in JAK–STAT signaling, we employed a gene-set analysis approach based on the aggregated association of individual genetic markers within the JAK–STAT pathway and STAT target gene sets. While individual loci harboring JAK–STAT genes displayed regional nominal significant association to VTE (Fig. 2A–B**)**, the JAK–STAT gene set showed no cumulative association to VTE (Fig. 2C; *p* = 0.97). Applying the same gene-set association approach to the STAT target gene sets did not reveal significant associations either [STAT1 (Fig. 2D; *p* = 0.47), STAT1:STAT2 (Fig. 2E**;** *p* = 0.17), and STAT3 (Fig. 2F; *p* = 0.20)].

### JAK–STAT signaling and the VTE regulome

**Fig. 3:**
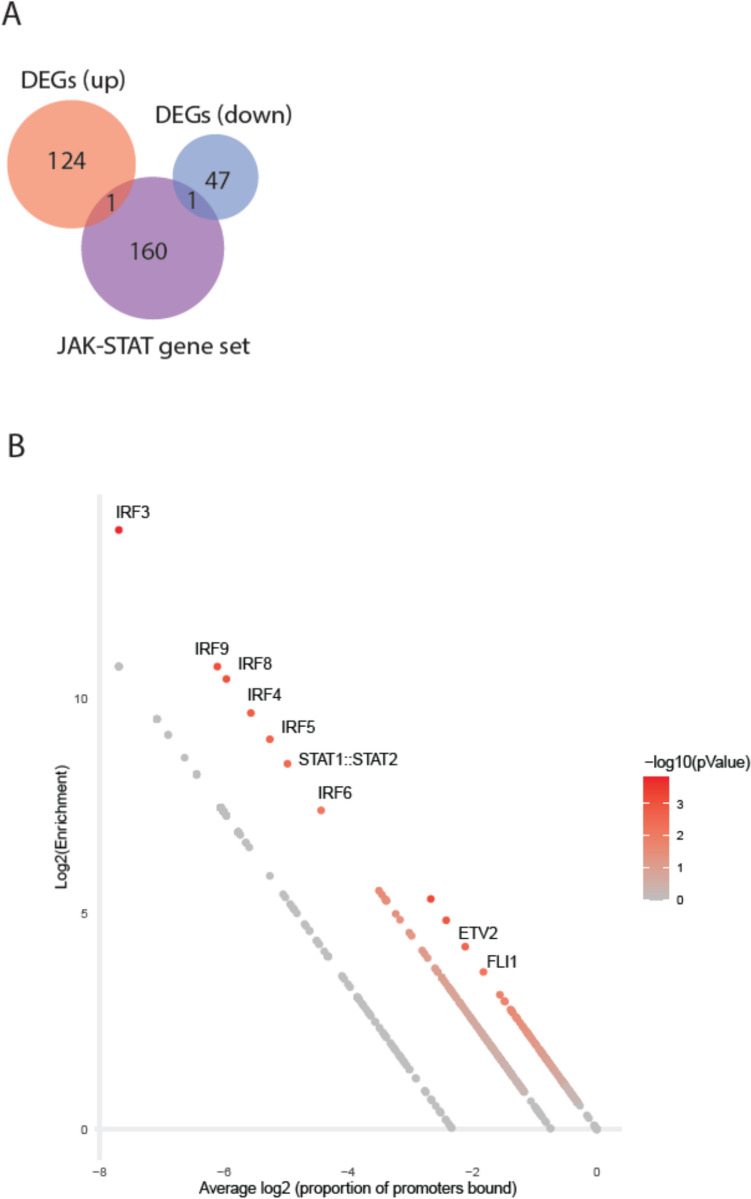
**A** Venn diagram of the overlap between VTE enriched DEGs with genes annotated to the JAK–STAT signaling pathway. **B** CiiiDER enrichment analysis. Graph shows: y axis: Log2 enrichment of individual TFBS in promoter sequence of downregulated DEG list compared to list of genes not on DEG list; x-axis: average proportion of genes containing each TFBS (close to 0 means that TFBS is present in the majority of gene promotors). Color code marks statistical significance of enrichment

To investigate a putative transcriptomic signature of JAK–STAT and STAT target genes in patients with VTE, we examined DEGs reported in a meta-analysis of large-scale mRNA studies in VTE (Lu, Lijuan et al. 2021) in which a total of 173 DEGs, including 125 upregulated genes and 48 downregulated genes, were reported. We did not find the DEGs to be enriched with genes annotated to the JAK–STAT signaling pathway, although individual genes from the JAK–STAT gene set were present among DEGs (Fig. 3A; p_up_ = 0.92/p_down_ = 0.91). However, utilizing the availability of experimentally verified binding site motifs in humans for more than 1500 transcription factors, we applied an enrichment analysis to identify TFs that are significantly overrepresented amon the promoter sequences of DEGs compared to the promoter sequences of non-DEGs form the same study. Suggesting that STAT family of TFs are among the main drivers of the transcriptional changes in VTE, we found a weak but statistically significant enrichment of the STAT1:STAT2 heterodimer TFBS motif among promoter sequences of upregulated DEGs (Fig. 3B, p = 0.003).

In a study of circulating miRNAs in patients with recurrent VTE, Wang, X., et al. (2019) identified 12 significantly dysregulated miRNAs: *miR-15b-5p*, *miR-106a-5p*, *miR-197-3p*, *miR-652-3p*, *miR-361-5p*, *miR-222-3p*, *miR-26b-5p*, *miR-532-5p*, *miR-27b-3p*, *miR-21-5p*, *miR-103a-3p*, and *miR-30c-5p*. None of these localize to VTE GWAS loci. However, it should be noted that many of the differentially expressed miRNAs are located on the X chromosome, for which no association data were available. For each DEmiRNA, a list of predicted target genes was extracted and their overlap with the JAK–STAT and STAT target gene lists were assessed. DEmiRNA target regulomes nominally significantly enriched with JAK–STAT genes are listed in Figure S3. Applying a conservative Bonferoni correction for multiple testing, only the regulomes of *miR-26b-5p* (*p* = 0.0001) and *miR-21-5p* (*p* = 0.0001) remained significantly enriched with predicted target genes of the STAT1:STAT2 heterodimer.

### JAK–STAT signaling and the VTE proteome

Finally, Jensen, S.B., et al. (Jensen, Hindberg et al. 2018) conducted a case–control proteomics study of 100 VTE cases and 100 healthy controls using untargeted mass spectrometry-based plasma proteomics. The authors reported 46 DAPs associated with VTE case–control status. Of the 46 proteins, only 35 had a corresponding unique gene ID. No significant overlaps with the JAK–STAT pathway and STAT target genes were observed for these 35 genes.

## Discussion

The occurrence of VTE when using JAKi is a rare but a serious adverse event. It is crucial to understand the possible implication of dysregulated JAK–STAT signaling in the pathogenesis of VTE. We performed a large-scale bioinformatic mining of genomic data from studies assessing patients with VTE compared to healthy controls. Whereas we did not find robust direct evidence of genetic vulnerability to altered JAK–STAT signaling in VTE, we did find data linking transcriptomic changes in VTE cases to this signaling pathway. Particularly, the transcriptomic signature of VTE and targets of miRNA differentially expressed in VTE suggest that genes under transcriptional control of particularly the STAT1:STAT2 heterodimer TF complex may be dysregulated in VTE.

The governance of gene expression involves two fundamental mechanisms including regulation by TFs (transcriptional regulation) and miRNA (post-transcriptional regulation). It is well known that these mechanisms exert facilitative or inhibitory effects on expression of their target genes, and their importance in a wide range of biological processes in pathological conditions has been demonstrated (Han, Cho et al. 2018, Morelli, Braekkan et al. 2020). Gene expression profiles are altered in patients with VTE compared to healthy controls and our results indicate that the STAT1:STAT2 heterodimer TF complex may play a significant role in facilitating these changes. Intriguingly, the STAT1:STAT2 TFBS was among the top 10 enriched TFBSs in the promoter sequences of DEGs, nearly exclusively surpassed by members of the interferon regulatory factor (IRF) group of TFs. Furthermore, the target regulomes of several differentially expressed miRNAs were significantly enriched with genes encoding the JAK–STAT pathway or STAT target genes.

**Fig. 4:**
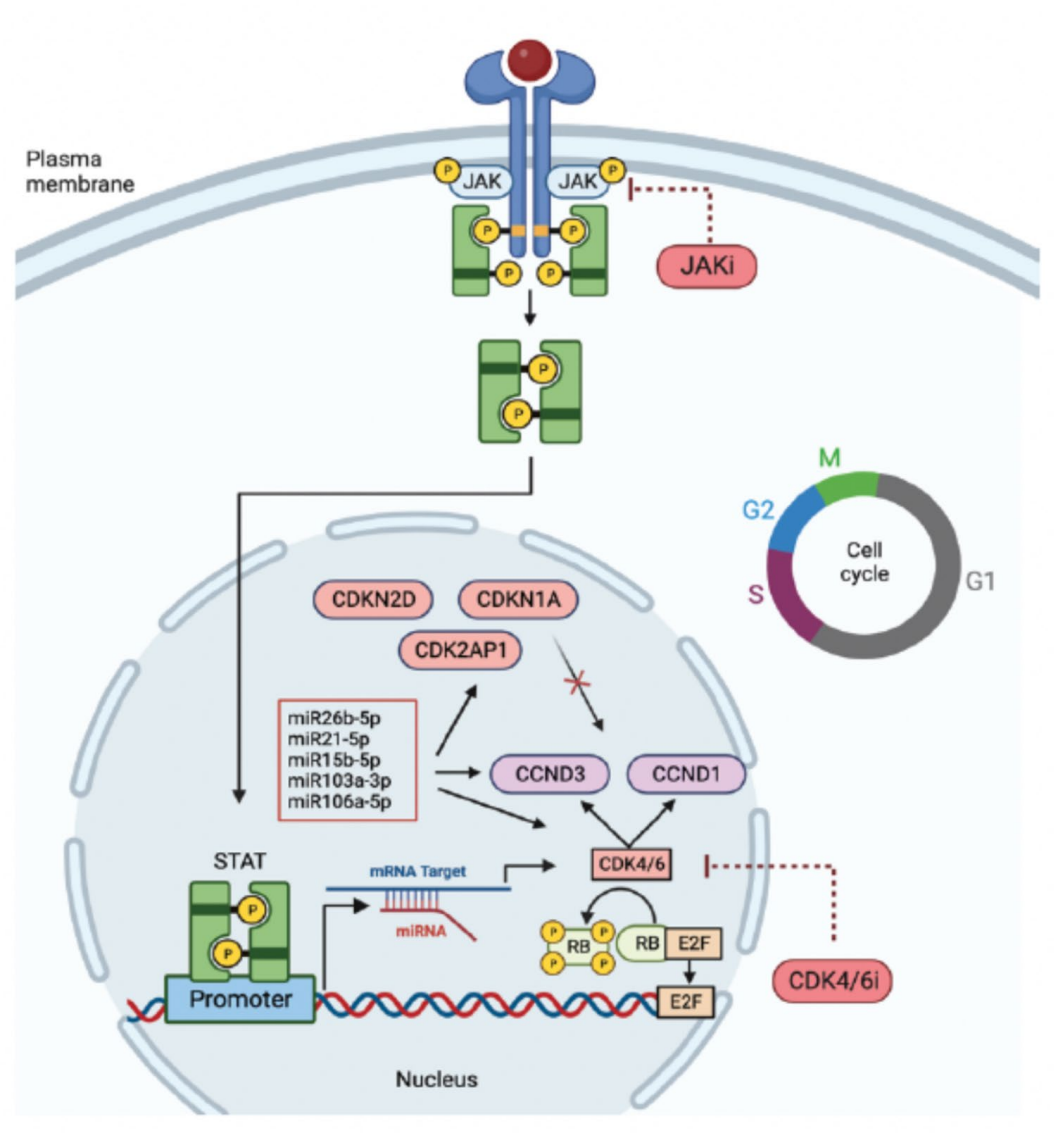
Illustration of the hypothetical connection, based on our results, between JAK–STAT signaling, miRNAs (miR26b-5p, miR21-5p, miR15b-5p, miR103a-3p, and miR106a-5p), cyclins (Cyclin D: CCND3, and CCND1), cyclin-dependent kinases (CDKs: CDKN1A, CDK2AP1, and CDKN2D), and the cell cycle suggesting that inhibition with either JAKi or CDK4/6i could alter the gene expression that may be responsible for the development of VTE. Created with BioRendor.com

Adding to the putative link between JAK–STAT signaling and VTE through cytokine signaling and cell cycle regulation, among the predicted targets of DEmiRNAs that overlap with JAK–STAT pathway-, and target genes are several genes encoding cyclins and cyclin-dependent kinases (CDKs), such as *CCND1*, *CCND3*, and *CDKN1A.* Furthermore, VTE-associated CDK encoding genes, CDKN2D and *CDK2AP1*, both locate to VTE GWS loci. The interactions of miRNA, cyclins, and CDKs facilitate the cell cycle progression. Blocking this pathway has led to important oncological therapeutic innovations such as CDK4/6i. However, previous studies have shown that VTE also emerged as a significant treatment-related adverse event to CDK4/6i (West, Kartika et al. 2022). Thrombotic events occur in up to 5% of clinical patients treated with CDK4/6i and have been reported in up to 10% in real-world analysis (West, Smith et al. 2021). Therefore, it can be speculated that there is a certain connection between JAK–STAT signaling, VTE, cyclins, and the cell cycle, as illustrated in Fig. 4. These findings would be an area for further investigation.

The relevance and magnitude of VTE in patients treated with JAKi are extremely high, since it affects the risk of hospitalization and patients’ quality of life, which contribute to high morbidity and mortality (Cohen, Agnelli et al. 2007). VTE is a complex disorder with a multicausal etiology including a genetic component (Zoller, Svensson et al. 2020). Since the description of Virchow’s triad explaining the pathophysiology of VTE involving: 1) stasis, 2) hypercoagulability, and 3) endothelial dysfunction, there have been rapid progress in the understanding of the pathogenesis and factors responsible for VTE (Kumar, Hanlin et al. 2010). Previous studies have shown that aging, long-term immobilization, trauma, cancer, virus infection, and genetic risk factors such as classical thrombophilia: factor V Leiden (rs6025) and prothrombin G20210A mutation (rs1799963), and deficiencies of antithrombin, protein C, and protein S contribute to VTE risk (Gohil, Peck et al. 2009; Crous-Bou et al. 2016). However, this cannot explain the association of VTE with JAKi-based therapy. It is well established that patients with IMIDs such as RA have an increased risk of VTE events, because inflammation promotes a thromboembolic state itself (Yates, Mootoo et al. 2021, Atzeni, Popa et al. 2022). Due to the relation between inflammation and VTE, it would be expected that the anti-inflammatory effects of JAKi would decrease the risk of VTE. It has to be considered that many outside factors, not examined in this study, are associated with the development of VTE and may influence the levels of gene expression.

Limitations of the present study were the validity of data available for analysis. The individual cohorts were clinically heterogeneous, differing by their sample sizes, proportion of patients with PE, and the time of sample collection since their most recent event. Thus, the investigation focused on different genomic levels from genetic variation associated with VTE to differentially expressed genes, miRNAs, and proteins, as well as their predicted genomic targets. Of note, expression data were based on microarray and PCR-based data and thus not true transcriptome-wide by nature, and the proteomics study was particularly limited by its small samples size and associated lack of power.

Here, we provide a coherent approach to assess the genomic basis for the reported association between JAKi treatment and VTE. JAK–STAT genes and STAT target genes were significantly altered in VTE patients compared with healthy controls, suggesting that genes under transcriptional control of STAT may be dysregulated in VTE. Furthermore, some of the overlapping genes include cyclins indicating a putative connection between JAK–STAT, VTE, cyclins, and the cell cycle, which provides a basis for further investigation.

## Supplementary Information

Below is the link to the electronic supplementary material.

Supplementary file1 (PDF 350 KB)

Supplementary file2 (PDF 1237 KB)

Supplementary file3 (PDF 1723 KB)

Supplementary file4 (DOCX 81 KB)

## Data Availability

We used publicly available data sets.

## Abbreviations

CDK4/6: Cyclin-dependent kinase 4/6
CVD: Cardiovascular disease
DAPs: Differentially Abundant Proteins
DEGs: Differentially Expressed Genes
DNA: Deoxyribonucleic acid
DVT: Deep vein thrombosis
FDA: Food and drug administration
FUMA: Functional Mapping and Annotation
G1: Gap 1 phase
GEO: Gene Expression Omnibus
GF: Growth factors
GO: Gene Ontology
GWAS: Genome-wide association studies
GWS: Genome-wide significant
IMIDs: Immune-mediated inflammatory diseases
INVENT: International Network Against Venous Thrombosis
JAKi: Janus kinase inhibitors
JAK–STAT: Janus kinase-signal transducer and activator of transcription
KEGG: Kyoto Encyclopedia of Genes and Genomes
MeSH: Medical Subject Heading
miRNA: Micro-Ribonucleic Acid
NG-CHM: Next-Generation Clustered Heat Map
PE: Pulmonary embolism
PRISMA: Preferred Reporting Items of Systematic Reviews and Meta-Analyses
RA: Rheumatoid arthritis
RB: Retinoblastoma
SNPs: Single-Nucleotide Polymorphisms
SURV: Surveillance trail
