# Supplementary figures and images for: Investigating associations between JAK inhibition and venous thromboembolism by systematic mining of large-scale datasets
Source: Inflammopharmacology. 2025 Feb 24;33(3):1425–34. doi: 10.1007/s10787-025-01677-2 (PMC11913929; doi:10.1007/s10787-025-01677-2)

# Supplementary Figure 1

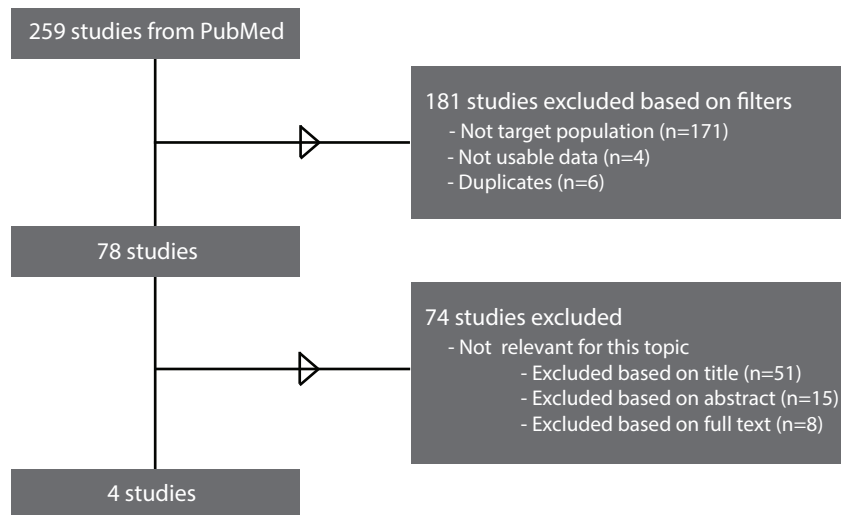

Supplement: Supplementary file 1 — Supplementary file1 (PDF 350 KB) [file 10787_2025_1677_MOESM1_ESM.pdf]

Supplementary Figure 1

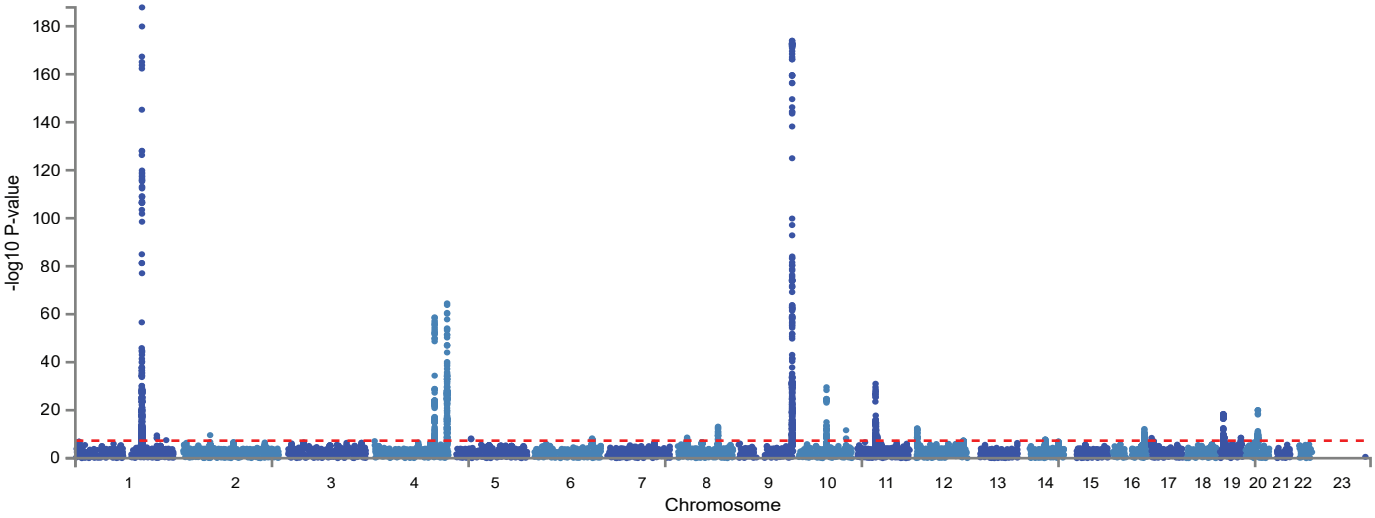

Supplement: Supplementary file 2 — Supplementary file2 (PDF 1237 KB) [file 10787_2025_1677_MOESM2_ESM.pdf]
