# Supplementary material for: Investigating associations between JAK inhibition and venous thromboembolism by systematic mining of large-scale datasets
Source: Inflammopharmacology. 2025 Feb 24;33(3):1425–34. doi: 10.1007/s10787-025-01677-2 (PMC11913929; doi:10.1007/s10787-025-01677-2)
Supplement: Supplementary file 3 — Supplementary file3 (PDF 1723 KB) [file 10787_2025_1677_MOESM3_ESM.pdf]

Supplementary Figure 3

	JAK-STAT genes	STAT1 genes	STAT1:2 genes	STAT3 genes
hsa-miR-15b-5p	JAK-STAT 142 · 20 · 1158 miRNA<br>**p = 0.0015**	STAT1 1090 · 61 · 1117 miRNA<br>p = 0.4838	STAT1:2 2014 · 141 · 1037 miRNA<br>**p = 0.0399**	STAT3 1191 · 73 · 1105 miRNA<br>p = 0.99
hsa-miR-106a-5p	JAK-STAT 146 · 16 · 1056 miRNA<br>**p = 0.0263**	STAT1 1082 · 69 · 1003 miRNA<br>p = 0.3249	STAT1:2 2016 · 139 · 933 miRNA<br>**p = 0.0027**	STAT3 1183 · 81 · 991 miRNA<br>p = 0.0776
hsa-miR-197-3p	JAK-STAT 161 · 1 · 409 miRNA<br>**p = 0.02648**	STAT1 1127 · 24 · 386 miRNA<br>p = 0.9738	STAT1:2 2113 · 42 · 368 miRNA<br>p = 0.9285	STAT3 1235 · 29 · 381 miRNA<br>p = 0.6663
hsa-miR-652-3p	JAK-STAT 162 · 0 · 170 miRNA<br>p = 0.4092	STAT1 1132 · 19 · 151 miRNA<br>**p = 0.0065**	STAT1:2 2142 · 13 · 157 miRNA<br>p = 0.2566	STAT3 1249 · 15 · 155 miRNA<br>p = 0.2835
hsa-miR-361-5p	JAK-STAT 157 · 5 · 294 miRNA<br>p = 0.2411	STAT1 1127 · 24 · 275 miRNA<br>p = 0.1533	STAT1:2 2112 · 43 · 256 miRNA<br>**p = 0.0382**	STAT3 1242 · 22 · 277 miRNA<br>p = 0.6089
hsa-miR-222-3p	JAK-STAT 159 · 3 · 430 miRNA<br>p = 0.8865	STAT1 1115 · 36 · 397 miRNA<br>**p = 0.0389**	STAT1:2 2099 · 56 · 377 miRNA<br>p = 0.1111	STAT3 1225 · 39 · 394 miRNA<br>**p = 0.0347**
hsa-miR-27b-3p	JAK-STAT 152 · 10 · 1033 miRNA<br>p = 0.8082	STAT1 1074 · 77 · 966 miRNA<br>**p = 0.0212**	STAT1:2 2032 · 123 · 920 miRNA<br>p = 0.0966	STAT3 1181 · 83 · 960 miRNA<br>**p = 0.0230**
hsa-miR-21-5p	JAK-STAT 144 · 18 · 1059 miRNA<br>**p = 0.0039**	STAT1 1076 · 75 · 1002 miRNA<br>p = 0.0796	STAT1:2 2007 · 148 · 929 miRNA<br>**p = 0.0001**	STAT3 1185 · 79 · 998 miRNA<br>p = 0.1429
hsa-miR-103a-3p	JAK-STAT 150 · 12 · 1265 miRNA<br>p = 0.7864	STAT1 1078 · 73 · 1204 miRNA<br>p = 0.9710	STAT1:2 1998 · 157 · 1120 miRNA<br>**p = 0.0094**	STAT3 1179 · 85 · 1192 miRNA<br>p = 0.5020
hsa-miR-30c-5p	JAK-STAT 146 · 16 · 1133 miRNA<br>p = 0.0510	STAT1 1076 · 75 · 1074 miRNA<br>p = 0.2322	STAT1:2 2110 · 145 · 1004 miRNA<br>**p = 0.0259**	STAT3 1173 · 91 · 1058 miRNA<br>**p = 0.0167**
hsa-miR-26b-5p	JAK-STAT 132 · 30 · 2957 miRNA<br>p = 0.1360	STAT1 960 · 191 · 2796 miRNA<br>**p = 0.0028**	STAT1:2 1800 · 355 · 2632 miRNA<br>**p = 0.0001**	STAT3 1070 · 194 · 2793 miRNA<br>p = 0.0587
hsa-miR-532-5p	JAK-STAT 162 · 0 · 184 miRNA<br>p = 0.3730	STAT1 1140 · 11 · 173 miRNA<br>p = 0.9899	STAT1:2 2134 · 21 · 163 miRNA<br>p = 0.8129	STAT3 1252 · 12 · 172 miRNA<br>p = 0.9919
