# Supplementary material for: Investigating associations between JAK inhibition and venous thromboembolism by systematic mining of large-scale datasets
Source: Inflammopharmacology. 2025 Feb 24;33(3):1425–34. doi: 10.1007/s10787-025-01677-2 (PMC11913929; doi:10.1007/s10787-025-01677-2)
Supplement: Supplementary file 4 — Supplementary file4 (DOCX 81 KB) [file 10787_2025_1677_MOESM4_ESM.docx]

**Table S1.**

Genes annotated to the JAK-STAT pathway was defined by Kyoto Encyclopedia of Genes and Genomes (KEGG: <https://www.genome.jp/entry/pathway+hsa04630>).

| Data number | Platform | Number of genes | Gene symbols |
| --- | --- | --- | --- |
| hsa04630 | KEGG | 162 | IL2, IL4, IL7, IL9, IL15, IL21, TSLP, IL2RA, IL2RB, IL2RG, IL4R, IL7R, IL9R, IL15RA, IL21R, CRLF2, IL3, IL5, CSF2, IL3RA, IL5RA, CSF2RA, CSF2RB, IL6, IL11, IL13, IL27, IL31, OSM, LIF, CNTF, CTF1, CLCF1, IL6R, IL6ST, IL11RA, IL13RA1, IL13RA2, IL27RA, IL31RA, OSMR, LIFR, CNTFR, IL10, IL19, IL22, IL20, IL24, IL26, IL10RA, IL10RB, IL20RA, IL20RB, IL22RA1, IL22RA2, IL12A, IL12B, IL23A, IL12RB1, IL12RB2, IL23R, EPO, GH1, GH2, CSH1, CSH2, PRL, THPO, CSF3, LEP, EPOR, GHR, PRLR, MPL, CSF3R, LEPR, IFNA1, IFNA2, IFNA4, IFNA5, IFNA6, IFNA7, IFNA8, IFNA10, IFNA13, IFNA14, IFNA16, IFNA17, IFNA21, IFNB1, IFNE, IFNK, IFNL1, IFNL2, IFNL3, IFNW1, IFNAR1, IFNAR2, IFNLR1, IFNG, IFNGR1, IFNGR2, EGF, PDGFA, PDGFB, EGFR, PDGFRA, PDGFRB, JAK1, JAK2, JAK3, TYK2, STAT1, STAT2, STAT3, STAT4, STAT5A, STAT5B, STAT6, IRF9, CISH, SOCS1, SOCS2, SOCS3, SOCS4, SOCS5, SOCS7, SOCS6, BCL2, MCL1, BCL2L1, PIM1, MYC, CCND1, CCND2, CCND3, CDKN1A, AOX1, GFAP, STAM2, STAM, PTPN2, PTPN6, CREBBP, EP300, PIAS1, PIAS2, PIAS3, PIAS4, FHL1, PTPN11, GRB2, SOS1, SOS2, HRAS, RAF1, PIK3CA, PIK3CD, PIK3CB, PIK3R1, PIK3R2, PIK3R3, AKT1, AKT2, AKT3, MTOR |

**Table S2.**

Available TF binding sites (TFBSs) linked to STAT1, STAT1:STAT2 heterodimer and STAT3 (STAT target genes) were defined by the JASPER database.

| Dataset | Number of genes | Gene symbols |
| --- | --- | --- |
| STAT1 | 1151 | SLC18A1 CLPB MMP2 HMCES SLMAP PLCE1 ARHGEF10L ZAP70 DNASE1 ZHX3 ZNF225 TSPAN11 NBAS GCSH YIF1B TACC2 NMB MACF1 IRAK1 GSTA1 IGF1 RAB1B FAM214A SQOR DDB1 DCAF5 GJD3 C1RL COL22A1 MITF KIR2DL2 TICAM1 C1QC LYG1 FCRL6 KCNA6 DDX11 TRIM74 ZNF446 IMP4 ZBED3 SP140L ANAPC4 SLC38A5 LRMDA KLHDC4 SLC4A8 DSP NDST1 GOLM1 SDCCAG8 KLC4 GPR143 CASP6 OSBPL8 ZNF181 TNS3 SPIN2B GK PIKFYVE CHID1 FLVCR1 SLC9A5 MTMR1 DUS2 DERA SYNGAP1 GPR162 SYN3 RAB8B ZFP3 SAMD3 ATG2B MAPKAPK2 ATXN10 MRPS14 CAMK1 KIR2DS3 KIAA1522 CDC25B ACVRL1 LIMS2 MECP2 PNPLA4 LIPN CNBD2 NOS1 ATP5MG A2M PALLD ABCG1 MOB3C ODF1 CPNE5 NUTM2G CPNE4 ZMYND15 PLN DGUOK FGGY RPP40 ZNF197 TRIM60 EML1 SMAD3 IFTAP SRRM4 SSTR2 SHISA3 LOC389199 ZBTB22 ZNF529 SLC2A13 SHROOM1 CLEC1B YTHDF3 PCDHA13 CD38 PGRMC2 HIF3A ZDHHC6 MFSD8 MYL12A PODN TEX19 TBC1D5 CNTN4 CFAP57 GSTA2 ZNF385B MAOA CASP10 RAP1GAP2 PLEKHB1 SRI ZFP37 FBXO31 TAF7L MRPL24 CEACAM20 IP6K1 HDAC7 GLRA2 POLE3 ZNF596 PRPF19 MRPL12 SOD3 KRTAP1-4 TEFM THAP12 UBR3 KRT26 PANX3 NLRP6 BPNT2 FNDC1 NABP2 ALKAL1 HNRNPR MEIS2 GCLM MCU IPO8 FAM167B ASIP MRAP2 P4HA3 TRMT112 SLC35D2 TLX2 CD36 PTP4A2 FAM76B MAEL ZNF284 TRIM29 ARF4 GKN1 RAB3A ATP11C SLC12A6 ELF3 RAP1GAP TOX3 NOP2 SLC9C1 GPCPD1 RGS4 TMEM92 CXCR2 SPTBN1 RICTOR IFITM5 LIPE PRR22 ZNF24 RPS27A COL21A1 ADCY5 RAI14 HMCN1 CYP1A1 PHF7 HOXB3 HHLA2 AKR1B15 ZNF547 SNX25 MRLN RGL1 DLGAP4 GSAP MBD5 C9orf163 PRKD3 TAP1 ACOT7 N4BP2 RASSF5 ATP6V1G3 ZBTB18 FAM24A DYNC1H1 FOLR1 GTPBP3 TMEM33 FAM156B TCF12 KRT71 SIPA1L2 ABCF2 GNG12 CFAP298 PLEKHG2 WDR20 COL7A1 ARMC8 CORO2B HSPA4 ZNF79 SLC7A14 HSPA8 RARB ZNF227 PRRT1B CNOT6 CXCL11 IDNK SELENOP PNOC DTX1 DRAM2 KRTAP19-6 UBE2L6 LDAH PRKAA1 CCR3 KIR3DL2 SMC4 MED12 C1orf141 AGTPBP1 ALDH1L2 TSHZ2 RPL22L1 DNHD1 PABPC3 CNGA1 HDAC9 CD58 RLN1 PARP14 TOM1L2 PTGFR SNX31 MLN DLX4 IL18BP OSGEP SMC6 CSRP3 CNR1 CUZD1 ALPP ZNF343 FAR2 PPP1R12A RBPMS UHMK1 ATP10A TADA3 SPATA19 LY6G6C TRAPPC2B SULT1A2 ANKRD52 FBXO40 CORO1B PARP11 NKG7 CTAGE15 SLC39A12 IFI27 POLA1 MRPS18A ECE1 TCEA2 ZC3HAV1 SH3D19 CPA2 GABBR1 YIPF6 NR2C2AP ARPC5 PLSCR4 KCNK10 NCOA3 CYP19A1 TLNRD1 SLCO1B7 TRIM40 STEAP4 HOXB5 C11orf71 KSR1 SLC27A1 HMX2 PAFAH1B3 CTAGE6 CARD9 RDX PXN ANKS1B APBA2 NFRKB GDF2 SUB1 KLHDC8B PDSS1 AKTIP HLA-DPA1 ATP2B2 PLS1 PDE8A ANK2 AGO2 MAPRE3 FN3KRP GGPS1 RER1 LARP1 DBT BRINP3 ZBTB16 PDGFB ANKRD22 RAB9B CADPS NAIP DLGAP1 ZNF221 UBALD1 DDX60 ADAMTS4 SHH MORN3 NPM2 NYNRIN RWDD3 B4GALT2 CLEC2A JAK2 GRIN2B CACUL1 SLC38A8 NR1H2 RHCE GM2A ETV4 SYNJ2 LRR1 ZFP30 ORAI1 ZNF578 ENKUR ZNF234 STX11 BEND7 ADAL KALRN LRRN4 ACBD7 BMP7 PSENEN CPM SPG7 BCAR1 MCM10 OXR1 ZNF362 INPP5B DRAM1 PPP2R2C TTC32 LRRC34 GSDMC ESRP2 NOX4 CST11 TLE7 VTN ACVR1C INCA1 SEPTIN9 GK5 SC5D ACO1 NEURL3 MYO18A WAS PPP1R32 NET1 PAPPA2 ESR1 ZFPL1 NACA C1orf210 SGCZ ACE2 MCAM H2AC1 STRN3 SLC12A9 TEX10 EDA2R DGKQ PDCD2L FAM107B GRHL3 CABLES2 TRIM42 DENND5B PLSCR5 GRM1 MUC20 CLIC5 KIR3DL1 GEMIN6 CD46 LOC112268355 CENPN CDC20 STYX C4orf33 DOK6 HIPK3 EPB41L3 PRSS3 CDR2L BCL6 UTP14A BCAR3 SPATC1 TMPRSS11D MANEAL SHISA8 ZBTB7C TP53BP1 MME PCNX2 CWH43 GREM1 ELP3 DHRS7B UBE2L3 FZD10 NR4A3 BLOC1S5 PELI1 AMER3 BHLHB9 BCL11B CERS3 SOCS3 ZNF444 S100A16 POLR3C C12orf57 ALPK1 RSPH10B2 MTUS1 SLC10A2 CHKA TMEM187 NUTM2F RNF138 WDPCP SPAG4 SMG1 IL13 CD86 LIX1 ACHE ADPRHL1 KIR2DL1 SPIN1 TBX15 PSRC1 RAPGEF4 TSPAN14 PANK3 GPM6A AMIGO1 FUT8 SLC7A8 ALDH3B2 FAM104A PALS2 ADA CPZ FOXA3 RPL36AL PDCD10 SRSF4 TMEM62 COL13A1 ACADS ZNF260 MED30 NXT1 NRTN PSME4 FGF14 EPHX2 CSRNP3 TRIM16 CDC7 HABP2 MED23 ATF7IP GALNT10 NAA35 PTGER4 APOF CD109 ANGPTL1 KLHL32 SRL PSTPIP2 RPS6KA2 ARHGEF40 KRTAP13-2 DHRS9 TEAD4 ZMYM3 OR8G5 TGDS FAM120C THSD7B FAAP20 SLCO1C1 ENTPD5 SUCLG1 KIR2DS5 KIAA0040 MYO1E KRT2 DDX55 ANKRD65 CD14 RIN1 ARHGDIB ACE CAPN8 KDM4A PSMA2 HACD1 C4orf45 DACH2 OPN1SW LYPD6B DUSP11 FAM111A IQGAP3 MYBL1 NCOA5 MUC22 COL3A1 ZNF610 IL19 COL9A3 FIG4 CTR9 SP7 KLF1 UBR1 KIR2DS4 ZNF140 UHRF1BP1 KCNK2 PAM UBE2NL TBC1D4 BBS2 GOLGA7B GRB10 KRTAP12-1 MOXD1 SREK1IP1 STARD8 GRAMD1B ZNF701 FLT1 SLC25A2 RPLP0 RNF220 NPFF TNC ZNF174 NOL4 PPP1R12B BATF2 FAM200A ZFAND1 CMTM2 LRP6 LHCGR CBLIF MYL12B CD276 F13A1 SRP14 OCIAD2 ANKEF1 KRT72 CDC14B GIPC2 KCTD4 LRRTM4 NFASC ARHGAP9 CCR1 CR1L ENTPD6 UBN1 BST2 ACADSB SYCP2L HTR1F INPP4B GMPPB BMPR2 GPR20 KLF7 MZT2B HMGN2 TEP1 SP100 DMKN CES3 MAP2K5 UBE2R2 MRM1 LILRB1 ADM2 ZNF229 PDLIM1 GLYATL3 ALOX5AP OPN5 KLF8 ID4 KCNJ4 TACC1 RIN3 ZNF699 PPP3R1 KIR2DL3 PAX6 AAMP TRIM73 NFIB SLC17A6 CDH16 POU4F3 CYTL1 BCORL1 PEA15 ZNF23 CFAP298-TCP10L NPC1L1 TENT5D IZUMO1 ANKFN1 STXBP6 LRATD2 CLECL1 GBE1 FARP2 ZFP36L1 TMX3 EXPH5 MAG RNF146 NELL1 GALK2 SNX9 TTC39A RUSC1 MBNL1 TRIM17 PURA ARRDC3 NAXD ERBB2 GTF2IRD2B FANCM CALHM3 TCAF2 NDUFV1 PLD4 RAB3GAP2 PDLIM4 ZNF302 ENY2 SRGAP2B FUT3 FIGNL1 ZNF226 APH1A COX18 KRTAP12-4 GTF2IRD2 ERP27 NPEPL1 MASP2 C1QTNF6 KRTAP10-7 DTYMK LEMD1 KRT38 NEMF KIR2DS2 RBM11 TRPM1 SHISA4 HNRNPM SEC61G KCTD6 LRRC70 VPS28 FNDC3A RPL18A DIAPH1 ZNF71 OPRD1 WNT8A UBXN11 PLEKHG4B TRPV4 CTPS1 EPHX1 GABRP SINHCAF ASB6 PVALB KIR3DS1 C5AR2 TNFAIP8 A1BG SF3B1 ICAM1 GOLGA2 ABCC5 RAPGEF3 TRNT1 ABL1 MAP2K7 DBF4B FAM156A OR9Q1 IRX2 TMEM164 TRIM38 BTBD11 MTFP1 CALN1 MMP26 ATP5PB LRRCC1 PAAF1 ASNS CABIN1 MRGPRX3 TXNDC2 C1D KIAA1191 TNIP2 PDZRN4 SPNS3 FAH SLC30A6 FBP1 RNASE11 EXTL2 C19orf33 SLC45A4 ELF2 BTBD18 SYF2 PKHD1L1 SLC26A9 EDEM1 NOS3 KCNIP4 NKAPD1 H4C4 VMO1 FAM50B SLC35D1 PDE10A IFNLR1 EN2 TM9SF2 TMEM144 METTL7A SH2D1B KRTAP9-2 NSFL1C FBXW7 NKX2-4 RHOJ FCRL5 INTS2 TMED10 FDCSP MTCL1 EIF2AK4 TMEM121 RUSC2 PDE11A PABPN1L KLHL29 TMEM17 TEX12 SYBU ABCA9 RNF214 TMEM87A OPRM1 ST6GALNAC6 RSPH10B XAGE3 LRRC1 DMAP1 CATSPERB TRMT12 TNNI1 DNTTIP2 CLVS1 ATF6 GPR85 IL16 VCL SLC7A7 IYD SLC35C1 KIF1A EIF2AK1 COL4A1 FBLN7 KRTAP13-3 C9orf24 PPFIBP2 ACVR1B LHFPL5 - KANK2 PSMD1 SLC41A3 TMEM242 RNF186 CSN1S1 PLEKHD1 LRRIQ1 TDRP COQ8B ST6GALNAC2 RAB40C OSBP2 ZMYND11 MPP3 PLEKHA8 ACAP2 ZNF525 FGF13 BUD13 UNC80 KIR2DS1 SPINK6 HHIP ETFBKMT ALPG DAGLA EFCC1 ADPRM DYNC2LI1 SCAF1 MMD LSR ICE2 RNASET2 DDX23 TYW1B ARFGEF1 SCUBE2 CFAP95 ABCF2-H2BK1 MAGOH RPL37A IARS2 P2RX4 EXO1 SOSTDC1 CCDC71L LRG1 TBRG1 CFB NRXN3 PDGFRA TRAK1 TMEM150A ADRB3 ADM RPGR DLC1 STAC TSR1 ACP6 KCNQ3 SEPTIN8 PPP1R35 C9orf40 NDUFA9 AKAP8 EFNA3 MVB12A SCRIB RSRC2 MUC21 RAB27A WFDC12 PITX3 CITED4 YBX3 KIF20A GRM4 CFAP119 CDC42SE2 GPM6B RNF130 LMCD1 TAPT1 ELP1 FBXO38 ORC6 TNFRSF6B GSTA5 GFRA1 KIR3DL3 SUN3 CGN TOPAZ1 MARCHF8 WARS2 PREX1 S100A7 TCF23 CXCR6 VGF TUBGCP6 KRTAP9-4 SLCO5A1 CALD1 CERS2 ADPGK RPL31 FRMD7 DEDD2 S100A7A GFRAL ITGA7 GMDS ANO3 TYW1 LYZL2 DEK TRAPPC3 ALB ARHGAP24 LGALS8 PTK6 C1orf94 CTSZ ZNF112 GPR68 ZNF385A UCP3 NR1I2 YARS1 C6orf47 OST4 GBP5 SNRNP27 C2orf88 SLC25A38 FTCD PMP22 APC IFNA10 IFT20 UHRF1 OLFM3 SNED1 FZD4 PRPF40B KRTAP9-9 MARCHF1 DPAGT1 IL18R1 RASGRF2 CLEC10A INO80 GOT1L1 AMOT TESC SLC14A1 ZSCAN22 CRAMP1 TTC33 ETV1 ADAM11 ATP6V0E2 SLC35E2A IRF9 BACH2 NRG1 MZT2A ADAMTSL3 PRRT2 UEVLD DMAC1 TRIM15 TGFBI MRPL30 RBM43 PGM3 CHRNG TRIM25 LEXM KIF3C CDH4 ITPRIP CISH SPINT2 RPS16 CSNK2A1 ABHD6 CRYBG1 NDC1 CD3E IWS1 MCIDAS COL1A2 DTNA TMEM208 SERPINA5 CELF4 TMF1 C21orf58 CDKAL1 ST8SIA6 STXBP3 FBXL13 ANO6 KIR2DL5A ITIH4 TBC1D8B GGA2 COL6A6 TRIB2 CD40LG LAPTM4B TNS2 APOC1 KLRD1 C11orf54 SATB1 NDRG3 NKIRAS2 NDUFA5 ECSCR CYP1B1 GRIA2 TOB1 ZNF17 TMA7 CENPF PIP4P1 ZNF440 ADAMTS7 ZFAT SMAD5 YPEL2 RHBDD1 COLQ PHF23 PSD3 GNL2 VPS13B ZNF471 FBLN5 BCL2L14 KRT32 HMGB1 TRIM50 CDHR2 PGK1 BICDL2 LZTS2 PALS1 OAT RNGTT ACTR3C MISP3 CDYL KCTD14 CDCA8 TP53TG5 ADIPOQ |

| Dataset | Number of genes | Gene symbols |
| --- | --- | --- |
| STAT1:  STAT2 | 2155 | ELMO2 CD44 SLMAP CEP250 SAMD4A DLG1 KCNMA1 PINK1 PIGX PKNOX2 TUBD1 GOLT1A RXFP3 CNTNAP1 CYP26A1 TUT1 KLHL13 APOL2 XDH ERAL1 ECHS1 ANKRD36B PBX4 LRRC41 CXCR4 CT47A9 NUP155 CYP39A1 RBM15 SSC4D SIDT1 MMP7 ZEB1 HRG ZNF131 SP140L USP47 LRMDA SYCP3 FGR NDUFAF4 HLA-DRB4 CAPNS2 GDA SEMA4D MREG HEPH MET MS4A10 HLA-DRB5 BANK1 TBC1D3H GPR143 TMEM39A HOXA1 OSBPL8 RCL1 RRP7A YEATS4 FADS1 TSC22D1 ATG2B MAPKAPK2 HMX1 EPS8 SHC1 GEN1 BRCA1 CDC25B TMEM19 LIMS2 EIF4G3 A2M PIK3R5 CDK20 UTRN CDK11A TRIQK GRPEL1 SDAD1 TBL3 TERF2IP UGT2B7 FOXK2 CLTA ZMYND15 CCDC106 RNF32 ATL2 DGUOK CLDN16 FGGY RBM17 CD1A LYPD4 SSTR2 IFNL1 PRR16 ARL14EPL SLC1A7 LGI2 CAVIN3 NEIL1 LTBP4 CCL19 ARHGEF9 GPR55 MFF SLFN12 CLEC4E STK17A CDKL3 CNGA3 GLE1 DHRS7 ALX1 CCNE1 ZNF257 EVI2A EXOSC7 PRAMEF15 APOE CABP1 H2BC21 ZNF484 GMPR C3 ING5 ALG2 HDAC7 EPB42 ADGRF1 MAPK12 PIWIL3 SIPA1 CCAR1 EP400 PTK2 BTN3A1 GSDMA LAMP3 TOR3A MFSD11 C8orf44-SGK3 USP7 OTUD3 SPINK14 HNRNPR LCMT1 MEIS2 AADACL4 CPEB3 RPLP1 ELOA2 HESX1 SAMSN1 SH2B2 RWDD4 THADA PIK3CB ATP1B4 FCN1 RAB3A PRAMEF7 A4GALT COG1 FBXO24 DNMT3B DNAH11 DHFR2 SLC9A9 PLAAT2 RGS4 CT47A11 SLC13A4 EDNRA TMEM200A ZBTB11 ZNF331 CEP120 PDE4C SPTBN1 SPX FRA10AC1 MRPS12 TACR1 NME1-NME2 CELA3A GRIK2 IGSF11 STMND1 MRLN LTBP1 LRRK2 PPBP PHLDA1 ACOT7 MAPK10 RIIAD1 GET1-SH3BGR LAMB4 EZR MAN2B1 ZBTB18 GPHB5 TMEM33 SLC22A14 AHCYL2 KRT37 GLIS2 NACAD DAPP1 TMEM125 CNTN2 C12orf4 GCA NDUFC1 TBC1D3E PABIR2 RP9 RARB MRPL15 CEL CNOT6 ZBTB20 RPUSD3 STK40 AGGF1 MAK16 IPO13 DPF2 TSN YIPF7 UGT2B4 DCUN1D5 TCF4 ABCC11 NR2C2 SPATA4 CEP85 CHMP7 CCDC172 RHOBTB2 IL27RA SLC3A1 AAMDC TIAM1 RPP14 CT47A4 ACTR5 NDNF KRT15 GFI1 FBXO36 GOLGA8N AHR SYTL1 SPATA19 PHACTR1 MLIP IRAG2 BCAP29 DGKD GPR132 LOXL1 NMNAT2 SORBS2 IQSEC2 IGSF8 MUC15 PRRX1 KLKB1 RBBP6 IFI27 GNAO1 IL1RL2 TAGAP ECE1 OLFML2A C1orf112 HAPLN4 LIPI PTGES3L-AARSD1 GABBR1 ATP7B LANCL2 PPM1H LAMB3 CEP131 MAP3K7CL CARD14 VIT SEPTIN7 ASXL2 RNF182 FSTL5 RALGPS1 CCDC6 CYP19A1 EIF4E2 NPFFR2 PRSS22 CCM2L S100Z FHDC1 HOXB8 WARS1 ZFP36 NDRG2 PXN RAD1 RILPL1 CCDC77 TNKS2 APBA2 FANCA DNAJC11 DEUP1 ASAP1 PCBP3 MEIS1 KLHL10 SHLD3 AMMECR1 DLST HAGH NECAB2 FBLL1 FYN TLK1 TBC1D3B AQP8 CFAP44 PI15 FAM9B BRINP3 PMVK ZNF649 NUGGC SGK1 DDI2 POLR3G PSMD8 CFAP43 DLGAP1 TANK SERPINB13 KIT MEMO1 ZNF518B SYDE2 EPHA7 TYRP1 DSE RAB20 TNFRSF17 OSBPL3 POLR1A TMPRSS13 ETV7 METTL5 CORO1C CD8A PRRT1 SUSD6 LRP1B XAF1 GLI1 SDR16C5 RGPD2 ZNF157 MCFD2 EPS15 GRIA1 XIAP SPDL1 ZDHHC22 SECTM1 ACBD7 B2M UBE2D3 SQLE POLR1F PPFIBP1 NELFCD AGER GALNT2 ZNF362 ATXN7 STK3 TGIF2 VEZT NCSTN ICE1 BMP15 C1GALT1 OXA1L IL4I1 LRRC61 SLC9A2 AGAP4 PSIP1 ZFP90 MEIOB TOR2A USP18 ATXN2 ZSCAN12 RASSF9 ANP32A DNAJC1 ESR1 GCNT2 PIGBOS1 SURF4 KCNN3 GLOD4 FKBP5 SPATA9 PCED1A SPDYE1 CCAR2 MSS51 CMKLR1 DNA2 SCNN1B ECHDC3 SH2D3C KCNAB1 SRRT TRIM42 APTX PDE4D MUC20 EPHB3 SNCA AQP12A LRRC45 CLIC5 EPSTI1 KCNMB3 ARSK HSD17B7 BSX RAB44 RIMS1 EPB41L3 CTU2 KAT5 CDKN2B FGFR4 POR NYAP1 MORC1 ABRACL TRIM14 APOL1 PIK3CD OR10H2 SHISA8 CDS2 SLC6A16 DOCK4 CCDC14 PAX2 EFCAB12 SGO1 DHRS7B BLOC1S5 DNAAF5 MCTP2 SPTLC3 BIRC2 ART4 UNKL MDFIC2 GPR18 S100A16 DCAF8L1 PRAC2 PCDHA7 SLC9A3R2 LCE1E MACIR NELL2 CHPF2 TRAPPC6A TSPAN2 IL1RAPL1 VCP SLC23A3 LAMA3 PPP1R3F APOBEC3F KRT78 CALM2 TRMT9B CTSF SLC25A39 RALB KIF1B BNIPL AMOTL2 FBXO25 PSRC1 TSPAN14 TGIF2-RAB5IF CLIC1 MATN4 LILRA5 IBSP PRSS41 AMPD3 ST6GALNAC3 SPINK13 TBC1D3 TMEM62 TNFSF13 GTPBP2 PRR20E CREM CER1 PTGER4 METAP1 CCR7 CD68 EPCAM ANGPTL1 GOLGA8J XKR3 FHOD3 CR1 PRDM1 SLC26A6 TMEM89 BBX EFEMP1 RFK RERE FOXG1 P2RY14 TRIM24 PPP1CA ZNF608 SLC22A5 SLC38A6 TBC1D3I ADGRG1 GRAMD1C H2AB2 FAM83A ARMCX3 ZFX KCNH1 ITGA10 CDK18 HACD1 IFT74 KLHDC8A STK24 ACSS3 CNTROB MUC22 IL19 IHH PDCD6 FAM3C ADARB1 BRI3BP EIF2AK2 ASB4 IDO1 PPY HNRNPH1 ZNF563 BPGM SLC23A1 ARHGAP32 MDC1 FAM126A GRAMD1B NKAIN1 PKIG IFNE PPP4R3C RB1CC1 POLR3A ADGB EBI3 U2AF2 EXOC4 LALBA NOL4 UTP25 AXL CRABP2 RINT1 IL17RD KLHDC7A CDC14B LOC102723623 FNDC9 SDSL MARCHF9 ABCA10 PRAG1 ZNF493 RAD23A FAM83B HDAC4 MT1E RPS6 CTNNA1 FAM102A TECTB PCLAF C9orf153 GHITM FOXN3 CCDC3 SP110 C1QTNF9B TTC36 THEM5 TFB1M FNIP2 GOLGA8Q TRIM34 GADD45A DKK3 FYB1 ALOX5AP AASS CBLL2 SHQ1 JAML ZNF16 TACC1 SERTAD4 CCNJ CFAP91 SH3YL1 MS4A4A PLAT SNX18 ITGBL1 SPOP KIF12 NDST3 ABCA6 NBEA EGFR FRG1 POLL TYK2 SESN1 CLASP1 CMC2 PLGLB2 UNC5C AFAP1 PRKD2 BCR CXCR3 PUS3 DRICH1 DZIP1 SEC22C OR2L2 CHI3L2 ZNF317 MTMR4 ANKRD36C WDR74 GCSAML RAB40AL PCNP TBC1D3G TMEM196 ARHGEF28 C9orf57 PRICKLE4 RMDN1 DDX3Y MXRA7 RIOK2 TASOR SRGAP2B NAAA CLEC7A SLC37A2 PIF1 TRDMT1 IL17F CDT1 UMAD1 IL4 APOOL SELPLG DIABLO TGFBRAP1 MUCL1 CLSTN3 GCOM1 GBP1 TENM3 TMEM181 TTLL9 PRR20B CCNB1IP1 ZNF408 CCL3L1 AKAP12 AKR1D1 MEOX2 FAM71E1 PSMD6 MGAT4C SH3D21 AKAP3 TUSC3 OAS2 IPCEF1 OR52A5 NACA2 ANKRD36 ZUP1 UBXN11 TRPV4 EHD4 VCX3A NOX5 C1orf167 PPP2R2A H4-16 GOLGA8K C5AR2 STARD4 CT47A5 ACVR1 RNASEH2B LPCAT4 TMEM52B RFC1 HCFC2 CMTR1 BRDT PLAC1 TMPRSS7 KLHL18 CALCR DPF1 PATL2 CDC45 DNAJC21 SRD5A3 CALN1 RGS13 PSMB4 ELMO1 DIO2 LRP2 CYTIP CYBB UXS1 SPTA1 PAAF1 GEMIN2 PMAIP1 SEMA6D RAD50 GOLGA6L6 MTHFD1L NTF3 TRPC6 METTL26 GSDMB WDR44 IL25 HRH1 GLDC EHMT1 AMDHD1 TTC29 RAX GDF11 NEURL1 MCMDC2 ZNF415 ZBBX ZNF585A RPL9 EGLN2 CCDC184 SLC24A1 LRSAM1 CSPG5 RNF133 PTPN6 TST POP1 H3C15 PAN3 TNIP1 CELF2 GLRX CD70 ST3GAL4 TBC1D3D TASL FBXW7 HEY1 CYB5R3 TBC1D32 C1orf105 RBFOX2 SNRNP35 OSBPL9 ZER1 SPATA6L ZNF804B PRAMEF20 PRPH FDCSP FAM9A FBXL7 POLR2A ZNF169 GLIPR1L2 ZNF730 CDK11B SNF8 CT47A10 SYBU TBC1D22A STX10 NADK TRMT10A TRPM3 ZNF559-ZNF177 STK32B FLRT3 TYR IL36RN SLFN11 HERC5 MYZAP ICAM3 PCDHB12 FFAR2 SLC1A3 GLI2 BTNL2 CCL5 GOLGA6L1 RBM41 CCDC69 RAPGEF2 EIF1AY RNH1 CCDC163 PLGLB1 ADGRF3 ENTPD1 RAB40C SLC41A1 GDE1 LRCH1 MX1 PRR20A PURB PLA2G4D C16orf87 FIZ1 FGF13 RELB FNTA HNF1B DTD2 PBXIP1 PDCD1 CYBC1 TMEM229B MARCKSL1 SPSB4 GPR32 IL32 DGKE DNAH3 PLPP1 ICE2 PTPRM ARRDC5 TRIML2 TMEM59L EIF2B1 SLC6A12 APH1B EVA1A NONO F5 GOLGA6L22 G6PC1 RPL37A PPP1R11 NREP CPS1 GCNT3 NRXN3 PIK3R1 TRAK1 CUTC PTPRJ FGD3 TBC1D3K UCN3 IL17B MLKL DLC1 CCNF LAP3 SRM PRKACA LRRC46 PPP1R1A NOVA1 OTULIN IFIH1 TUBB4B NUTM2E XRCC5 AHSG RBM39 ARHGDIG KLC2 SH3RF1 MICAL1 SH3BGR VRK3 FOSB AREL1 PRM2 MFAP1 NEURL1B ASF1A TDRD7 UBFD1 CDKL5 HLA-DOB VGF WIPF1 UST ATP8A2 ST6GAL1 SIAE PMS1 PSMB3 KDELR2 SNRNP200 ZBTB3 TSTD2 SOBP SHOX2 ST3GAL5 RNF114 ANO3 KCNIP2 ZNF821 TSPAN4 GOLGA8R SFT2D2 GRAMD2B TMEM61 UPF2 ARHGAP24 ALB CPT1A CEACAM1 KIF22 DCN RNF11 NOD2 CERS6 FAM47E PRELP UBTF SLC13A3 HLA-C PLA2G12A TIMP4 GBP5 MKS1 MYLK3 BAALC SLC30A3 MORF4L1 HSPB7 NKX2-2 CNNM2 CCN5 LOXHD1 HMGXB3 FUT2 ANKRD7 SLC39A1 LIN28A DHX58 CT47A1 KCNJ11 CASP4 TSFM MAP1S IL18R1 AKAP11 CCER1 BCL10 MAGEB10 HLA-DRB3 MAGED1 TMTC1 GSTM2 FAM71D MOCS2 CTXN3 NAT1 CDC20B SPTSSB PARP9 MT2A ROR1 KLHDC9 PPP2R3A ZFY PPARGC1B MIEN1 PTDSS2 STON1-GTF2A1L IVL POMGNT2 H2BC3 HNRNPU CYFIP2 DYNLT3 MAP2K3 NME1 SLC4A4 ESR2 MESP2 KAZN NEMP1 FCRL1 GJB4 ANKRD34C ZCWPW2 KCNJ8 KTN1 ADARB2 GJA5 EVL BAIAP2 SLC17A5 SI TRIM5 PYCARD BAAT PRAMEF8 SIGLECL1 RRAGB PDE4A NKIRAS2 SLC5A8 SCO1 NDUFA5 NCLN LYPLAL1 ZNF251 ABLIM1 THNSL2 ZNF445 TMEM54 SLC19A2 PHF23 AP1AR LTA4H DUSP13 BCL2L14 RFX2 CHRNA7 ZADH2 IFIT3 ADGRE1 MEF2C HOPX METTL1 LZTS2 PGK1 CCL13 IL2RG DRD1 CDYL CEP295 DERL3 ESM1 TBC1D19 HMCES CCDC25 ZNF416 STT3A TSPAN11 SLC6A2 PDE6A C3orf85 LPP SPINK1 YIF1B ATRX RAE1 EIF4EBP2 DMRT1 HSPB6 FGL2 CEP70 WASL MPZL2 CHRDL1 CCDC59 CTNND2 MBNL2 ARSF ANXA1 FRMD3 TAF4B ZNF189 APOBEC4 APOBEC3D RAP1A SETD2 SENP1 TUBGCP3 FCRL6 ZNF783 SVBP POU4F2 ACOT1 RFX5 HOXC13 TXNDC15 PTPN18 TFDP2 TOPBP1 CTNNBL1 TMOD4 GNAI3 NUP214 DPYSL2 PTPRE DOP1B GOLM1 AGAP5 FPR3 SOX4 FBXW8 PTPDC1 CEP135 TRIM22 NLRP3 RC3H1 ATAD2B BTNL9 SFXN2 VEPH1 KIAA1522 FGFBP3 URB1 RARS1 TCIM FGF11 ACVRL1 FARSB H3C10 ZYG11A NFATC4 CENPBD1P DEFB1 ALKBH1 ARHGEF7 SLC23A2 GRK6 MANBAL STX2 ARL14 TIMP3 LIN54 ARPP21 NR1H3 MPPE1 SLC44A2 MOB3C RGS3 MAML2 TRIM43B DSCAML1 RAC2 GLUD2 RPP40 TRIM60 ADAMDEC1 LNPEP APCDD1L NAPG UCHL1 ZNF669 ZBTB22 YIPF4 APOL6 MEPE ANKRD10 KRT39 SLC16A7 TAGLN2 OR51Q1 DAB1 CARD18 PHF3 THAP11 MRPL19 DICER1 DPP4 KLHL23 CFAP61 KCNA2 CAPZA2 TEX19 TBC1D5 SFR1 DACT3 DOCK8 CACHD1 GAN ARL5B MUC16 ZFP37 MRPL33 GREB1 KIFAP3 BTN3A2 LILRB4 ARNT CNKSR3 BDNF PRPF19 R3HDM4 ZNF574 CYSLTR2 RASAL1 C19orf25 COMMD2 YEATS2 ABCA1 PLSCR2 CLEC2B TNFSF10 OR51J1 SCRN2 COPA CKB IGSF1 PRAMEF26 PCDHB8 ATP13A3 STOML1 EXO5 MUC7 CCSER1 MRGPRX4 C8orf48 TNFSF15 NCF2 SUPT5H NUDT7 DCLK1 ZNF665 H3C14 RAD9A MAGI3 TSEN15 HDDC3 ARHGAP45 TMEM161B CLDN22 FKBP14 RNF145 HOXB3 ZMYND8 CDKL4 GATAD2A PDXK SMLR1 CASP8 RCOR3 NRIP1 ETS2 BNIP2 DHX30 PRR20C MED10 FGB ACP2 PIWIL1 RCAN2 LRRC69 ADGRL2 CAVIN1 CCNB3 CCDC196 BAG1 COLGALT2 CORO2B DHX40 NUPR1 ITGA8 F8A3 CIAO2B SYCP1 RBMS2 TMPRSS6 AKR1C2 ANTXR2 TNNC1 IL31RA BCL9L ZP1 RUNX1 HSPA4L ZNF765 MID1 C2orf81 CTAG2 TRIM43 FBXW2 ZNF540 HDAC9 STON2 WIPF3 LITAF ZNF410 IFNL3 FAR2 PCDHA2 FBXO33 CPB2 TRAT1 PLAGL1 PIM1 IFIT2 DYRK2 NR3C1 CYP4V2 CHKB TM2D3 CLEC4C MAP2K6 HUWE1 FAAH EARS2 RRAS2 LEPR SH3BGRL H1-3 ANKRD42 SYNGR2 NDUFS2 SLC19A3 LSMEM1 ZNF502 KDM1A TLNRD1 CCDC170 EYA1 CCNL2 MYPN RORB MIIP NHP2 KCNJ9 LRIG3 KIAA1217 CSK C2CD4C TAS2R19 MORC3 MORF4L2 MRRF PATE2 CDH18 PPHLN1 RAPGEF1 PRKAB2 CNTF ZIC1 FURIN RHNO1 MPV17L2 ZNF281 SMARCA5 PLEKHG3 TMEM14A CWF19L1 GPKOW BLM ANK2 CHST4 SENP6 H2AB1 GGPS1 PPP1R3B SRBD1 FHL2 RNF19B KCNMB1 PCDHA9 RTN4RL2 PCNA LMO2 AMY2A BEST1 UNK DDX60 HLA-A SUPT3H ZRANB1 MAN1C1 YDJC KRTAP13-4 HSPA6 PTGES3L ZNF518A NT5DC4 GM2A ARHGEF39 S1PR4 ETV4 MS4A5 ANXA2R PPP2R5C PCDHB4 KRTAP10-6 TRIM26 AK9 MBD4 MPZL1 IL34 CRISPLD1 STX11 TBXAS1 DNAJA1 GPR176 MPP2 SCGB1D4 INPP1 MATN3 POMK PRKX KIF5A PKP4 SLC44A1 EGFLAM GUCY2D SHPRH NPR3 TBC1D3C VPS11 MYO5A CNNM4 C5orf49 TRIM27 SIPA1L1 OXR1 PAK3 PNPLA1 FBXW9 SLIRP CBY2 VPS33B TBX1 SALL2 EBF1 ZNF546 POMGNT1 MPHOSPH8 CYP2E1 TNKS CT47A2 KDM8 ATP1B2 MTIF3 NALF2 TIE1 PAPPA2 GTF2H4 LAT2 ITM2C LOC645202 TINAG SCAMP5 EREG GSTT2 TXNL1 ABCB5 H3C11 TRIM69 LRRC14B TNFSF18 PCGF2 CYYR1 PNRC1 IL1RAP UBXN2B CASP7 CCNJL PER2 ARFGAP2 ZKSCAN4 CDC20 UGT1A6 STYX DOK6 ADRB2 PMFBP1 KY CD3D ERICH3 AFM TBP CCDC40 DNAJC18 RIOK3 SCARB1 CXXC5 GCHFR GLI3 MSANTD1 PTCH1 ERCC3 H3C8 TADA2A NEDD1 RPL6 GJB7 LRRC9 PRKAG2 SNX20 AGXT2 GOPC FRMPD2 PNPLA8 COX4I2 SERPINF2 FMO1 JAZF1 TCF24 DCDC2 C7 DYNC1I2 HAVCR1 FANCF MTUS1 ARFIP2 SRP19 HYDIN ZBP1 DHRS3 AIF1 PYM1 SOAT1 MRPS30 EXOSC5 CHODL MIEF1 PLAA AKAP4 ABHD17C INSYN1 ARNTL C20orf202 KCTD9 CTCF SIGMAR1 MTNR1A HEG1 EHBP1 SNTB2 PDIK1L NIM1K ALDH3B2 ZSCAN1 AGAP2 CCND3 COASY SPATA7 M6PR SRP9 GPR88 EIF5AL1 TLR3 ZNF761 MS4A6E C22orf31 TRAPPC3L PRAMEF11 FGF14 PTAR1 STATH ZMAT3 BATF PCNX1 H2AC4 ATF7IP UGT2A1 MVP HHAT MRGPRX1 UBE4A TMPRSS15 MX2 PNLIPRP2 CREB5 SHF FAM120C ASB15 CCRL2 TRAPPC13 PRAMEF2 TAMALIN PLIN1 RHEX SH2D4A SLC7A2 ZFTA SEC23A ACE CPA6 CEP57L1 PRKCE DACH2 KIFC3 IFNL2 TBC1D3F ZC3H13 CREB3L2 GPR82 ACYP1 FOXP4 TK1 MFSD2B RTN4 NAPA GOLGA8M LETM2 HLA-F ARHGEF11 CD37 DUSP19 KCNK2 VCX3B DPT BTBD3 GRB10 MMP28 ADGRD1 PCDHA10 ASXL3 ZDHHC11 HMGN5 NIPSNAP3A EDIL3 SCAPER INSIG2 PCDHB7 FASTKD3 RMI1 ECM2 SHCBP1 TSPAN15 CLEC3A VPS26C LATS2 IKZF2 GUCA2B DLG2 OR51H1 SESN3 PPP1R12B CT47A12 DSC3 PDE2A TBX4 LRP6 SPDYE2B AFF2 RFC2 VWDE KRIT1 CD200R1L ALOX15B ANKEF1 CD59 DENND2C STXBP5L TBC1D13 ARHGAP9 CACNB4 TNFSF4 STX17 HERPUD1 H2BW1 ACAD9 RBP4 AARSD1 SERPINE2 NME2 GCC1 SLC36A1 PPEF1 LOC100132202 KCNH8 CPEB2 IGF2 TASOR2 CUL5 NUBPL H4C12 EPB41L2 NOD1 KIF6 NUBP1 ADAMTS1 PRR20D P2RY6 ZNF354B ELAVL4 AKAP1 RIN3 SBF2 CDH9 CCDC191 NFIB PIK3CG SORBS1 SLC15A1 HTD2 MB SNAP23 CBL RPL5 GSTT2B FFAR4 WDR25 SLC35E4 GOLGA8H CT47A8 LCE3D CIC NELL1 ITIH6 PCDH19 TSR2 MBNL1 GRIK5 DCUN1D4 DAZL SLC16A13 PLCL1 ATP6V1H ZNF322 NECTIN3 ALDH3A1 IFIT5 FAM177B PXDNL ZNF226 ZNF69 IFIT1 CALML3 APH1A WIPI1 NAT16 OGFOD3 ASIC2 DCTPP1 ZNF777 GNPNAT1 KRTAP10-7 GRM5 DNAI4 FGF2 PAMR1 FXYD7 CCDC33 ATG4C IRF5 R3HCC1 PFDN6 TFAP2C COL2A1 ABCB10 TMEM245 SPRR2A EEF1E1 AKAP9 LGI1 C19orf48 NCR1 GPSM3 MRPS23 MTRF1L RIPK1 UNC93A TNFAIP8 PPDPFL IFNA13 TRIM54 H4C15 NMNAT3 RNASEL TMEM260 GBP6 GET1 AQP12B CCL28 PRR5L TRIM38 PRR7 ACAA1 TMCC1 MACROH2A1 ELSPBP1 FOXF1 PCDHB16 NCAPD2 CRYZ PRSS23 ANK1 DNAJB9 CTBS ISG20 CASZ1 LRRC37A3 ANKRD29 KRR1 SMOC1 SLC30A6 SH2B3 SCRN3 RABEP1 FRK BTN3A3 TMEM116 RELA HSH2D SCAMP1 BANP EID3 NIPAL3 MAP2 OGFR PROM1 MS4A4E TMPRSS11F MROH9 KCNIP4 TUSC1 CAMK1D GOLGA8O KLF14 MED28 LYPD5 OR52I2 C19orf54 NFATC2 DST SLC17A2 PRAMEF27 GDI2 PRAMEF25 FDX2 NKX2-4 DSG3 CRYGS USF1 ETAA1 ALOX12B TSC22D4 USP48 FAM189A2 APOBEC3C PDE11A RAD17 INPP5D GCNT4 CLEC12A CD5 MAP9 MRS2 TSPAN9 DOP1A H2BC8 COL17A1 KLHDC10 OPRM1 MAT1A TKTL2 CALM3 TRMT12 NMI NKAPL B4GALT7 NSMCE3 PCDH9 H4C14 OR1F1 CCDC61 IPO11 SOCS2 NKAIN2 CT47A3 AKAP5 SPATA21 NUP54 CD96 RBMS3 - CAP2 STAT2 C6orf201 EDARADD PRAMEF4 CD1E ACTG2 FAM83D SRFBP1 SLC29A2 IFT81 NHSL1 SMG6 INSR ACMSD SRGAP3 AKAP13 DDX60L OAF ZNF337 MDN1 HLA-DRB1 ETFBKMT FOXN1 AMOTL1 ZNF607 DYRK1A KLK10 RADX KCTD7 CPA5 HLA-B TMEM30A PCDHA1 TCF7L2 NEDD9 ETV5 JAK1 SLC39A11 ARFGEF1 GALNT11 NDUFA3 PPP1R1C NRCAM FABP4 SLC35E3 RHOBTB3 CFB DDX58 TTC23 FCN3 RIMS2 PTPN3 GBA3 MCM6 TMEM150A ART3 SART3 UPP1 FOXP1 APOBEC3G CUEDC1 USP22 STAC CCDC85A CTPS2 NDUFA9 TJP2 ZNF888 HSD17B8 PSMD7 GPM6B TMTC4 CCL25 AIM2 IRF8 ORC6 SLFN5 SPDYE2 SPATA13 BMPR1B DUSP15 CARF CNPPD1 SREK1 ADH6 TTPAL RSC1A1 OAZ1 GOLGA8T OR4N2 NUMA1 TCF7 RLIM S100A7A MMRN1 ARID4B RGPD1 UBE2V2 FASTKD1 ACTA2 ZNF770 SPATC1L DERL1 BECN1 TRMT10C ANKRD1 IFT57 DDR1 FKBP9 PRSS36 ALDOB CARD19 ZNF579 FAM209B ZNF385A SYNPO ATE1 C10orf82 PEG10 TDRD1 PRAME CD69 MFSD14A TEPP LARS2 GNGT2 IFNA10 DBR1 GPC6 UBR7 RAET1E BCAT1 WNK1 SGO2 GSDMD ARL17A MAGEB1 OLA1 LARP4B FAM120B CHAC2 CD300E CEBPB ST7 LRCH3 TRPS1 SSBP1 PDIA4 ANXA4 OR51I2 YTHDC1 ECI1 TULP3 APBB1IP ADGRG7 POM121 H2AB3 AOAH PNN SYT9 MTMR3 TRIM25 OR5T1 LEXM EPB41L5 RNF212B TMEM168 RND1 GABRA3 ZNF33A PHTF2 KLHL3 APOL3 USP44 ADCY9 SLCO4C1 CD274 MAP3K19 GORASP1 MSANTD2 FCHO2 EIF3G TNS2 SRF SLC18B1 MDH1B C2orf74 C2orf66 PGPEP1 TRPC3 KLRD1 PRODH2 RNF44 ZNF383 TP63 JCHAIN APP GATA4 IFI44 ZPBP MS4A14 TMEM270 WASF2 DNAJC13 EAF2 HDAC6 DCLRE1C SPART TTC30A HAPLN1 BNIP5 ARNT2 KCTD1 BIRC3 TRAPPC6B GPLD1 EXOC6 AQP7 TP53TG5 |
|  |  |  |

| Dataset | Number of genes | Gene symbols |
| --- | --- | --- |
| STAT3 | 1264 | KRTCAP2 CLPB HMCES SLMAP PLCE1 ARHGEF10L KCNJ2 DNASE1 ZNF225 GAPDHS TSPAN11 NBAS APOL2 YIF1B TACC2 RAE1 AKAIN1 PDCL3 THEM6 NMB MACF1 IRAK1 GSTA1 SLC35G3 POLR2L GALNT3 DDB1 GDF15 TAF4B DCAF5 COL22A1 GNAL MITF TICAM1 C1QC LYG1 KCNA6 DDX11 TAF9B MTM1 PROP1 DCLK3 ANKRD13B ANAPC4 SLC38A5 NLRC4 PPP1R13B KCNF1 LRMDA CCDC28B KCNE1 SLC16A14 KLHDC4 SLC4A8 LYAR SDCCAG8 ZSCAN5A KLC4 GPR143 FPR3 OSBPL8 RNF41 ZNF181 TNS3 SPIN2B GK MFSD6 ALPK3 CHID1 SLC9A5 MTMR1 PTCD3 NRP1 ACAD8 STRADB SAMD3 CACNG5 MAPKAPK2 ATXN10 SPPL2B ARL4A MAB21L3 CDC25B BMP3 ACVRL1 MECP2 PNPLA4 TMEM60 CNBD2 ARHGEF7 NOS1 ATP5MG PIK3R5 TIMP3 ARPP21 SNX16 ABCG1 MOB3C ZNF704 LRRC8C GAREM2 FGGY ZNF197 PRAMEF13 TRIM60 INTU SPAG11B YOD1 SMAD3 IFTAP TRAF5 XNDC1N TRDN SSTR2 SHISA3 LOC389199 SLC1A7 APOL6 ZNF529 SLC2A13 GASK1A ANKRD6 SHROOM1 SEPTIN11 TAGLN2 NEDD4 EZHIP YTHDF3 PCDHA13 SPATA17 PEX14 ZBTB38 DRD5 HIF3A ZDHHC6 FGF17 PODN LAMTOR2 TEX19 TBC1D5 GPR78 HRH4 GSTA2 ZNF385B MAOA CASP10 DACT3 PLEKHB1 SRI HLA-E ZFP37 C5orf15 HDAC7 MAPK12 PIWIL3 ZNF596 PRPF19 SOD3 C17orf99 RPS28 THAP12 STEAP3 KRT26 PANX3 SLC35B2 ZNF25 TOR3A SAMD13 FNDC1 LRIT1 NABP2 ALKAL1 HNRNPR MEIS2 GCLM IPO8 FAM167B TMEM120B HCRTR2 ELOA2 ASIP PHC3 SLC35D2 CD36 OR4M2 FAM76B KIAA1143 MAEL ZNF284 FAM162B ARF4 CSDE1 RAB3A ATP11C SLC12A6 ELF3 H4C13 ZSCAN4 TOX3 PIK3C3 PAX9 NOP2 SLC9C1 SPOPL RGS4 RCSD1 FOXD3 CXCR2 ZIM2 POTEG IFITM5 PRR22 ZNF24 RHOH RANBP6 RAI14 CYP1A1 HOXB3 RNF145 HHLA2 AKR1B15 ZNF547 SNX25 VSNL1 GSAP SHOC1 MBD5 C9orf163 PRKD3 TAP1 ACOT7 N4BP2 RASSF5 ATP6V1G3 ITPRID1 RIIAD1 STRIP2 FAM24A MRPL54 FOLR1 LAG3 AGT GTPBP3 FAM156B TCF12 POU4F1 STX19 GNG12 CFAP298 PTPN20 PLEKHG2 MTMR8 COL7A1 DSG2 ISOC2 ECEL1 TBC1D28 HSPA4 NDUFC1 ZNF79 CD99 SLC7A14 NEBL RARB SLC1A5 ZNF227 PRRT1B LYSMD2 CXCL11 PICALM IDNK DTX1 DRAM2 KRTAP19-6 PNOC UBE2L6 C11orf58 ZNF407 SUN1 LDAH CCR3 MED12 ABCC11 AGTPBP1 ATP2C1 KRTAP10-2 ALDH1L2 DOCK11 FAM237A TSHZ2 PABPC3 DUSP26 HDAC9 CD58 RLN1 C11orf68 KIAA0586 TOM1L2 CPAMD8 MLN AAMDC DLX4 IL18BP OSGEP SMC6 CNR1 MYLK2 ALPP ZNF343 PPP1R12A VSIR UHMK1 ATP10A MLIP TRAPPC2B PIK3R3 ANKRD52 CORO1B CTAGE15 ZNF747 IFI27 C11orf53 CCL27 KLRC1 POLA1 SMIM10 MRPS18A ECE1 TCEA2 ZC3HAV1 YIPF6 NR2C2AP OSBPL10 ARPC5 DCX RRAD H2BK1 ILK NCOA3 TRIM40 STEAP4 SLC25A18 SLC17A9 HOXB5 C11orf71 SCAMP3 KSR1 SLC27A1 MYPN PHETA2 HPR PIEZO2 CDK6 RRP36 ADRB1 KIAA1217 CTAGE6 CARD9 RDX TUFT1 ANKS1B GRIP1 GDF2 SUB1 DLG4 CHAMP1 KLHDC8B PDSS1 AKTIP FURIN ATP2B2 PLS1 FRMD6 CPVL SULT1A1 ZNF419 ANK2 LMBRD2 KCNE5 MAPRE3 ESPNL RIMS3 FN3KRP SPEF1 GGPS1 SRBD1 BRINP3 AVIL KIAA0930 ZBTB16 PDGFB RNF7 ANKRD22 MPI NUDT6 PCDHA9 FGFR2 DLGAP1 ZNF221 CERS1 UBALD1 MMACHC FNTB ADAMTS4 SHH FHIT NYNRIN MAN1C1 RWDD3 RBM7 CLEC2A GRIN2B NR1H2 RHCE SYNJ2 ST3GAL6 ORAI1 ZNF578 ENKUR ZNF234 STX11 BEND7 ADAL LOC102723475 XIAP AQP11 WNT5A KXD1 MPP2 GNS LRRN4 NF1 PSENEN FGF4 NPR3 CPM MCM10 IER5L FIS1 TTC32 LRRC34 SLC6A13 HK1 ESRP2 SFMBT2 GAS8 TLE7 ACVR1C INCA1 SALL2 SEPTIN9 NINJ2 CCND1 GK5 MPHOSPH8 ACO1 HNF4G ATP1B2 MSR1 NEURL3 ANKRD54 SLC17A4 CRTC2 ITPRIPL1 PPP1R32 NET1 NPY1R TUB ESR1 NACA C1orf210 WDR49 ZNF790 ACE2 MCAM H2AC1 STRN3 SLC12A9 ITIH3 EDA2R CTAGE4 H3C11 CAMP TBC1D26 PDCD2L CABLES2 MYL1 ANAPC1 CLCA2 BTBD10 APTX DENND5B PLSCR5 KRT74 MUC20 FOXN4 OVOL2 PYGO2 PRKAG3 TMEM106A CENPN CDC20 STYX CBWD5 SELENOS C4orf33 LYZL6 DOK6 HIPK3 EPB41L3 PRSS3 BCL6 UTP14A ERICH3 DEFB121 PROS1 LIMS1 PAK1IP1 APOL1 PPP6R2 MANEAL SETBP1 MFAP3L AREG MSANTD1 MME CWH43 ELP3 GREM1 CHST2 DHRS7B PGLYRP4 BCL11B TNFRSF8 SOX15 MED16 ZNF444 S100A16 POLR3C SEMA4B S100A8 ALPK1 RSPH10B2 MTUS1 CHIT1 TADA1 RPS6KB2 TPST2 PYROXD1 PUS7L CHKA B3GLCT TMEM187 RNF138 WDPCP MAGI1 FAM120AOS SPAG4 SMG1 GAB2 IL13 LIX1 RAB4B MIEF1 SKIV2L RALB GNA13 SPIN1 IFRD1 TBX15 PSRC1 RAPGEF4 TSPAN14 SLC35G5 CCL2 GPM6A FUT8 SLC7A8 SHMT1 AMPD3 RHBDL1 FOXA3 RPL36AL EIF2AK3 TMEM62 HEBP2 NFAM1 ACADS TLR3 RBPJL ZNF260 MED30 NXT1 NRTN PSME4 FGF14 CDC7 HABP2 MED23 ATF7IP GALNT10 NANOS2 CER1 FAM131A APOF CD109 ANGPTL1 SRL PSTPIP2 RPS6KA2 C1orf109 ARHGEF40 GADD45B DHRS9 TEAD4 DNAH2 ZNF862 ZMYM3 OR8G5 NUP210 TGDS DMRTC1 THSD7B ELOC FAAP20 SLCO1C1 ATP6V0A4 CUL1 SIAH1 FGFRL1 SUCLG1 ADGRG1 MYO1E CXCR5 KRT2 TNFAIP1 DDX55 ANKRD65 ARHGDIB SEC23A CAPN8 KDM4A PSMA2 HACD1 C4orf45 DACH2 FH LYPD6B KIFC3 LGALS2 DCDC1 MYBL1 MTUS2 MUC22 ACYP1 COL3A1 ZNF610 RTN4 TK1 IL19 COL9A3 FIG4 ATP6V1F PRDM11 CTR9 UBR1 GGT1 ZNF140 UHRF1BP1 KCNK2 KDR PAM BTBD3 UBE2NL TBC1D4 SYT1 MRM2 ACRV1 MOXD1 SREK1IP1 SLC7A13 PCDHA10 ZNF701 GRAMD1B FLT1 NKAIN1 SLC25A2 FUT1 BDH1 RPLP0 NEIL2 RNF220 SHCBP1 NOL4 PRAMEF14 PPP1R12B IGF2BP2 TBX4 CBWD1 CMTM2 FOXO3B LRP6 LHCGR CBLIF SRP14 OCIAD2 ANKEF1 KRT72 KCTD4 LRRTM4 NFASC ARHGAP9 UNC119B CLCA4 ENTPD6 NAMPT SLC44A3 UBN1 BST2 UROC1 ACADSB CABP4 HTR1F HS3ST3B1 INPP4B FAAH2 SPPL2A GMPPB BMPR2 MZT2B HMGN2 TEP1 DMKN CES3 MAP2K5 ACP5 TBCE UBE2R2 MRM1 LILRB1 PDLIM1 ZNF229 GJD4 HOXA2 TEX44 TLE3 ALOX5AP OPN5 KLF8 H4C12 POSTN ID4 CBLL2 ANKH KCNJ4 C19orf18 ELP6 TACC1 MARCHF5 SYNDIG1L SAMHD1 PPP3R1 SPRY1 PAX6 AAMP NFIB SLC17A6 CDH16 UBE3B CYTL1 POU4F3 TNFSF11 CFAP298-TCP10L POLR2K BIVM TENT5D AKT2 PPFIA2 ANKFN1 MB STXBP6 HCCS MRPL46 FARP2 GTF2F1 ZFP36L1 SLC49A4 EXPH5 RBM42 RNF146 PCDH19 CIB3 TTC39A CBWD6 TSPAN10 RUSC1 PLA2G2D TRIM17 PURA NAXD ERBB2 FANCM PRICKLE4 HNRNPDL TCAF2 GPR152 RAB3GAP2 CFL2 ZNF286A ZNF302 ENY2 SRGAP2B FUT3 FIGNL1 ZNF226 ERP27 SELPLG ASIC2 ARMC9 MCOLN3 SSH1 MASP2 C1QTNF6 CAD TMEM190 DTYMK LEMD1 RPS12 ETHE1 RBM11 SCT CAGE1 TIMM23 SHISA4 DPH2 KCTD6 LRRC70 CAPN11 RPL18A ZNF71 OPRD1 WNT8A UBXN11 COPS8 CTPS1 EPHX1 GABRP SINHCAF ESS2 HBE1 ASB6 PPIF SPTB A1BG ICAM1 GOLGA2 RBM47 NMNAT3 PPP1R3E ABCC5 RAPGEF3 TRNT1 ABL1 CMAS PATZ1 RBM22 C14orf93 FAM156A OR9Q1 APLP2 CBWD3 ITIH2 TMEM164 TRIM38 BTBD11 MTFP1 CALN1 MMP26 GRID2 ELMO1 VWA7 FAM184A LTF ATP5PB ARSG HSP90AB1 PAAF1 PRSS23 DNAJB9 WNT2B OR13A1 SEMA6D PMAIP1 CABIN1 WBP2 MRGPRX3 C1D KIAA1191 TNIP2 GUSB SPNS3 SFTPB SLC30A6 GAS2L2 FBP1 RNASE11 EXTL2 PDE6B FBXO47 BEX1 SLC45A4 ELF2 PKMYT1 PKHD1L1 YTHDF2 GDF5 NKAPD1 H4C4 RNF133 LYPD5 FLRT2 ARMS2 COA7 PDE10A IFNLR1 TM9SF2 TMEM144 ERP44 SHOC2 METTL7A SH2D1B KRTAP9-2 NSFL1C FBXW7 NEUROD4 RHOJ ZNF180 ACP3 EIF4E3 INTS2 USP48 TMED10 FDCSP FAM9A MTCL1 ARFGAP3 KRT7 EIF2AK4 TMEM121 PDE11A TMEM17 TEX12 ABCA4 POTEM SYBU ABCA9 RNF214 TMEM87A GFAP OPRM1 SNAPC2 PDE4DIP RSPH10B LRRC1 SCG3 TRMT12 PEG3 CC2D2B CLVS1 ATF6 GPR85 CBWD2 TACR3 CA8 SLC35C1 EIF2AK1 FBLN7 SLC25A10 B4GALT3 NKAIN2 ACVR1B LHFPL5 - PSMD1 SLC41A3 BTNL2 TMEM128 SFTPC RNF186 CSN1S1 TDRP TMOD1 COQ8B ST6GALNAC2 RAB40C FGFBP2 MPP3 ARHGEF6 CDH23 ZNF525 FGF13 BUD13 UNC80 SPINK6 HHIP ETFBKMT ALPG DAGLA EFCC1 ADPRM ARHGAP6 SCAF1 CDKN1A LPL CHURC1-FNTB SERPINB10 RANGRF ANKRD53 ICE2 TTC6 PPP2R2B UNC5D PLEKHS1 CEP295NL SLC6A12 DDX23 FAM221A RARRES1 SCUBE2 VANGL1 CFAP95 RPL37A FLOT1 NREP P2RX4 CCDC71L SBSPON CTAGE8 CUL4A TBRG1 CFB TOR4A GPC3 NRXN3 KRTAP10-10 CACNG1 HELZ TRAK1 NPTN ADRB3 CLDN8 MS4A12 TTC14 DLC1 TSR1 ACP6 KCNQ3 PPP1R35 AKAP8 IFIH1 DENND2B EFNA3 SCRIB CHURC1 ARF3 PITX3 YAP1 KIF20A GRM4 HPX CFAP119 GPM6B RNF130 JAKMIP3 UCHL5 ELP1 FBXO38 IRF8 ORC6 TNFRSF6B GSTA5 HPS5 GFRA1 TMEM135 GDF1 MARCHF8 PREX1 UBR2 S100A7 TCF23 CXCR6 MAK IGSF6 TUBGCP6 VGF KRTAP9-4 TTPAL AGXT SLCO5A1 CERS2 ADPGK EIF3E RPL31 CTAGE1 DEDD2 S100A7A GFRAL ITGA7 CPT1C CLEC11A ASB7 ALB PTK6 HAMP CTSZ ZNF112 GPR68 ZNF385A SFXN4 UCP3 NR1I2 C6orf47 OST4 VAMP7 SLC13A3 CLIC2 PRICKLE1 C10orf82 TAS2R38 GBP5 LPIN1 SNRNP27 P2RY11 TRIM59 SLC25A38 PLP1 CNNM2 SERTM2 IFNA10 SUCNR1 TLR2 UHRF1 HTR3E KCNMB4 FZD4 SNAPC3 PRAMEF12 KRTAP9-9 PIGC BICRA TRMT1L CEBPB IL18R1 ZSCAN20 CCER1 INO80 CLEC10A GEM KRBA1 AMOT TESC ZSCAN22 CRAMP1 STAB2 ETV1 SLC35E2A IRF9 BACH2 NRG1 TCN2 UEVLD DMAC1 TGFBI ARHGAP40 MRPL30 RBM43 PGM3 GABARAPL2 TRIM25 HINT1 PRAMEF1 RNF212B MPG CKM ITPRIP RHOBTB1 CISH GPR39 RPS16 CSNK2A1 BABAM2 CHCHD4 ANKS3 NDC1 IL2RB CD3E DEPP1 MSANTD4 DTNA TMEM208 SLC17A8 CELF4 TMF1 ST8SIA6 LRRC14 METTL21C GIGYF1 STXBP3 SMARCAL1 ITIH4 TBC1D8B IFI27L2 TRIB2 COL6A6 LAPTM4B TNS2 ADD2 APOC1 SIGLECL1 ACSL4 REPIN1 C11orf54 NDRG3 KRTAP21-3 PEPD CYP1B1 GRIA2 VDAC2 ZNF17 PIP4P1 BTAF1 YPEL2 PPWD1 RHBDD1 COLQ LETMD1 HHATL PTCH2 PHF23 PSD3 GNL2 CCDC9 TTC30A PPP5D1P ZNF471 ADGRB1 KRT32 SGPP1 CSF2 TRIM50 GDF3 OSBPL5 CDHR2 PGK1 BICDL2 PALS1 CCL13 MISP3 |

**Table S3.**

Genes from summary statistics from the GWAS meta-analysis (147 genes that are annotated to the identified 16 genome-wide significant loci).

| Number of genes | Gene symbols |
| --- | --- |
| 147 | CREB3L1 IER2 EIF6 DCHS2 RDH13 AMBRA1 SCYL3 RPL7A PLEK PSMA3 CRY2 ACTR10 SLC44A2 GRK5 SELL FOLH1 ATP1B1 OR4A47 FGG CELF1 OGFOD2 GBGT1 UQCC1 CCDC181 ACP2 PEX16 OR4X1 TP53INP2 SELE SETD8 CYP4V2 KLKB1 F11 C1orf112 CHRM4 CDKN2D GP6 TOMM20L MYBPC3 ZFPM2 PACSIN3 C12orf65 C1orf198 DGKZ NUGGC SELP CACFD1 TACR2 STXBP5 PLRG1 FAM83C ATG4D C11orf49 VWF FAM180B MADD SURF4 RAPSN EDEM2 RALGDS ARFGAP2 ATG13 PITPNM2 AL132989.1 KRI1 SURF2 NCOA6 MMP24 XCL2 ACSS2 XCL1 RILPL2 DPT METTL18 TSPAN15 PLCG2 HIC1 SPI1 FNBP4 NME7 CDK2AP1 ARHGAP1 C11orf94 SURF1 TMEM8C NLRP2 GPR161 NUP160 ABCB9 FGA GGT7 C14orf37 REXO4 SLC2A6 ZNF408 SLC39A13 QTRT1 MDK DNM2 TBX19 KBTBD4 AC011475.1 SURF6 ARID4A NACC1 STX10 TIPRL C9orf96 ARL6IP4 SMG6 MPHOSPH9 AP1M2 F5 S1PR5 PTPMT1 PTPRJ LRP4 CKAP5 METTL11B BLZF1 C4BPB PSMC3 AC110771.1 PIGU TRPC4AP C4BPA AGBL2 SBNO1 MED22 LRAT NDUFS3 MTCH2 OBP2B MYH7B PROCR PHF21A SLC19A2 |

Genes were derived from the loci C1orf198, PLEK, OSMR-AS1, NUGGC/SCARA5, GRK5, MPHOSPH9, ARID4A, PLCG2, SMG6, EIF5A, STX10, SH2B3, SPSB1, RP11-747H7.3, RP4-737E23.2, and ERAP1.

**Table S4.**

DEGs from a meta-analysis of data from two independent transcriptomic studies of VTE. The meta-analysis reported a list of 125 upregulated genes and 48 downregulated genes in VTE across the three categories.

| Dataset | Number of genes | Gene symbols |
| --- | --- | --- |
| Upregulated genes | 125 | TCEB2, MRPS15, UBL5, S100A12, DPM3, RPS29, NANS, PRDX4, ZNF271, DICER1, CCDC53, ICT1, CLIC2, MYL6B, CD244, GADD45GIP1, DRG1, STX8, TMCC2, KRIT1, RTP4, STAG2, RPS21, RPS15A, CSTA, MCTS1, PHF20L1, CHD9, SP100, TIMM8B, TRIAP1, BFAR, MRPL13, LY96, NDUFA4, ZBTB44, OSBPL8, SKP1, PIK3R1, RORA, YIPF6, RBM41, CXorf21, USP33, UBFD1, UBE2G2, IFI27, KLF1, MYL4, PRDX2, AKR1C3, PDCD10, FKBP1B, SAP30, POLR2K, MTF2, AKAP7, NDUFB7, RASSF4, MRPL11, SLC14A1, NFAT5, ARL4A, KYNU, BAG5, LYRM2, MRPL15, CDKN2C, RPLP0, NDUFA7, MXI1, UCHL3, MXRA7, IFIT5, PBX1, METTL5, GAS7, RNF11, FIS1, CCNB1IP1, TAL1, RPL9, SPIN1, MRPL3, TSTA3, HMBS, GATAD1, TERF1, CD3G, PAK1IP1, UBXN4, LGALS3, FCGR1B, TES, CASP6, RPS24, RHOBTB3, MED13L, CCNT1, PSMA2, TGIF2, GYPB, SLPI, YOD1, XK, ITM2A, FECH, METTL9, SYNJ2, MCTP1, COX7B, SAR1B, PCDH9, SLA, MTHFD2, RAP2A, PLEK2, AHSP, CEP57, IFI44L, ALAS2, RPS23, PF4V1, ISG15, ISCA1 |
| Downregulated genes | 48 | MAZ, BRD1, HNRNPM, ZFP36L2, FOSL2, CR2, CALR, FUT7, SPTBN1, OGDH, YWHAE, MYC, HUWE1, CR1, TAPBP, ZMIZ1, SSH1, DYRK1A, TAF4, SIRPB1, PRKDC, POLDIP3, SGK1, MARK2, PRKACA, CYP4F3, MTMR3, GPR56, MAX, PPIF, NCOA1, SUPT6H, ALOX5, IDS, RAPGEF1, FOS, CHMP1B, BCOR, RAB11B, CD46, ILF3, ARHGEF11, ABCG1, NEDD9, JMJD1C, CXCR4, FPR2, HDC |

**Table S6.** Differentially expressed miRNA (DEmiRNAs) from a prospective population-based investigation. 12 DEmiRNAs were reported in this study. Genes predicted to be targeted by the identified DEmiRNAs 11.279 genes were identified.

| Dataset | Number of genes | Gene symbols |
| --- | --- | --- |
| hsa-miR-15b-5p | 1178 | ELMO2 TBC1D19 ANKS1A COBLL1 CHPT1 KANK1 EIF2B5 KCNJ2 MAP4 ZHX3 BTG2 CNTNAP1 VPS4A VAMP8 RNF38 PVRL1 KLHL2 SALL1 PTPRR WASL MACF1 ADAMTS18 CHRNE MSL1 MBNL2 PELI2 SIDT1 EPB41L4B ATXN7L1 ODZ2 CHORDC1 POU4F2 DDX11 BTLA CAPN3 SLC6A4 ANKRD13B SPTLC1 ZBTB34 HCN1 ARL2 CHD9 GNAI3 AATK PHLDA3 FGFR1 TOB2 MAP3K3 KLC4 EXOC5 COPS7A IRF2BP2 RNF41 IPO9 SRPK1 PI4KB TEAD1 SNX33 FRY AK3L1 RBM35A STRADB RAB8B FASN PCDHAC1 KIAA2022 EXT2 ABL2 CCBP2 LUZP1 BCO2 C19ORF6 CRYM ACSL3 BMPR1A RFWD2 CDC14A CNN1 USP6NL SNX16 ACACA RGS3 ZCCHC5 PHF20 EED CCR9 ARHGEF12 GLUD2 AP1S2 MTHFR MAN2A2 PLUNC SCOC DACH1 SFRS1 YOD1 SMAD3 TAF5 KPNA3 PDCD4 ANLN CEP350 FAM73A CHRNA5 C1QL3 FAM63B WHSC1 HTR2C GHR ARHGEF9 MKNK1 GALNT13 NKD1 DCBLD2 PRKAR2A RAD23B IHPK1 PPIL4 DICER1 CACNA1E PCDHA13 NCOR2 RTF1 PAFAH1B1 KATNAL1 UBE2I PCTK2 GGA3 PCDHA4 CCNE1 CAPZA2 sep.02 USP14 ZNF280C MINK1 PCMT1 VAMP1 RBM24 GRB7 SOCS6 IL1F5 FRS2 TAF7L MYO1C FIGF GRB2 ARNT BDNF C14ORF1 SEMA3A TRIM2 RNF217 BTRC TFCP2L1 UBR3 C18ORF34 OTUD4 GCC2 SORT1 YWHAQ CHEK1 KRTAP11-1 PPAP2A RTN3 PPM1A PCBP4 CPEB3 BCL2 SLC2A3 SLC25A35 PTHLH ATP13A3 ACOX1 SON MAFK ABHD2 TRAM1 KIF5B ATP1B4 CSDE1 ERLIN2 CDCA4 SFRS16 FAM133B C1ORF102 CLCN4 PRPS2 DHDDS MYEF2 DCLK1 SNF1LK2 USP42 TASP1 AGPAT3 STIM1 RICTOR LIPE IRS1 B4GALT1 TMEM161B SRP72 PPFIA3 MFN2 ESRRA CHD6 C11ORF68 CD40 DYNC1LI2 CLDN2 DEPDC5 MAPRE1 MOBKL2B TSC22D3 BACE2 RAB11FIP2 RCOR3 POF1B RASSF5 NRP2 ZNF609 SIRT4 KIAA0317 CACNB1 QKI C13ORF18 ACP2 FREQ TMEM33 AHCYL2 UNC84A SIPA1L2 BCL2L2 UBQLNL ABCF2 CDX2 MFAP5 ACTR1A IL6R HIRA AQP4 PIP5K3 PPP2R1B AEBP2 PRDM4 NBR1 PAPPA RAPGEFL1 INPP5J OCRL MARCH5 EVI5 NEBL RARB KIAA0174 KCNQ5 CNOT6 DLL1 SMYD5 UNG BCL9L RET LPPR2 HSPA4L ENAH C16ORF72 NR2C2 FAM54B POMZP3 RHOBTB2 FRYL MAPK9 FAM18B SLC12A2 PNPLA6 ESRRG OTX1 C17ORF59 HOXA3 FUBP1 EPB41L1 PTGFR LITAF STOX2 CCDC109A PHLPPL PCDHA2 FBXO33 ASH1L CAMSAP1 PLAGL1 RBM6 PCDHA11 PIM1 NMNAT2 GRLF1 GOLGA1 USP9X PIK3R3 SH2D2A ACVR2A PRRX1 PGM2L1 RBBP6 SCN1A FSTL4 ASPH WNT3A FLOT2 ZC3H6 GOT2 TFEC SCN3A CAPN6 NUAK1 DCX PITPNA CDC37L1 NRBP1 SLC25A22 CCDC6 EYA1 KSR1 SATB2 YWHAH CDK6 KIAA0664 ZFHX4 FHDC1 TSPYL2 MLL3 ABTB2 AP2A1 TUFT1 SNAP25 SMPD1 SVEP1 LIN28B RAPGEF1 PRKAB2 AFF4 RAF1 VTI1A DIXDC1 STC1 ZIC1 KLHDC8B LYST SPOCK3 FURIN ARL10 AMMECR1 WWC1 DLST EDAR ATP2B2 PLS1 ZYX ANK2 TLK1 MAPRE3 RIMS3 COPS2 SMAD7 C14ORF129 TUBA1A SLC1A2 SGK1 GPATCH8 LMOD1 MOBKL3 HIGD1A SNRPC LTB CPD SUMO3 BZW1 FGFR2 RRAGA HOXA10 BAI1 KIF21A DNAJB4 PRKAR1A DOLPP1 FAM120A ZNF518B EPHA7 LMAN2L PEX13 MAP7 SCN4B PHF19 EPB49 CHUK RAB6B AKAP2 UBXN10 PPP2R5C PANK1 TFAP2D CD2AP LRP1B XPO4 TMUB2 AQP11 EIF2B2 WNT5A SH3BGRL2 KALRN WIPI2 USP25 EMR2 IFNAR1 ITPR1 TBC1D20 KIF5A EIF5A2 NF1 SLC44A1 FCRL2 EIF5A WBP11 SPRED1 PLRG1 MAP1A MYO5A BCL7A ZFYVE20 SPEN GALC CDC42EP2 CHP ZNF362 GABRE C14ORF43 UBE2J1 HAS2 TGIF2 CDC42 VPS33B FLJ37543 CRIM1 DHTKD1 RUNX1T1 RIC8A CCND1 SFRS2IP NOTCH2 KIAA0652 PDCD6IP C9ORF25 FAM122A ATXN2 RS1 GABARAPL1 IL15 PLEKHA1 ODF2L STK33 ARHGAP12 HPCAL4 KCNK1 ZCCHC3 FKBP5 PCDHAC2 NFAT5 TMEM55B LRRC15 WIF1 TPPP3 DVL1 GSTT2 PPM2C PLCXD3 IRS2 RIMKLB USP2 GALNT7 ABCG4 GBA KCNAB1 CUL3 DMTF1 HMBOX1 GRM1 NFATC3 FAM122B ITK ZKSCAN1 PCDHA12 LRIG1 SELI CCNJL ERC2 BSDC1 ARFGAP2 RNF43 MAP2K4 ACSL5 PTPN4 LYPLA2 ADRB2 TRIM9 LHFPL4 WSB1 TBP G0S2 FCHSD2 VEGFA SLC4A7 ARPP-19 ZNRF2 ELL FKBP1B CREBZF sep.05 IL3 ZBTB41 PPM1E BAT2D1 CDS2 VAV2 ITGA2 PTCH1 DOCK4 VAT1 THSD3 ATG9A SHANK3 TYRO3 POU3F2 ARRDC4 C10ORF46 IL1RAPL1 E2F7 RNF138 UBAP1 HOXD1 SLITRK6 FAM91A1 RAB4B MARCH9 KIF1B EGR3 SEH1L FOXO1 KIF5C TSPAN5 PDK4 SNTB2 WDR82 MAPK1IP1L KPNA1 CCDC28A TRIP10 PDIK1L NEK10 PIAS1 WWP1 FSD1 LAMC1 CCND3 C9ORF150 GCLC GABPA PBX3 PVRL2 CAMK2G KIF3B EFCAB4A FAM123B KARS MTMR9 DLL4 SETD3 CASKIN1 SEMA6A CHAC1 TMEM154 EPHA1 HMGA2 WEE1 UBE4A SMURF2 TMEM100 CCDC47 RNF165 CREBL2 NUP210 CREB5 TNRC6B RANBP3 WIBG DYRK1B EIF2C1 ZNF831 CC2D1B RNF213 SIAH1 KIF23 FAM60A COPS7B EIF4B WDR47 AP1GBP1 XPR1 ITGA10 FERMT2 CLOCK SLC35A4 NUP35 CLDN12 IRF4 C18ORF1 ARHGAP20 MYBL1 CCDC19 C9ORF5 CNTN3 IHH PPT2 CDC27 DCTN4 IVNS1ABP C1ORF21 CD163 AP1S3 KDR LMO7 PAM WDTC1 PPP1R2 GLUD1 GIT1 OGT FBXO21 LSM11 KIAA0831 GRB10 SFRS2 KPNA4 PHIP RBM12 SLC11A2 D4S234E FEM1C TGIF2LY CARM1 EIF4E ANKRD57 LATS2 DEDD C1ORF9 SMAD2 GADD45G PPP1R12B HTR4 VPS37C BTN1A1 LRP6 EZH1 PPARA NRN1 PLXNA2 SLC2A14 PCDHA5 PTPRD PLDN ATP6V1B2 CDC14B SLC22A17 ARHGAP18 KIAA0247 PPP3CB USP15 DNAJA2 SMURF1 LRRC55 HECTD1 HNRPDL BACE1 STX1A STX17 USP6 CTNNBIP1 IRAK2 CBX4 METTL9 NTRK2 HCG_18385 sep.11 MSH5 USP31 SLC36A1 CNIH2 CPEB2 BPTF PAK7 SR140 CHD7 ISLR LRRK1 CD80 SENP2 MLLT6 SNRPA1 PCGF5 SOX5 PRKG1 SMARCD2 TACC1 UBL3 CAPRIN1 EIF3A KIAA0182 ZNF238 FGF7 RASL12 IKBKB LPHN1 SESN1 AXIN2 CARD10 ZBTB33 MGA MYLK RASGEF1B TFAP4 IRF2BP1 CLCN5 BCR SYT3 DZIP1 FLT3 CHD2 ACSBG1 MTMR4 SLC9A6 DCUN1D1 DAB2IP FAM134A PURA DCUN1D4 TBPL1 NIN DDX3Y MYT1L PLA2G15 GRIN1 CALU FAM116A IQGAP1 PLAG1 EDA KCNA3 SYS1 CMPK1 SCN2A CD28 PAFAH2 WNT7A C12ORF30 ZNRF3 ENSA ZNF449 BAZ2A NISCH RNF144B ZBTB9 BLCAP MEOX2 TTL C20ORF46 FGF2 KCNG3 MED1 AMMECR1L PPP2R1A GLIS3 AP3M1 DIAPH1 ATXN7L3 KRTAP4-4 PHACTR2 TBL1XR1 C20ORF39 ST8SIA3 KCTD11 BFAR GABRP CX3CL1 ASB6 IGF1R PPIF MED13 FAM179B EFNB2 RECK SIX4 PID1 NLGN1 ACSL1 ABCC5 C6ORF204 NAV1 SLC20A2 JARID2 KIAA1549 KLHL18 SHROOM3 ZNF367 MKL2 FAM130A1 SLC9A1 TMCC1 NFE2L1 ZNF423 CA2 LRRC7 LRP2 UNC13A SEMA6D IKZF4 TARBP2 SYNE1 RSBN1 TMEM85 APLN SNRK DMRT2 GPN1 SNCG UBE4B CNTNAP2 KIF1C PPAP2B SUZ12 CDK5R1 KIAA1305 SIDT2 WNK3 SEMA3D ZBTB10 C1QB WDR22 SPTBN2 SLC41A2 RNF111 WISP1 EGLN2 PTH KIAA0895 NPR2 TMEM189-UBE2V1 ARHGAP5 POLE4 CSPG5 SPRY3 DYNC1I1 EN2 CAB39 MAP3K4 CYLD PSKH1 SHOC2 FBXW7 NCKIPSD NTNG1 CLCN3 CASR MAP3K7IP3 PCDHA3 SSR1 TFAP2A MGAT4A ZER1 ARL3 PPP2CA SRPX PHF15 CTDSPL SALL4 PCDH17 CADM1 LRRN3 TSPAN9 CBARA1 SGCD TMEM87A KLHDC10 GFAP ZDHHC14 KCNC4 CYP26B1 SEC62 CXCL10 PCDH9 ZNF532 ZC3H11A UBTD2 ACVR2B TCF3 IYD IL10RA ISOC1 ADAMTS3 STK19 SYDE1 GRM7 RANBP10 RARG GLS CECR6 C3ORF23 DSEL PRPF38A SHROOM4 ALDH1A3 PEX5 C2ORF42 MNT PPP6C OTUB1 INSR CRTC3 ZCCHC2 LRRFIP2 PURB SYPL1 KBTBD2 BAG5 FNTA SLC39A10 TGFBR3 PDIA6 CDC23 SLITRK1 ZMYM2 UMOD SRPR AMOTL1 MTSS1 MIPOL1 RAB11FIP5 SPSB4 KCTD8 SYNJ1 MMD LAMP2 ANKRD53 PCDHA1 PTPRM AGAP3 CUL2 MARCH4 HEY2 GORASP2 C5ORF24 CAMKV ARHGDIA CDV3 NUDCD3 PPP1R11 N4BP1 CCND2 LCP1 PIK3R1 KIAA0226 PTPN3 PCDHA8 HOXC11 TMEM55A CIAPIN1 TRAK1 HELZ C8ORF58 RNF125 STXBP1 FRMPD1 WDR68 KIAA1804 CD47 PRKACA C1ORF131 SCN8A ZFHX3 SPRY4 HEMK1 DPY19L4 RAP2C KCNC2 SOCS5 YAP1 KLC2 MLL2 PSMD7 CDC42SE2 GPM6B FNDC3B MTCP1 MASP1 GPR63 ZHX1 MAP2K1 ZNF654 CD164 TMEM135 MED26 UBFD1 MAK IGF2R BMX RNF8 UBE2V1 TLL1 HOXC8 PPP1R14C ADSS ANKRD34A NARG1 SLC24A3 MKX ATP2B3 POLR3F HDGF RAB10 TLE4 SOBP ELL2 SH3GL2 IL28RA COL24A1 RSPO3 ANO3 ZNF326 TXN2 TAF15 JPH1 PFKFB4 DLK1 SPAG7 C14ORF4 ZNF436 HTR2A C11ORF42 ARMC5 VAMP7 KIAA0427 SLC13A3 ETNK1 ZBTB5 PSME3 CNNM2 ZSWIM3 FAM70A GALNT1 GNA12 PLEKHA5 SEC24A RELN SPRYD3 C9ORF100 AKAP11 XIRP2 SUPT7L SERBP1 AMOT YTHDC1 STAG3L4 ETV1 ADAMTS5 STK38 ONECUT2 BACH2 NF2 NRG1 ADAMTSL3 TBC1D9 VTA1 ESPN LPHN2 NXPH1 FAM81A MTMR3 DDX3X KCNN4 CRKL KDSR SNF1LK DYNLT3 NDP OMG SLC4A4 UBE2Q1 AGTR2 FAM119B E2F3 ZKSCAN2 ZBTB46 KLHL3 HSPG2 CD3E GOLGA4 HNRNPA1 C20ORF29 AKT3 TRIM36 MTMR11 GIGYF1 STXBP3 FKBP1A ZC3H12B GOLT1B FOSL1 TBC1D8B MIB1 TTC1 BAIAP2 EPHB4 SYT4 LRIG2 PISD ZAK ACSL4 MBD1 WAPAL ARL8B SFRS11 GRAMD3 PPM1D FBXO10 ARMCX2 PAK2 GLS2 APP COL12A1 ELMOD1 SAV1 EIF2C4 ATF7IP2 PDLIM5 RPS6KA3 MAP3K9 BTAF1 SMAD5 PCDHA6 FCHSD1 SCARF1 RYBP USP3 MYADM ACTR2 CNOT6L ZNF622 PDE3B KCTD1 RPS6KB1 CBFA2T3 DRD1 MYB PAX2 TMEM20 SLC5A3 SNRNP48 FZD10 NR4A3 CMTM6 SUPT16H BCL11B UBE2B CCNT2 ZC3H12C BHLHB3 PCDHA7 GPR124 ZBTB39 NUP50 CDC25A |
| hsa-miR-106a-5p | 1072 | PTGER3 MMP2 ERBB4 KCNMA1 CCDC25 KIF26B APBB2 HECA COX7A2L PFKP RNF38 KLHL2 SALL1 WASL CEP70 CXCL14 WWC2 RGMB MSL1 PTEN AFF1 CYCS EPB41L4B GUCY1A3 SENP1 DDX5 FNBP1L KBTBD8 HMGB3 AP1G1 C7ORF60 SMARCC1 KLF11 CHD9 PAPOLA ZFP161 GDA SLC16A14 JMJD3 IRF1 DPYSL2 ARL1 TET3 MAP3K3 SSH2 IRF2BP2 SOX4 NR4A2 PTPDC1 CEP135 SORL1 CHD1 LYRM2 RAB8B GJA1 RPS6KA5 ATG2B LAPTM4A DIP2A KIAA2022 PCDHAC1 ARL4A KIAA1522 TAOK3 HOOK3 CRY2 RUNX3 GPR137B MECP2 LUZP1 ZNF202 EFNB1 MAP3K5 ARHGEF7 RAB7L1 PKD2 CNN1 PALLD ROCK2 SNX16 PPP1R15B ZNF704 TRPM6 INTS6 SQSTM1 C1ORF63 TMEM127 KPNA2 ZNF197 GABPB2 YOD1 CTDSPL2 KPNA3 KCNA1 RHOC PPP3CA PTGDR PRR16 C20ORF103 DCBLD2 IHPK1 RAD23B YTHDF3 DICER1 PCDHA13 ZNF148 KIAA2018 CNOT4 PAFAH1B1 NEFH PCDHA4 PGBD5 FYCO1 RAPH1 LPGAT1 SLC16A12 CEP170 MINK1 ATP11A TRPV6 HECTD2 SOCS6 FRS2 ADAT2 REEP3 BTN3A1 OPHN1 TRIM3 ABCA1 ACBD5 OTUD4 GCC2 PLXNA1 SORT1 TIPARP CCDC137 HS2ST1 GMCL1 YME1L1 CPEB3 ULK1 BCL2 CAMTA1 MAP3K14 PTHLH SLC40A1 CADM2 ABHD2 PTP4A2 USP24 TWF1 RACGAP1 SC4MOL CSDE1 PXDN TBC1D17 DUSP2 ERBB2IP SCML2 MAGI3 CEP120 NAP5 SPTBN1 CAMK2D MARCH8 CNGB3 MFN2 SGK269 RAB5B BAMBI RNF145 FGD4 DYNC1LI2 FAT2 E2F1 NPAT CRIPT UPF3A MAPRE1 RGL1 LIF CASP8 SDC2 BNIP2 QKI C14ORF145 EPHA5 FAM130A2 BCL2L2 ARL4C CCNG2 SCD5 LASS6 HABP4 AQP4 PIP5K3 C7ORF41 SPG20 MYNN PPP2R1B TANC1 ORMDL3 DCUN1D3 CORO2B RAPGEFL1 EPHA4 OCRL HSPA8 RARB CNOT6 TP53INP2 PRRG1 HP1BP3 HLF TUSC2 MASTL RUNX1 SUV420H1 GMFB C16ORF72 KIAA0494 MID1 NR2C2 PHF1 CENPO CROT MAPK9 TRIM37 GRHL2 UBE2W TIAM1 RPP14 AHNAK FAM175A FOXJ2 CNR1 PHLPPL FAM126B PCDHA2 ASH1L ZNF711 CC2D1A ELOVL7 SERTAD2 PCDHA11 GOLGA1 GRLF1 USP9X IQSEC2 SH3PXD2A MTF1 RNF2 PGM2L1 PRRX1 YWHAZ ANKRD52 SCN1A PFN2 ZC3H6 TMEM25 SV2B SCN3A PLSCR4 CDC37L1 UBR5 NRBP1 NCOA3 CHAF1A EYA1 CASC4 ZFPM2 SLC35F1 GPR158 LIMA1 ZNF236 MLL3 CTSS TNKS2 AFF4 MAP3K8 DIXDC1 STC1 TIMM17A LYST FURIN SLC16A9 EGR2 C12ORF36 NEDD4L PLEKHA3 ATP2B2 FRMD6 PLS1 KLRAQ1 ANK2 HARS MAPRE3 LDLR SMAD7 VLDLR FAM177A1 ELAVL2 PPP1R3B SLC1A2 RIMBP2 FAM160B1 DNAJC16 TANC2 ADRA1B POLR3G MLL4 KLHL28 IL8 ARHGEF3 UNK NTN4 B4GALT6 ATG5 ZRANB1 SERP1 EPHA7 EIF2S1 EGLN1 MAP7 MAN1C1 KIAA1147 SGTB ZNF264 NKIRAS1 STAT3 LRP1B HBP1 ORAI1 TTC39C FTSJD1 ADAM9 XRRA1 FAM19A1 XIAP TMUB2 GALNTL2 DPYSL5 GNS EMR2 MKLN1 RAB11FIP4 EIF5A2 TBC1D20 OSM SUMF1 KIF5A FGF4 C17ORF39 ZFYVE20 SULT2A1 SCRT2 NAV2 RABGAP1 ZNF362 GABRE OXR1 C14ORF43 UBE2J1 REPS2 HAS2 ZNF217 VEZT PRDM6 MIDN CNOT7 LHX8 COL19A1 CRIM1 CCND1 CTSK HNF4G SLC9A2 MSR1 AP2B1 SLC30A7 ZBTB7A BMP2 UBE2Q2 WDR42A ESR1 KLF12 ARHGAP12 STK33 ARHGEF18 PLEKHO2 MEX3D FKBP5 PKNOX1 PFKFB3 PCDHAC2 NFAT5 SCAMP5 EREG SIN3A OSTM1 AGFG2 CDCA7 PLCXD3 NAPEPLD CRYBG3 ABCG4 BTBD10 ZBTB47 CUL3 DMTF1 DENND5B RND3 IL1RAP MAT2B RBL2 CASP7 PCDHA12 ARHGAP26 KCNJ10 YPEL1 LRIG1 ITGB8 FBXL5 USP53 PTPRT PRKACB LACE1 E2F5 PTPN4 RASGEF1A STYX ANUBL1 HIPK3 SOCS4 HN1 SLC4A7 VEGFA SGMS1 MFAP3L SLC7A11 DERL2 ZBTB41 NRSN1 GOLSYN DOCK4 SLC17A7 CAMK2N1 WDR26 NR4A3 FRMD4A BCL11B CCNT2 UBE2B UNKL ZC3H12C BHLHB3 GOSR1 JAZF1 SLC35F3 PCDHA7 JOSD1 SEMA4B POU3F2 WASF3 ACPL2 KLF9 C10ORF46 TXLNA RUNDC1 LIN7B YES1 UBAP1 POLQ MAP3K1 LAMA3 ABT1 ANKRD9 PBK SLC11A1 ATL3 SPATS2 PCYT1B LASP1 ZBTB4 EGR3 RAPGEF4 HEG1 ZBTB6 MCL1 SNTB2 WDR82 PRR11 TXNIP TRIP10 ST6GALNAC3 LAMC1 M6PR NCOA6 STAT1 PBX3 GRAMD1A USP37 KIF3B CDC40 LPHN3 CREM METAP1 HMGA2 TMEM154 ETF1 MMP24 WEE1 SSFA2 TOPORS TMEM100 RPS6KA2 FXR1 FAM84B CREB5 TNRC6B ZFYVE9 EIF2C1 WFS1 TRIP11 GRIN3A LYPD6 CUL1 FAM60A KIF23 B3GALT2 SMOC2 TNFAIP1 PKN2 ITGA4 CLOCK DSG4 NUP35 MCF2L NPAS2 STK11IP C9ORF5 CNTN3 VASH2 PHACTR4 ARHGEF11 PAM TCF7L1 FBXO21 UBASH3B KIAA0831 SFRS2 KIAA0240 PCDHA10 TBL1X MYLIP FEM1C EPB41 RB1CC1 SHCBP1 CLEC4D ANKRD57 DEDD C1ORF9 NOL4 ZC3H7B PPP1R12B ZDHHC1 MMP3 HTR4 FOXJ3 YPEL4 BTN1A1 SACS AFF2 EZH1 PPARA SHE MGLL PCDHA5 SASH1 IL17RD PTPRD SLC22A23 LDLRAP1 ZNF597 PLCH1 NAMPT LRRC55 ATP1A2 BCL2L11 HDAC4 USP6 FAM102A BMPR2 INHBA ZFP91 SAPS2 NTRK2 NEUROD2 MIER2 KIAA0513 GPR137C USP31 PLCB1 MAPK1 ATAD2 PAK7 SR140 WDFY3 PHF6 TNRC6A WIPF2 TACC1 ADHFE1 OCLN PPP3R1 USP46 PSD FAM117A FAM57A NFIB MED17 DIP2B CAPRIN1 SPTY2D1 TNFSF11 ZNF238 NBEA DAZAP2 RASL12 KREMEN1 ENPP5 CYB5B SP1 ODZ1 ARHGAP1 ZBTB33 MXI1 CIC ZDHHC9 SH3BP5 MAP3K2 ANKRD13C CEP97 NKX3-1 SNX9 FAM134A FOXA1 PURA PCNP SIRPA ERBB3 CSGALNACT1 C14ORF28 ABCB9 NIN SRPK2 SMAD1 MYT1L CFL2 OSR1 STAG1 FAM116A ANKFY1 PLAG1 ZHX2 CMPK1 SCN2A PAFAH2 CD28 TP53INP1 TSHZ3 SLITRK3 FOXL2 TBC1D10C OPCML ZBTB9 ITPKB SLC25A27 FGF2 SLAIN2 SKI PLEKHB2 TET2 GLIS3 PLEKHM1 TNKS1BP1 FNDC3A CA10 TMEM50B RRM2 AKAP9 RASSF2 ARAP2 PPP2R2A PRR15 CAPRIN2 ANKRD50 EFNB2 F2R GNPDA2 TAL1 TSG101 STRBP ABCC5 RBBP7 LHX6 IQSEC1 MCHR2 PRDM10 DNAJC27 TNFRSF21 PLAC1 ZNF367 FBXO41 PLAGL2 MKL2 TBX3 ALX4 CORIN MBTD1 TMCC1 RASL11B FOXF1 USP33 SART1 UXS1 MAP3K12 TGOLN2 DNAJB9 SLC24A4 PAFAH1B2 FGD1 IKZF4 MYCN SYNE1 RSBN1 ANKRD29 SMOC1 KIAA1191 GTDC1 DNM2 GPR6 RABEP1 TNFRSF10B NEUROG2 CCL1 RASD1 HIF1A KIAA0922 SLC2A4 PKMYT1 WNK3 LDLRAD3 MAP2 PTH CRAMP1L SEMA4G RAB22A NPLOC4 ZFYVE26 RB1 VSX1 ZNF280B ADAR BAHD1 LYSMD3 FAM40B PIP4K2C SHOC2 FGD5 C19ORF2 CANT1 PCDHA3 XRN1 FBXW11 SLAMF7 CHRM2 SLC25A36 RAB11FIP1 CSNK1G1 KIF13A C7ORF43 CTDSPL CLIP4 PTPRO TSPAN9 SLITRK2 E2F2 THRA MLL5 SGCD CAMTA2 ARID4A FAM123A ST6GALNAC6 CYP26B1 PKIA APCDD1 C11ORF30 RAB12 ANKRD12 RAB30 ZNF143 GNB5 GBF1 ZNF295 ITFG1 SNIP IYD COL4A1 LMO3 NEUROG1 TMEM64 MED12L GRM7 C9ORF82 BRMS1L TMEM123 DDHD1 RAPGEF2 BICD2 PPP6C KIAA1024 SLC29A2 SHANK2 SLC41A1 STK11 RAPGEF5 TGFBR2 PURB CHIC1 SRGAP3 ACAP2 SULF1 AKAP13 MARK4 CDC23 MTERFD2 PTGFRN ZNF512B CIT NBL1 RAB11FIP5 DYRK1A CDKN1A SAPS3 RPS6KA1 PCDHA1 CRK PDS5A ATXN1 CHD5 MARCH4 TBC1D15 JAK1 SETD7 FBXL11 BTBD7 ZNF800 ITCH VANGL1 PDZD11 PPP1R1C P2RX4 BNIP3L CELSR2 CCND2 TMEM133 ADIPOR2 C1ORF107 PIK3R1 KIAA0226 PTPN3 PCDHA8 PDGFRA PTPRJ SCAMP2 TET1 TCTEX1D1 FOXP1 KLHL20 DLC1 CD47 SNRPD3 C1ORF173 PPP1R1A SPRY4 RGMA RORC RAP2C MXD1 RSRC2 WDR37 EGLN3 CHURC1 SSX2IP ST8SIA2 L3MBTL3 RRAGD FNDC3B KIAA1128 DLGAP2 TAPT1 FASTK SS18L1 HPS5 TIMP2 ZBTB44 FAM46C JUB F2RL3 NEK9 PREX1 NHLH1 GABBR2 ASF1A SP8 UBFD1 FCRLA SEMA5A CABLES1 ZNF791 CALD1 FLJ36031 SLC24A3 TCF7 TGFB1I1 UBE3A KIAA1462 RAB10 TLE4 C2ORF69 ARID4B SOBP ZNF2 SFMBT1 RP5-1000E10.4 FAM117B F3 EIF4G2 ZNF37A ROD1 ZNF385A UCP3 ALDH3B1 FEZ2 OBFC2A RAB18 UBE3C ZNFX1 KLF10 CD69 MKRN1 KIAA1598 ZBTB5 MORF4L1 SLC30A3 ELK3 PEX5L MGEA5 BCAT1 STX6 LIMK1 SCN2B NKX3-2 MACROD2 ZSCAN20 GATAD2B VPS13C AKAP11 TRPS1 INO80 CASP2 NAT12 BNC2 THAP6 AMOT NANOS1 ATG16L1 ETV1 NAGK USP28 TTC9 STK38 ADAMTS5 IRF9 CYBRD1 RGS7BP TBC1D9 UEVLD GAB1 MYO1D PPP2R3A CEP57 MTMR3 MAPK4 SNF1LK SESN2 SFXN5 RUNDC3A EPB41L5 ARHGEF10 TMEM168 MAP3K11 SLC4A4 FJX1 ADAMTSL1 GIT2 E2F3 RNF128 PHTF2 WAC FAM155B JRKL EEA1 AKT3 GIGYF1 FCHO2 ANO6 ZNF697 NPAS3 RUFY2 TBC1D8B FAM13A1 FAM45A NDEL1 EPHB4 SUSD1 STK17B PAPOLB MAL2 PARD6B ZAK ACSL4 TRIM8 RNF6 SAR1B BTG3 RBL1 KAT2B APP ATP2B1 VHL SMAD6 PDLIM5 RPS6KA3 MAP3K9 BRWD1 UBXN2A SMAD5 IGF2BP1 WASF2 PCDHA6 YPEL2 CAMK2N2 SNX21 RYBP PNKD USP3 FRMD4B UBC TTC30A ZADH2 CCDC88A CNOT6L DUSP8 PDE3B OSBPL5 TXNDC10 DRD1 KLF3 |
| hsa-miR-197-3p | 410 | DPP8 COL4A6 AGO1 PHAX UBA2 NKPD1 CMBL ATF6B GALT CKS1B DGCR8 CNBP SLC4A1 PDF RRAD IYD PMPCA GAPVD1 HPN RAVER2 MTHFD1 ACVR1B G3BP1 ARGFX TRPV2 HNRNPH1 RBM41 SRCAP IER3 CENPJ TAF4B RRP36 RBM4B MRM2 GNA14 CYCS COL22A1 BMF MORC3 MRPS10 C2orf68 UMPS GDE1 NEMP2 UTP4 ZNF417 ORAI2 INTS4 KLC1 PFAS PURB MAP4K2 HNRNPD SS18 POLR3A EIF4E TFDP2 TMX4 SOD2 METTL4 KIR3DX1 H2AZ1 HEATR5A MED24 MSRB3 C6orf132 RAD51 FOXJ3 RPL13A IER3IP1 ZNF208 PALD1 DNLZ ZYG11B PNPLA3 ZNF384 SHE TSPAN3 AK3 IPO9 HBS1L CSNK1E DBT SNX1 RFX1 HOXA13 RPL7A SAMD8 HMGN1 HSPA1B GORASP2 UQCRB NUS1 NAA50 ABCC3 FASN OVCH2 CHSY1 PRIM1 ATP1A2 TANGO2 GSTO1 DDX19B RGS16 IARS2 CLN8 RPL22 PEX13 SCD QSOX1 FOXN3 NME2 LZIC CGAS CPSF1 ANTXR1 HSPA6 SH3BP2 C1orf115 DNASE2 ZMIZ2 PLIN3 MAPK1 GOLGB1 EIF4H HNRNPA2B1 MYH10 DLC1 ICA1L PHF20 RUVBL1 NUBPL LARS1 LYPLA1 C19orf47 SUDS3 HARBI1 ZDHHC22 RSRC2 ZNF793 YOD1 IFNAR1 PRKX ZNF274 PTBP3 PDCD4 CCDC90B YIPF4 SPIDR IGF2-AS EHD2 LRP4 SSC5D ATP5MC1 USO1 ATXN3 KIAA1958 ABCA6 BORCS7 KREMEN1 NOA1 MICA DCBLD2 NEK4 PRKAR2A MAML1 RAD23B SOX11 NRL SKP1 GPR156 CEP152 CACNG8 ALX1 SOD1 LAIR1 GSTK1 IL18 TIAL1 PRKD2 PCYT1A ZNF101 BCR MORN2 TTPAL RBM27 TRAPPC1 MYO6 AKAP6 CASP10 GSTM5 RBM15B GAN ATP11A TULP4 RAB3B MEGF9 E2F4 MCM7 STX12 LMOD3 RAB28 ATAD3C ORMDL2 HSPD1 CNNM3 TMEM192 CLUAP1 GGT7 ZNF302 CBX5 RNFT2 GPN2 RBM4 PPDPF GMPS SLC16A5 JTB CPT1A ALMS1 DMXL1 CHEK1 PTPN14 CD82 LRRC58 ARSL SPPL3 TRIM72 HNRNPR TTC8 ZNF175 PPIA VDAC1 ZNF551 CCNG1 PHC3 PTPRT NKD2 SEMA6B GNE ZNF677 ZNF354A FOXO3 KLF10 MYH9 MKRN1 CTCFL HACD4 PHPT1 HIPK3 PDZRN3 LRIT3 FUS LIPG RNF222 ZNF324B GDF7 SLC35E1 CYB561A3 MANEAL WNK1 ADNP2 ZNF784 IPP DENND6A ABHD18 TMSB4X STMN3 SEC24A TADA2A NSUN5 ACVR1 SERPINA3 SPATA2 GTF2H3 LDHB MIR197 ZFAND4 FGD4 ATP2A2 ART4 PLCB3 JARID2 ZNF584 SHISA9 MED16 CCNA2 TRMO STRN UEVLD CRISPLD2 CHIC2 BACE2 CPNE6 CLPP MIOX TYRO3 ACTG1 ALG1 ZNF318 FGB TAOK1 RAB31 CLEC17A MT-ND6 ACTL9 WDPCP OTUD6A DIPK2B PMAIP1 THEMIS2 ALDOA RDH11 TRMT9B NCL TSPYL1 PLEKHG2 SNU13 KPNA5 WDR6 KIF1B RPL14 UBN2 UTP6 LRPAP1 HNRNPA1 GMEB1 TMF1 STEAP2 PBX2 ORMDL3 HNF4A TSPAN14 IDH3B IL1R1 ZNF813 HSPA4 CYP2W1 ZNF616 FBXL13 CLIC1 CHD4 TERT ZNF70 MRPS16 GLA CEP19 PDIK1L TCOF1 NUDT3 VPS41 NGDN PALS2 CES1 PIPOX TSEN34 ATP8B1 SYNGR1 RSBN1L PGPEP1 TUSC2 EIF5AL1 IGFBP5 CDKL1 TRAP1 AGTPBP1 DPYSL3 CDC73 MINDY2 ZBTB40 RAN ADAR FKBP4 GPR26 SPRING1 EEF1A1 MMP23A TENT5B KIAA0586 WASF2 CDK10 TMEM154 CYLD MLN FBXW7 ZMYM1 SETD1B FOXJ2 MYOZ3 ISYNA1 AGR2 SPART PPP2CA MSI1 TRAT1 RANGAP1 ZFR2 TNRC6B SNX22 ATP1A1 FRMPD3 SMCR8 DYRK2 FPR1 ADIPOR1 ZNF708 CHTOP ZNF747 DPH1 RXRB |
| hsa-miR-652-3p | 170 | ACTN4 SUPT6H NFAT5 AGO1 MXRA7 PTPLA TMED5 CNOT3 HIST1H3B GABRB3 YIPF6 MCTS1 HIST2H4B DGCR8 ACVR2B KIF1A ISOC1 C1orf85 LRP8 RNF152 IRS4 RPL26 UHRF1BP1 KCNK2 RAC1 CD46 EML4 MRPL36 CYTB KCTD10 YWHAH ZFAND5 PHF12 COX2 MORF4L2 RPL35A ATP5B ATP11C SLC12A6 IARS RPL18A NUP98 DDX39A ATP11B CCDC74B HNRNPAB HIST1H1C ELOVL1 C11orf48 ARCN1 AP1G1 CDC42EP1 SMAD2 PNPT1 SEC13 TCP1 ND1 HIST1H2BH VCPIP1 NXN USP10 IGF2BP2 FGFR1 SERBP1 MRAS AGO2 GTF3C5 CEBPG EIF4ENIF1 ONECUT2 SRPK1 SLC7A1 CHST6 AGAP3 HIST1H2BB POM121 STK4 HSPA1B ZNF431 ACTG1 BTF3 CSNK1A1 TLR8 ZNF567 MSI2 TAZ NFE2L1 H3F3B QKI SLC31A1 RPL27 RPS6 FAM120AOS ANKRD9 RPS16 CSNK2A1 SMAP2 RPL32 NOM1 CDKN2AIP COX20 PCSK7 ZBTB4 GRK6 DTNA NPTN VPS37B G6PC3 RPL29 GRPEL1 CNN3 HIST1H2BJ LRRC14 ATP5IF1 HIST2H3A ADM2 CSAG1 DLC1 RPL21 SRM MTA3 KMT2C VPS41 IMMT RPS19 CTC1 ISL1 TNRC6A IPO13 RPS29 ZBTB8A UGT2B4 MASTL GDPD5 PRKAA1 CAPZB CARS SAR1B YBX3 MGST1 SF3A1 SLIT1 ND4 SERF1B ATF7IP CBS SAT1 HOXA9 EEF1A1 PPIL4 ZBTB44 AGO3 TUBA1B UBE2I CACNG8 KDM2B SNX5 BSG CCDC142 RPL4 HIST1H2BD sep.02 HIST1H1E POLR2A AKAP6 HMGB1 LLGL1 RAP1GAP2 TMEM107 NDE1 |
| hsa-miR-361-5p | 299 | CALM3 PKIA GPR155 ZHX3 GPHN OSBPL11 PCDH9 GPR85 ZMYM4 CAPN6 FYTTD1 CNTN3 UBR5 TMEM64 WT1 MAP1B IGF1 NHS SLC1A3 ZFPM2 VEZF1 SEC63 EHF C7ORF53 NUFIP2 CTNND2 PEX5 MBNL2 ADD3 PELI2 BEND6 KIAA0240 P4HA2 EPB41 PHF17 BRUNOL6 CDC123 AFF4 GLRB ACAP2 ERG TRIM33 USP47 C15ORF29 SLC16A9 IKZF2 CHD9 TMED7 MET HERC4 PTPRE ZFAND1 AFF2 SYNPO2 CREBBP ARHGAP6 RIMS3 MMD TSPAN12 ZNF516 TNS3 ACTR3B NR4A2 HBS1L HMMR MED13L UNC5D KDELC2 CPOX POLR3G ETV5 CTNND1 GTF2E1 SETD7 XRCC4 IMPA1 KIAA2022 ARPC5L MYCBP RAD23A RAB3GAP1 DUSP6 SERP1 DIAPH2 EPHA7 BNIP3L MECP2 NTRK2 ZNF294 PIK3R1 IL10 LIN54 PLCB1 ADCY2 LRP1B G3BP2 CLDN8 CUL5 TSC1 ARHGEF12 MLLT6 PCGF5 SH3BGRL2 RCHY1 PRDM2 NFIX HISPPD1 KCNA1 PKP4 YAP1 YIPF4 SC5DL DIP2B BCORL1 EIF3A C3ORF24 RAD21 C17ORF39 PIGA ZNF238 FGF7 REV1 RPGRIP1L CHP RBM16 ZNF362 OXR1 DCBLD2 RAD23B MTRR SOX11 TGFBR1 DICER1 STXBP6 SP1 MS4A2 C3ORF58 MXI1 PDS5B DHTKD1 EBF1 ZDHHC9 FOXM1 MTMR4 IL12B ARRDC3 FAS KLF12 RAB28 NFAT5 FAM46A DDX3Y BDNF PRY GALNTL6 HOMER1 MYT1L GIGYF2 KLF13 C18ORF34 OTUD4 PAP2D HS2ST1 RNF11 WNT7A SOHLH2 GRM1 CYYR1 GCOM1 MMP16 DEXI LCA5 MEOX2 ABHD2 CLIC2 KPNB1 MLF1 PDE3A NUP153 MYH9 PPP1R9A PPIL5 PLEKHB2 UBE2K RPA1 PLP1 KCTD6 ZNF804A ZXDB CSMD1 VEGFA SLC4A7 FAM96A LAD1 TSPYL4 RBM25 PPM1E ARCN1 SLC31A2 MAPK8 RELN TRAF3 MACROD2 GATAD2B MEX3C TRPS1 CAMK2D SLC5A3 SND1 CALCRL C8ORF33 DGAT2L4 ABHD3 ADAMTS5 DYNC1LI2 LGR4 TNFRSF21 UBE2H DAG1 APPBP2 ATPAF1 RAB11FIP2 ACTG1 BTF3 MTUS1 DDX3X KLF9 LEMD3 RANBP17 MAGI1 AHCYL2 ARID2 KCNJ3 RCAN2 LIX1 DPP10 GOLGA4 CCDC58 DTNA ARMC8 STEAP2 AEBP2 TFAP2B MMAB RHOA MCL1 GPM6A NEBL MIB1 CBFA2T2 CNOT6 ZBTB10 ARGLU1 C9ORF150 ZNF521 MAP2 RUNX1 HNT KLHL7 SUV420H1 CLPX RRAGB OSBP C2ORF67 SNX3 NAALADL2 PRICKLE2 ZBTB40 GABRA1 KCNA5 REEP1 FAM13C1 FLRT2 SEC31A DR1 PPTC7 SHOC2 SFRS12 CRY1 PSD3 PDE4B ODZ4 FBXO33 BBX CREB5 ZNF507 ELOVL7 CSNK1G1 ZADH2 CNOT6L SGPP1 USP9X PCLO YWHAB ACVR2A PARP11 RNGTT ZDHHC17 MTFR1 |
| hsa-miR-222-3p | 433 | SNAP29 FERMT2 DPP8 SLC35A4 ASPH CLDN12 ERBB4 SEC62 CPNE8 STK24 ZC3H6 TFEC TMEM25 DCX MYBL1 ACVR2B PPARGC1A PHACTR4 ZCCHC16 PVRL1 FAM178A RANBP10 IGF1 AMPH DDX42 HOXB5 KCNK2 KDR CABYR KSR1 SSSCA1 ZFPM2 CHD8 VEZF1 CAMKK1 PAK1 GARNL1 DNAJC14 HAS3 ADD3 CDK6 ANXA1 sep.14 MSL2 GRB10 AGFG1 VAPB NAP1L2 BICD2 MLL3 PPP6C BMF PCDHA10 MYLIP SHANK2 PAIP1 NSMCE4A PRKAB2 TAF9B SERPINB5 KLC1 KBTBD8 ANO1 SMC2 HNRNPD SLC25A37 HLTF MEIS1 AKAP13 SOD2 C12ORF36 AMMECR1 SLC39A10 DLG2 CBFB YWHAG RP11-35N6.1 GNAI3 SNX4 PDGFD IGF2BP2 CD4 TRAM2 FOXJ3 CLEC16A DYRK1A RIMS3 BBC3 PCSK1 SOX4 ANGPTL2 PRUNE PCDHA5 HCN4 SLC1A2 RIMBP2 PCDHA1 TUBA1A MIS12 ATXN1 FMR1 WSB2 C5ORF24 GTF2E1 UBE2E3 SETD7 ATAD2B CCDC50 GNAI2 SOX10 CDV3 PTGER2 CHSY1 PCDHAC1 HNRNPA3 INSM1 IRX5 CCDC64 KIT BCL2L11 USP6 CANX AIDA BNIP3L LCP1 MECP2 PCGF3 OSBPL3 SP100 PIK3R1 YAF2 PTPN3 ZNF323 PCDHA8 TMEM55A TIMP3 USP6NL PPP1R15B PAK7 MPZL1 SLC30A8 CD47 PAIP2 NFYB KPNA2 NOVA1 APAF1 MESDC1 ZFHX3 ASXL1 DACH1 INA SCOC WDR37 OLFM4 SPCS2 CTDSPL2 PPP3R1 ATF3 RAB1A EIF5A2 EIF5 RND2 CDON SC5DL SLC17A6 WHSC1 EGFLAM CDC42SE2 SLC2A13 NLK BMI1 MLL GALC SEC24B ZHX1 ZNF654 SIRT1 CD164 PRKAR2A UBE2J1 MIA3 SOX11 DICER1 CBL PCDHA13 SNAP23 SYT10 ITGB3 SKP1 RTF1 SEC24C AXIN2 MIDN FRAT2 GAS8 FOXN2 NEFH PCDHA4 GABBR2 FUSIP1 MAGI2 TLL1 TMEM132B RBM24 ST6GAL1 HECTD2 DCUN1D1 MBNL1 SLC17A4 MEGF9 UNC84B PURA DCUN1D4 TOX ERBB3 RNF4 ESR1 C14ORF147 KLF12 PCDHAC2 ARNT NFAT5 MARK1 DDX3Y SHOX2 OSTM1 WDR40A CDKN1B TRIM2 DMRT3 CALB2 FOS NAP1L5 MAP3K10 TMOD2 ASB7 PPP2R5E PLCXD3 OTUD4 ANKHD1 ADAM22 ZNF25 MIER3 SMARCA4 KIAA1370 C12ORF30 BCHE WDR35 TTC8 ZNF385A RALA CPEB3 CEP55 RAB18 CAMTA1 PCDHA12 SLC40A1 PPP1R8 HOXC10 CLIC2 SPRED2 PLCL2 GNG2 SLAIN2 ARF4 CXCL12 WDR77 KIAA1598 STYX TET2 MORF4L1 FNDC3A SOCS4 CORO1A EMX2 SLC4A7 THRB MYEF2 CREBZF PITPNM2 ADNP2 DLX1 OLA1 RFX7 GOLSYN PPP2R2A EIF3J C18ORF25 POGZ TRPS1 CAPRIN2 FBXO28 SUPT7L ITPR2 DTX3 ATPBD4 EFNB2 SPATA2 RECK C6ORF134 PHF2 THBS1 ALDH1A1 PELI1 NRK ADAM11 BCL11B DYNC1LI2 FAT2 SOCS3 SCARB2 THAP1 PRDM10 SRGAP1 TSC22D3 PCDHA7 KIF16B APPBP2 CAMSAP1L1 NXPH1 PCDH10 MAPK10 ARRDC4 KIAA1267 CUX2 ETS2 TMCC1 USP32 QKI FIGN KHDRBS2 TMEM168 SLC4A4 MAGI1 HOXD1 ETS1 SEMA6D TCF12 IKZF4 NDUFA1 PCMTD1 CSNK2A1 CASZ1 SCD5 GNG12 RBM5 MAP4K5 NTF3 MOBP HNRNPH3 WDR6 CYR61 MBD2 C7ORF41 PDZRN4 MYNN VASH1 RLBP1L2 CTCF CDC2L6 C2ORF71 PANK3 GPM6A KPNA1 EDEM3 ZNF655 SMC1A BEND4 CCDC18 LOC91461 LNX1 MMP1 HIPK1 INSIG1 MYO10 MAT2A RSBN1L PDCD10 RAP2B TRPC3 CHMP4B ARID1A PBX3 GMFB KIAA0494 SMEK2 TFG ZSWIM4 FGF5 ADAMTS6 TP53BP2 BRD1 PRICKLE2 PAK2 STMN1 GABRA1 CYP1B1 VGLL4 C6 BRWD1 SMAD5 PCDHA6 ZEB2 TIAM1 SBK1 GOLPH3L PCDHA3 CNR1 IRF2 EIF4E3 PCDHA2 CREBL2 BBX UHMK1 NSUN4 TNRC6B PHF15 SNCB ZADH2 CCDC88A PCDHA11 ATP1A1 ACTR2 NAP1L1 ZFYVE16 ACVR2A HEXIM1 ANKRD52 CDKN1C SPOCK1 WDR47 GNAO1 |
| hsa-miR-26b-5p | 2987 | FSTL1 FARP1 CIPC KCNJ2 VPS33A RALYL TUBD1 GNL3 POLR2G BTG2 ITGA5 RXFP3 APBB2 MUM1L1 XDH MTERF PFKP COX7A2L TESMIN ANKRD36B PMPCA PDCL3 RNF38 AEN GFOD2 CXCR4 SALL1 GLTP SLC7A6 NAGPA CRTAM ADAM12 IGF1 HSD17B2 LMAN1 NT5DC1 GALNT3 UBE2G1 PAK1 UQCR11 PELI2 sep.14 SIDT1 EPB41L4B CCNI EP300 NKX2-5 NTSR1 MITF MESDC2 HS3ST3A1 RTN1 CENPQ GYS1 OR2W1 CHORDC1 TRIB3 TAF9B DHX35 COL4A5 KBTBD8 SLC33A1 UBB CDK14 G2E3 NWD1 TMX4 CYTH1 FBXO42 MIER1 CHD9 SYCP3 STK16 MSH3 XK SEMA4D RAB3IP MREG BAK1 THRAP3 CERK SPECC1L ING3 TET3 C10ORF137 PCSK1 RAB40B IGSF3 LONP1 SRPK1 MED13L CXCL13 MRPL1 OAZ2 PTCD3 MNS1 SMIM9 CHD1 CXADR ECH1 DARS DIP2A DHRS11 ARL4A ARHGAP21 BLOC1S2 ACTR8 CYP2F1 ITGA3 HERC2 BRCA1 RABGGTA KIAA1033 CDC25B CYP2D6 TMEM19 MAP1LC3C HOOK3 CRYGC GINS1 PRKCB C5AR1 ACSL3 ABCD4 PALLD SNX16 ANXA3 BBS12 TLR1 PPP1R15B CLTA EED KPNA2 A1CF FGGY CA11 SIX5 KIAA0528 ACBD3 VGLL3 DCP1A TMCO3 DSCC1 TAF5 TRAF5 HSPA1L CEP350 CES2 CIAO1 RAPGEF6 DDB2 AARD TSPAN13 STARD5 MKNK1 BCLAF1 SLFN12 GOLGA3 FAM98A ABR FOXE1 BBS7 CD38 KIAA2018 RTF1 RIPK4 ZDHHC6 CTDSP2 ALX1 CCNE1 EVI2A IL17RC TIAL1 NT5C1B-RDH14 LRRC8D FOXM1 BUD31 AP3M2 UFM1 USP14 ZNF385B CCDC41 RASGRF1 ZNF556 FBXO11 ST8SIA4 C6orf15 CDK8 FIGF C3 SRSF6 QDPR ZNF598 PPP1R3D TPM3 SLC6A15 POLE3 HSPD1 CHST15 FPGT C1orf216 PRY SLC38A10 SIPA1 CTSL2 EP400 GDF10 DMRT3 COQ9 ATAD1 NELFE DSCR6 UBR3 LAMP3 NOP9 CDIPT OTUD4 PMF1 FNDC1 FAM98B KIAA1704 ZSCAN16 RFX3 CBLL1 BCHE ENOSF1 CARMIL1 EIF1AX CPEB3 PCDH7 SRSF11 RPLP1 MMP16 ADI1 SCP2 TMEM156 DRD3 SLC35D2 KIAA1324 ABHD2 QPRT TRAM1 RACGAP1 SC4MOL SLC6A6 DUSP5 CHFR ID1 TAF12 GKN1 RAB3A COX8A FAXC OTUD6B ATP11C ZNF35 C9orf16 ERBB2IP TMEM117 ADAM19 FKRP NIPSNAP3B SSX7 PRPS2 FBXO24 THRB DNMT3B MYBPC1 ADRA2A DNAL4 TSPYL4 TASP1 RGS4 SLC13A4 EDRF1 NIPBL ZBTB11 PDE4C POLG PPP2R5A ADAM23 ZNHIT2 POTEG B3GALT5 LUC7L2 SPTBN1 HSF2 MTX1 RANBP6 PAQR8 CYP2D7P1 GRIK2 APOBEC2 LTBP1 GNRHR PCDH18 SLAMF1 RASIP1 DLGAP4 CCDC181 ZBED1 LIF CNTFR POF1B USP32 ENDOG KDM6A ZNF549 ZBTB18 OFD1 FGFR3 PALM2 AGT CLSTN2 TMEM33 LSM12 WDR20 CST3 CDC6 KDELC1 UBN2 FUT4 PIP5K3 DNAJA3 HPS4 SLC10A4 INPP5J FANCC ARL6IP6 TENT5C GPATCH2 NEBL ZNF655 BOD1L2 HSPA8 DIDO1 AGPS ZBTB20 PNOC KRT222 IPO13 PCCB ARPC3 BRD8 SPRY2 PJA2 HEPHL1 GSTP1 HOXD13 DCUN1D5 KIAA0494 GMFB TCF4 NR2C2 AGTPBP1 NKTR CEP85 FXN CHMP7 ADRA1A DCSTAMP NOC3L FUBP1 PBLD AAMDC UBE2W ACTR5 TENC1 FN1 PSMD5 C17orf53 CBX6 LACTB2 FAM126B MAD2L1 EIF4A3 SPDYE5 CAMSAP1 LUZP6 RTCB ATP1A1 ZNF672 NFE2L2 SH3PXD2A TAB1 ZNF275 NEK6 SETD8 KLF4 CYP4A22 PGM2L1 ANKRD52 PAQR4 ZNF805 ACSF2 KLRC1 RPS6KA6 MAGOHB MOB1B SNN SH3D19 IFI16 GALR2 GABBR1 ATP7B LSM3 GPBP1L1 PPM1H KIN CARD14 CHMP6 RALGPS1 SNAI1 BRD4 CCDC6 EFNA2 SPCS3 MANF ARFRP1 CHAF1A ENTPD7 TTC13 ZFPM2 DEFB4A MRPS28 SEC63 FTH1 CUL4B AKIRIN1 DTNB ALDH6A1 NPRL2 MLL3 GRWD1 CSRP1 BOD1 LIN28B TNKS2 sep.10 FANCA CDC2L5 DNAJC11 PCBP3 TMEM248 HNRNPA0 FAM114A2 AMMECR1 PSTK LRRC2 GLDN WDR91 HAGH C10ORF12 RGS20 TRAM2 ARHGEF26 PAWR MTMR12 CREBBP KLHL21 ASTN2 ZNF254 PARK7 DUSP14 FAM177A1 ELAVL2 RPN1 PSMC4 C3ORF63 FAM9B KCNH4 MPC2 NOP10 KIF4A TANC2 SELP POLR3G SNRPC CPD ANXA8 KLHL28 CFAP43 TANK RARS2 PROSER3 FAM193A DNAJB4 TMEM189 CYB5R4 NTN4 FAM120A TRAPPC2 KRT8 SERP1 SYDE2 EPHA7 CNTNAP3B LMAN2L C1GALT1C1 MAP7 EIF2S1 POTEJ GPR135 ZNF294 PIGG TSHZ1 TAF9 TERF2 DGAT1 TAC3 LRP1B BARD1 PHGDH IGFLR1 EPS15 CA9 ZNF710 TLX1 WNT5A KALRN THOC7 TCTA FDFT1 XKRX PTGS2 USH2A KMT5A TEX2 MAP1A NELFCD MAP4K3 HOXA5 BHLHB2 ATP6V1A MICA GABRE INPP5B ANKRD17 ATXN7 ELF4 IL20RA UBE2J1 CHN2 REPS2 HAS2 KDELR1 FCAR PARP4 CD200 MARCH3 ICE1 COL19A1 FLJ37543 ITGA6 ZNF468 BAG3 C1GALT1 OXA1L CCND1 THAP5 EAF1 SC5D SLC9A2 MICOS10-NBL1 CD93 PDCD6IP ZNF136 USP18 SLC30A7 RASSF9 NET1 BMP2 TOX MGP MVD SPATA46 CCDC144A ESR1 GCNT2 GABRG3 SLCO1B1 CCNDBP1 KIAA0232 SMS ERCC8 RRAGC GPX4 ACE2 OLIG3 KIAA0368 GCH1 FBXO3 CHMP3 ICT1 TYMS DNA2 FAM199X PLCXD3 AADACL1 FAM107B ALG9 ANAPC1 PIM3 RPL39 CPA4 HERC3 DMXL1 RBM3 GPR1 PODXL PDE4D TRPC4 CLIC5 ZKSCAN1 ZNF551 GRTP1 ARHGAP26 YPEL1 ITGB8 KLRC4 ERC2 POFUT2 PAPD4 DSC1 PRDM13 METTL16 FRS3 MAP2K4 ITGB2 RASGEF1A FOLR2 TRANK1 SF3B2 RCN1 UBE2K TMEM263 EPB41L3 GAL3ST1 CDR2L BCL6 ZNF480 PSMB8 ARPP-19 LIMS1 ZWINT LAMA1 TBCCD1 OR10H1 CDH11 MFAP3L DERL2 SLC7A11 FHL5 OR10H2 UXT TGFB1 DCUN1D2 SLC6A16 ABHD18 LRRC8E MME DOCK4 GRIN2A CEP68 MAST4 MPDU1 SLC5A3 TRMT2B UBE2L3 SNRNP48 NRXN1 NAA15 NATD1 NR4A3 BLOC1S5 MCTP2 MT1P3 PIH1D3 BCL11B ZNF839 POP4 MEP1A ACAA2 PARK2 TMEM132A TIMP1 PLOD2 KLF9 TRAPPC6A CEMP1 E2F7 VCP YES1 LAMA3 PWWP2A ARID2 SCGB1D2 SRSF3 SP4 PIP5K1C KIF1B GSK3B GNA13 B4GALT4 TNFAIP3 NAA40 ADAMTS17 TGIF2-RAB5IF PDK4 PANK3 RESF1 IBSP FUT8 TRMT11 BEND4 CCDC28A SNURF TESK1 CDH20 PPP4R1 GCLC MAN1A2 TMEM62 ALDH5A1 IKBKAP FKTN POLD4 SSBP2 ADAMTS6 COLEC12 RPP30 BLVRB STMN1 GALNT10 LSAMP STK39 METAP1 APOF TRIM6 WEE1 CKS2 ZBTB24 PLXND1 TP53I3 GREB1L ARHGEF5 RPS6KA2 MRPL22 ACER3 ADAMTS12 ZNF862 BBX TULP2 RFK TNRC6B ATG4A BNC1 FOXG1 ALS2 ZNF608 COMMD8 NAP1L1 PLXNA4 HSD17B13 LOC389602 ECT2 SLC38A6 GRIN3A FPR1 DEPDC1 ASPN GRAMD1C CCT7 USP38 ZFX TRIP6 CDK18 KDM4A URB2 PHAX EI24 GREM2 ZNF548 KIAA1045 ABCD3 HOXB1 CRADD INPP5K DUSP12 CASQ1 GSR BLNK CACNA1C FIG4 FAM20B IGHMBP2 FOXD2 GGH UBR1 DCTN4 ADARB1 CCSER2 INTS7 AMPH AP1S3 ZNF664 GPC4 ASNA1 TMEM178B COL4A2 SRCAP HAS3 ADRBK1 SLC1A6 C1S ZNF84 GFPT1 HMOX1 HOXD8 SLC27A6 ERAP2 DYRK3 NLGN4X NUCKS1 ADGB EIF4E ABHD5 RCOR1 MKNK2 ZNF44 NOL4 TMEM68 BGLAP FOXJ3 MRAS SLC35F5 ZIC5 LPAR3 CCR6 LMNB1 PNPLA3 TSPAN12 CNTLN NRN1 KIAA0408 SLC2A14 SRP14 TFAM ACADM KCNQ4 CDC14B SLC22A23 PPP3CB FAM8A1 TUBB2B CXCR1 USP15 CEND1 ZDHHC7 ZPR1 NAMPT FAHD2B SLC31A1 MMP8 HDAC4 ADAM29 GFOD1 CTNS STK36 SFXN1 CTNNBIP1 FOXN3 TXLNG PFKFB2 YAF2 KIAA1644 MAP3K7IP2 CES3 WDR33 CNPY3 LIAS PLCB4 MRM1 TSKU PDLIM1 G3BP2 RNF216 GADD45A DPM1 GPSM1 IAPP PSMB7 WDR92 PPP1R14B ZNF430 GP1BB TNRC6A TAS2R9 THUMPD3 C1orf50 GTF2A2 TRIM6-TRIM34 CYP27B1 RHOU VPREB1 PPP3R1 PTBP3 EPC2 DMXL2 CCNJ KRT4 SPRY1 NAE1 POTEF MMP14 FMO3 SLC17A6 POU4F3 MDM2 NLK SF3A1 EIF3A COA4 IPPK THSD7A SRP9P1 ICMT KLHL15 GALR3 ABCA6 dec.01 SUSD3 SESN1 APPL2 TNPO1 ENOX1 CMC2 C12orf5 AFAP1 NCAM2 CDK13 PGAP1 ITPRID2 BCR MSRB2 GPR161 AKAP6 CHD2 C19orf52 FZD5 POLR2F TRIM17 USF3 CELA3B PURA GTF2IRD2B CREB1 HOXC4 TSC22D2 RELCH UBQLN3 SMAD1 C14orf37 MYT1L PALM3 UFSP2 TASOR SRGAP2B STAG1 NAP1L5 HSPA14 SOGA3 SLC2A6 SORCS3 SKIV2L2 TRDMT1 HIST2H4B ABCF1 C20ORF24 CD28 DIABLO BAZ2B TAGLN3 CPSF6 FAHD2A ZNRF3 AGL ZDHHC20 GBP1 NFE2L3 DEFA1 OPCML F13B CCNB1IP1 TAX1BP3 MINPP1 PALMD EYA3 TNP2 ENPEP HIST1H2BI OR10H5 ATF2 NUDT19 SLC22A13 TUSC3 RPL28 NOX3 SEC61G C2orf72 IARS TNKS1BP1 TMEM50B MPP6 LOC110117498-PIK3R3 PHACTR2 TBL1XR1 G6PD LINC00483 RPRD1A NUDC SCARA3 PPP2R2A EMP3 SYNCRIP ZNF426 PVALB ELOVL2 CPEB4 PLEKHH1 ESCO1 APOO CAMSAP2 ACSL1 SNAPC5 TXNRD1 KIF27 BRDT SLC16A4 UBE2H SRGAP1 KLHL18 CCNE2 SMPD3 MKL2 DNAJC21 CRIP2 TAZ PDE7A DCAF4L2 NT5DC2 ZNF423 CYTIP ARPP-21 MTDH MAP3K12 UNC13A CHST7 SLC24A4 SLC3A2 SEMA6D PMAIP1 TARBP2 NDUFA1 SLC25A20 PCMTD1 TRIOBP IER5 SYNE1 S100A2 ZNF573 CMTM4 TUBGCP4 ERO1LB GUF1 C2CD2L GUSB DEFB4B KRTAP5-3 LIG1 CRELD1 CHEK2 RASGRP3 SUZ12 GDF11 MRPS16 KRT18 PI4K2B KIAA0922 GRPR TMEM74B ZBTB10 HTRA1 PDZD2 TCEA1 BHLHE40 MCC RAB32 SLC26A2 P2RX2 RNF111 CAMK4 CHST12 KANSL3 HHLA3 NME5 RB1 CNIH4 CSPG5 PHLDA2 BAHD1 MPV17 GAS2 ERLIN1 RNF24 DGCR14 CACNB2 GPX8 METAP2 NMRK2 RHOQ PAN3 GLRX CELF2 SHOC2 TMEM200B PDHX NTNG1 ITGAX ZNF720 CDK2 MT1HL1 NR0B2 NABP1 MTPN SSR1 TFAP2A TENT2 MYOZ3 PDE4B PGR ADAM18 SLC25A36 INTS2 ZNF99 SRPX RAB11FIP1 CSNK1G1 SDC3 RPA3 YIF1A MICU2 MSMO1 RDH14 POLH SGCD DDX17 PDE4DIP TMEM108 EMC6 PKIA IRGC CATSPERB NAB1 GABRB3 TIPRL TRMT10A GALT MED31 GPR17 LRRC20 TACSTD1 ATP6AP2 ZNF106 SLC35C1 IL36RN ZNF772 RNASE1 RSPRY1 NDUFB11 OSBPL2 TMEM64 VPS54 JARID1A ERO1B PANK2 GLS ADAP2 NHS DCAF10 TM9SF3 KANK2 GLI2 ZNF594 RBM41 SCO2 BCL7B EIF1AY MAFG SPAG1 PIDD BICD2 KIAA1024 C1orf53 TAS2R13 SHANK2 GDE1 PRRG3 SPAG8 DAK ZCCHC2 PLGRKT VASP PURB CHIC1 ACAP2 SULF1 RELB UBE2E2 SDE2 HNF1B DTD2 SUCO PBXIP1 MCUR1 CASP9 MAP3K7 TAB3 SRPR EHD1 UPRT NBL1 BAG4 KCNN2 ZNF469 DYNC2LI1 NCK1 SLC2A4RG SLC7A1 MRPS15 PRKCQ EIF2B1 TBC1D15 ADAM17 MAGEA11 LYZ SH3BP1 MYOG FAT1 ZC3H4 KLHL42 FAM118B GATA2 S100PBP SOSTDC1 C12ORF23 TOR4A EBNA1BP2 PDGFRA TRAK1 HELZ AGPAT5 PTPRJ TET1 PTRH2 DMD ADM CTDNEP1 WDR68 LSM2 ADAT1 TMEM50A ABCB6 RABGAP1L CRYL1 LAP3 PPIC OTC NOVA1 RORC RAP2C SOCS5 IFIH1 KCNH7 AZI2 XRCC5 DLG5 MANSC1 TFEB IMP3 INHA SH3RF1 FNDC3B RGS17 MTCP1 KIAA1128 GPR63 LMCD1 RUFY3 NFKB1 CAV2 ATRNL1 MIA3 TMEM135 FAM46C ZKSCAN3 NEK9 ACTA1 PLEKHG1 S100A7 WDR83OS TDRD7 TPRKB UPF3B CXCR6 FKBP9L TUBGCP5 IGSF6 TXNDC4 HPSE TPPP IGF2R ADNP RAMP2-AS1 VIPAS39 FGF21 LCAT ADPGK POM121C LARP4 NSMAF SKIL PFDN4 NARG1 TGFB1I1 KIAA1462 UBE3A LRRC27 PYGO1 DDX25 TLE4 TOP2B TLK2 SH3GL2 SFMBT1 RAD54L2 ZNF207 DNAL1 TRAPPC3 LY6D C16orf70 ZNF823 GRIA4 RNF11 SEC61A2 COL11A1 HTR2A HIST1H1D FOXF2 EPAS1 PRELP OBFC2A RAB18 ZNF860 VAMP7 USP32P1 PITPNC1 MYLK3 KIAA1598 POU2F1 HSPB7 NKX2-2 PLP1 CNNM2 PMP22 APC NXPE3 DPPA4 C14orf2 IFRD2 CBLC COMMD9 RRP15 SBNO1 P2RX7 NARS2 LIN28A FAM127B CASP4 POLR2E KIAA0101 SEC24A TSFM WRB ZBTB25 IL18R1 MTX2 VNN2 MCMBP BRD3 FBXO28 BNC2 AMOT HOXB7 CPN2 CTXN3 NAT1 PCOLCE2 WFDC6 TRAFD1 CYBRD1 CTTNBP2NL MTCH2 MMP10 LBH ZFY FSD1L PPP2R3A LPHN2 HSD17B14 MIEN1 BMP2K STON1-GTF2A1L UGT8 ALG1 CALM1 SLC25A23 C8orf33 MCM8 HEXA NRD1 PHLDB2 ATXN2L TMEM56 RCC1L SESTD1 RHOBTB1 SLC4A4 KAZN CHPF SMAP2 LPCAT1 HACD2 KRTAP5-7 SRRM2 PAX5 MRPS18B AOX1 ZNF682 CYR61 COL1A2 TMEM208 CEBPZOS TRIM36 CAAP1 UBR4 TMC7 ARL5A MYBBP1A MAB21L1 HSD17B6 EDEM3 ZNF697 RUFY2 FAM92A FAM45A SF3B3 PIM2 KMT2C RANBP9 GJA5 SLC17A5 HGF MRTFB FKBP2 SI SERPINA10 KCNE4 ZNF235 RBM48 HSPA12A REPIN1 ARL8B CAPZB RCN2 ERC1 STARD7 SATB1 PPM1D NDUFA5 THAP9 CH25H KAT2B COL12A1 SMAD6 TMEM206 CHST3 VGLL4 MAP3K9 RCBTB2 ROS1 BRWD1 RBMY1HP C1QTNF1 BMP8B TFAP2E PCDHA6 RBMS1 USP27X UQCRC2 RYBP MAEA PHF23 SLC19A2 PSD3 CPSF2 LTA4H SAFB GNB3 HMGB1 TRMT2A TRIM68 MEF2C ZNF652 SENP5 UCK2 RNASEH1 WLS MLXIP RLF KCTD14 XRCC2 C11orf16 B3GNT5 ANKS1A DBNL SMNDC1 DONSON RYR3 ERBB4 CCDC25 OTUD1 LPP HECA RAE1 DFFB NDUFB1 MEF2D RYK PRTG QARS TLX3 BID OR2S2 RCBTB1 COX5A FGL2 ADAMTS18 FAN1 AIFM1 GSTA1 DCHS2 TSTA3 WNK2 LILRA4 EHF NUFIP2 CTNND2 MNAT1 PTEN ANXA1 ATP6V0D1 TAF4B SLTM SUSD5 GTF2A1 COL22A1 RAP1A ATP1A3 MEX3B PNKP NID1 ATXN7L1 NUBP2 ROGDI MATR3 DEPDC1B S100P RASSF3 CILP FCRL6 UGGT1 ZNF85 SERPINB5 GPR107 MTM1 PLCB2 TIA1 RECQL KCNF1 SYNE2 C7orf55-LUC7L2 GALK1 GABARAP TPD52L2 FBXL6 MAGEC3 SSH2 SYNPO2 SDHB TSPAN3 MFSD6 ALPK3 CYLC2 FLVCR1 DUSP22 USP20 DUSP7 DERA TRIM22 ZNF347 RNASE6 STRADB ATAD2B CCDC50 TAS2R10 PHLPP2 CHSY1 ARHGAP17 RFC3 KIAA2022 GPNMB RAB3GAP1 SFPQ NSD2 EXT2 AKAP7 ABL2 ASCC3 MECP2 BCO2 GRM8 RNF141 CSN3 BMPR1A PKD2 RFWD2 ARPP21 TMEM86A EPHA2 RGS3 ZNF704 ARHGEF12 SAMD12 CD55 NMRK1 SNRNP70 PRICKLE3 RPGRIP1 CSGALNACT2 DACH1 INTU KCND1 YOD1 PHKA2 ATF3 TBC1D12 RHD DGKH MPP7 THAP10 MRPS34 SLC2A13 HTR2C TSGA14 PHF3 MDH1 DCBLD2 BTG1 YTHDF3 DPP4 SYT10 RPL7L1 ZNF148 CNOT4 TMEM30B IKZF1 PGRMC2 ZBTB38 ZNF141 PAQR6 OSTF1 GGA3 PCDHA4 ANKRD63 HIST2H4A SNTG1 FOLH1 MAPKAP1 COL15A1 GLRX2 MFAP3 EZH2 EXOC6B TRPV6 RBM24 ARL5B VAMP1 HDAC5 GRB7 SOCS6 LMLN GON4L YWHAE GREB1 ARMC1 C1orf63 KRTAP7-1 PON1 REEP3 C17orf59 MARK1 MGST3 PNMT SEMA3A NDUFS8 USP49 SLC25A16 ABCA1 PAK6 ACBD5 PQBP1 CLEC2B REST TIPARP MIER3 FNIP1 EXD2 PPAP2A PRY2 ULK1 SKP2 PRYP4 TEAD3 SLC2A3 JAKMIP2 ARID3A PCDHB8 CD36 EFNA4 HAO1 TWF1 RBMY1F NUP153 MUC7 LRRC17 NKRF ANAPC13 TMPRSS11E TNFSF15 HSD17B11 SECISBP2 IDH2 ZNHIT1 SUPT5H COPZ2 PAG1 KCTD18 DCLK1 MYEF2 NOP2 ASF1B TAF2 ANKRD28 AGPAT3 CDK2AP2 TRAF3 LETM1 RIPPLY3 SND1 B4GALT1 MPPED1 SLC25A44 RAI14 LRFN3 FKBP14 HIST1H2BC SGCB CTSV FRAT1 ZNF547 GDI1 ZNF81 EFTUD2 CASP8 TAP1 CMC4 CYP11B2 NRIP1 BNIP2 DHX30 CPSF7 SIRT4 FAM135A CDH2 NACC2 PTPRB GPR52 EPHA5 ERMP1 METRN ARF1 TMCC2 TSGA10 TCF12 ZNF721 POU4F1 ERAP1 ARL4C CUGBP2 PLCH2 PTPN20 GPR22 NCEH1 ARMC8 ISOC2 MYNN AEBP2 JAG1 DCUN1D3 HSPA4 PAPPA RNF144A CYP4F8 EMC3 DCTN3 POM121L1P CAPN9 CCL7 PRKCD NDUFB4 TRPC7 RBMS2 MT1M TMPRSS6 TRIM65 TP53INP2 ZNF407 GPALPP1 SUN1 RBM19 PRKAA1 CASD1 FADS2 HSPA4L IL1RL1 SMC4 PLXNC1 FCGR1B ATP2C1 TMEM230 FAM237A MTTP FAM208B KHK PPTC7 SLC12A2 FBXW2 HDAC9 STON2 NYX PARP14 TOM1L2 ZNF410 GRHL2 MNDA MAGEA9 TMEM165 RAB21 GRSF1 ZNF566 PSMC6 CNR1 KIAA1549L KLRC2 ABCC4 CPB2 SSR3 MPC1 TADA3 HECTD4 ZCCHC24 BICRAL MAGT1 PCDHA11 BATF3 NR2E1 EPPIN PIM1 USP9X PCLO RNF2 PIK3R3 USPL1 FCN2 PARP11 TM2D3 ARNTL2 CCL27 POLA1 PFN2 PPP1CC SLBP RRAS2 CCDC109B OXCT1 MTOR ARPC5 SV2B MCM3 FAM149A CDKN2D CDC37L1 NRBP1 STUB1 LEF1 CCDC170 SLC22A8 IL13RA2 SERPINB2 FES CCNL2 CBLB RORB CCN2 STAC2 FAF1 CDK6 ZFHX4 ADAMTS19 P2RY1 TFPT ZNF236 ENDOV ODAM IFT22 TMEM132C ANKS1B GPR126 MTFMT TMTC3 PPHLN1 VANGL2 LUC7L TBC1D2B ACKR4 FA2H DLG4 MAGEA9B EFCAB14 UQCRFS1 KIR3DX1 ZBTB45 ANKRD46 PLS1 CD248 DGKZ SH3KBP1 RAB23 CHST4 ARPP19 COPS2 ZNF516 SMAD7 SOX3 VLDLR BOLA2 LARP1 KIAA1107 PDGFB SAMD8 VIM WARS MPI SNRPD1 NCAPG2 ANO2 NAA50 CLDND1 MAGIX GDAP1 CARS2 PCNA ABCE1 EXOC8 DDX60 RBM20 HSD3B1 SUPT3H PWWP3B JHDM1D GABRB2 PEX13 ATP6AP1 ARHGEF39 ETV4 SLC38A2 PPP2R5C CD2AP GID4 SMN2 MYH10 TTC39C PARPBP SPATA6 RUVBL1 ADAM9 MYLK4 STX11 DDX52 CLVS2 APAF1 STXBP5 FAM212B SLC36A4 HSF4 NEK1 CWC27 MATN3 IFNGR2 USP25 DENND4A PRKX NDUFA4 ARMC7 ITPR1 NSL1 PCYOX1 DUSP1 CYTH2 BABAM1 NPR3 PCK1 PLEKHA7 FAM168B RAD51C CPM CYB561D2 MEGF8 PARP6 ADAM10 C7ORF58 NDUFB5 LINC02054 SLC26A4 FBXO5 N6AMT1 GPRC5A DRAM1 SOX17 ZNF259 RNF187 GBP2 ZNF217 RGCC WDR4 FRAT2 VTN HEXB ACVR1C LINGO1 CD97 SETD5 TM4SF18 CRIM1 PTER MPHOSPH8 RRS1 SMPDL3A TNKS CRLF1 SNX2 CTH SCAI MEGF9 MADD ATM PLEKHA1 TUB KLF12 RBM46 KCNK1 ARRB1 PFKFB3 LIG4 EREG SFRP4 IL17RB DCAF7 OSTM1 TMED5 AGTR1 AQR GSTT2 WDR60 IPMK TXNL1 AMDHD2 GRHL3 MTERF4 GALNT7 ZSWIM6 MAN2A1 TNFAIP8L1 ABCG4 SMEK1 RND3 PNRC1 MDM1 OVOL2 MAT2B SRGN VDAC1 CASP7 TRIB1 CCNJL USP53 RALGDS FAM170B DSG1 RBM12B FAM118A STYX LHFPL4 SMAD4 WRAP73 SLC35B3 ERI2 CTSD HPGD FCHSD2 SLC4A7 SGMS1 STAT6 DNAJC12 ZNF195 FKBP1B CREBZF PER3 IL3 MSANTD1 CDKL2 S100A1 RPL6 RHCG TINF2 EPC1 SPRR1A CYP4A11 PRKAG2 SNX20 HIAT1 RAB36 PFDN5 CMTM6 PPT1 GRB14 MLLT3 RP2 KEAP1 DCDC2 HAUS8 JOSD1 NUP50 ZBTB39 NIPAL2 CSNK1A1 FANCF INHBB PRR4 ARFIP2 SRP19 RPS6KB2 TXLNA RAB31 TMEM187 DCAF16 SLC6A14 RNF138 SLC35E2B GTF3C2 DIMT1 MAP3K1 CPPED1 HCN3 DIS3 THAP3 TMEM184B SLC28A1 EGR3 TCEB3B IFRD1 IL12A RAPGEF4 KCTD9 PRR14 EMC7 HEG1 ZNF696 TSPAN5 MCL1 CCL2 FBXL19 KIAA1539 PDIK1L PSMC1 TMEM41B COASY CLASP2 CLTC DFNA5 SRP9 EIF2AK3 PDCD10 RPRD2 SMYD2 PBX3 ACADS CXorf38 IL6 ALKBH4 CPED1 CTGF FGF14 ANXA5 USP37 TMEM265 PRICKLE2 BATF MED23 PCNX1 IVD TRIM58 UFC1 PSME1 EEF1A1 UGT2A1 CHAC1 C9orf114 TOR1B HMGA2 ETF1 ING2 SUV420H2 PSTPIP2 NWD2 UBE2D1 C11orf30 CREBL2 RAB39B NUP210 CASP3 ICK CNOT9 TRAPPC13 COPS7B PDIA5 LHX1 DNAJC6 PODNL1 C2CD5 CLOCK THAP2 IRF4 SPESP1 LGALSL DEFA3 PGM2 ROCK1 KIFC3 STX3 L1CAM NXPH4 TGM1 TAGLN KIAA1737 CPA1 FASLG TK1 ZDHHC18 C9orf64 ZNF140 PIK3R4 UHRF1BP1 IVNS1ABP C1QTNF9B-AS1 TBC1D4 DNM3 ABCG2 PACRG MRPL18 CAMKMT KREMEN2 LSM11 C7orf10 GRB10 BHLHB5 CLSTN1 SREK1IP1 PDGFRL ASXL3 DPF3 PRDX3 HMGN5 ODF2 RBM12 NIP7 HSPA13 C19orf66 BDH1 RMI1 CARM1 PECAM1 LATS2 PTPN1 CBFB SESN3 ZC3H7B IFITM3 SOCS7 DAB2 ASPM SACS TTLL12 SERPINH1 DVL3 PLXNA2 KRIT1 ATP5SL PTPRD OCIAD2 PIAS2 CDK9 PNRC2 TBCC NSF CAPZA1 TMSB15A TBC1D13 LRRTM4 DDIT4 NUS1 DNAJA2 CACNB4 SCGB1D1 OR2J3 LRRC55 CHM RANBP3L CRYAA ATP1A2 BACE1 STX1A CSTF2 ACADSB HERPUD1 FAM105A USP6 POMP HGD BMPR2 CDCA3 TFB2M METTL9 SP100 HCG_18385 UGDH MAPK6 FAM198B WBP5 LGMN GPR183 TCN1 PLCB1 CPEB2 LOXL2 NUDT11 ATAD2 ADM2 CCDC182 NUCB2 TXK DYNAP MTA3 PHF6 HTR1B TAF13 SOX5 RAP1B ADAMTS1 SFT2D3 WIPF2 P2RY6 DGKI FAM136A GPR27 SLC16A6 CFI CDH9 MMP21 PPM1B EIF5 UBL3 TIMM10 AK4 PIK3CG NLN PIGA ZNF238 ZNF23 COL4A4 GUCY1A2 SASS6 TBC1D30 LRCH2 GSPT2 TRIM13 STIL MB DNMBP LPHN1 CDK2AP1 GSTT2B ANXA8L1 DENND1B WDR25 SLC35E4 GTPBP4 MXI1 EIF4A1 DUSP21 TAF1 GINS4 IL1R2 SAMD14 TTC28 C22orf29 MAP3K2 HTT ZNF451 CCDC53 CLINT1 HOXC9 DCUN1D1 C12ORF51 SEMA6C GRIK5 FGF9 ZFC3H1 STX12 PLCL1 SERPINB1 SLC1A1 HOMER1 FUT9 CDH1 PSAT1 TDG PMP2 IFNG PLAG1 CNTRL CREG1 CMPK1 SCN2A CCDC82 PUS7 GTF2IRD2 TP53INP1 DCTPP1 LRRC74B SSH1 GNPNAT1 CAD DOCK10 CAMK2A KCNQ2 SPOCK2 PHYHIPL INSL4 HADH DDX6 TRPM1 CACNG3 TFAP2C TET2 HNRNPM IDI1 CNTNAP3 ST8SIA3 SACM1L EPHX1 BFAR ANKRD36BP1 ARAP2 SLC30A10 UBE2E1 DNAJC2 LDB2 GADD45GIP1 NECAB1 FEN1 IGF1R CPNE3 PURG MTHFS ANKRD50 CALCRL IPO7 CXCL9 CAV1 RBM47 NLGN1 KIAA1333 FMO5 HECTD3 CEBPG STRBP FGF18 BRSK2 TNS4 TMEM260 LHX6 GBP6 NAV1 PATZ1 JARID2 ZNF584 DBF4B ATPAF1 JAM2 RAD54L MICAL3 NCBP1 PRR5L CELSR1 SNRPN FGFR1OP2 CACNA1S PRR7 ABCD1 TMCC1 LRRC40 SRPRB CA2 TPO CRYZ TTI2 FAM172A LOC100144595 FGD1 MARC2 ASNS IL22RA1 CASZ1 LRRC37A3 TVP23C-CDRT4 C1D TMEM131 USB1 PDHA2 SPC25 SFTPB WDHD1 UBE4B SH2B3 ST6GAL2 TRIM44 KIF1C CDK5R1 BTN3A3 TRAF1 COMMD3 SLC45A4 SHFM1 SCAMP1 WNK3 TSEN34 SLFN13 DUT SLC26A9 C1QB KIF18A MAP2 ADCY6 TNFRSF11A PRLR TMPRSS11F CDC73 MINDY2 KCNA5 STOML2 HSF1 ALPI HOXA9 TNRC6C LNX2 ZEB2 PKN1 POLR2H ITM2A ARHGAP44 MED9 MAP3K7IP3 SNORA70 MGAT4A KIAA1468 NSA2 USP48 MLLT10 PHF14 GABRA4 ERCC6 MFSD12 FAM3A TMX1 KLHDC5 PDE11A MRPS2 CADM1 PRYP3 ZCCHC8 INPP5D ZNF708 PTPRO ARID3B MEAF6 MAP9 CDKN1C MRS2 PPAT KLHDC10 SYCP2 OPRM1 NIPA1 IL7R APCDD1 TLR4 PRSS16 PCCA FAM84A RAB30 MPDZ OSBPL11 PCDH9 GNB5 MKRN3 ITFG1 MSTN IMPG1 UBTD2 ACVR2B IQCJ DEPTOR C18orf25 PPP2R5D SMN1 MAP1B POU2F3 AKAP5 NVL RANBP10 ATAT1 SFRS18 ACVR1B BRWD3 RBMS3 CCDC15 ROM1 UBA5 DTL ALG3 TMEM194A DNAJB5 RNF186 MBIP CREBRF COX2 RABL3 RSAD2 NHSL1 SMG6 INSR MLEC MST4 RGS6 PDK1 CIB1 OAF COBL RNF212 CBY1 ZNF680 MTERFD2 MDN1 OXSR1 REEP4 RAD51D ACN9 RASAL2 C12ORF35 DYRK1A KIAA2013 NFKBIE MMD PHF20L1 RAB11A MLANA SCAND2P TMEM30A WISP3 DENND4C TCF7L2 RNASET2 SOWAHC MORC4 FAM227A CACYBP TCTN2 ARFGEF1 SETD7 CDK19 CDV3 BTBD7 ZNF800 GALNT11 AUP1 MYCBP VANGL1 GPX5 ULK2 LSM1 PPP2R3C HIPK2 SLC25A28 CCND2 CAB39L CHERP AKT1 ZNF462 PEX11A PTPN3 CXCL6 SLC34A2 ANKIB1 RNF125 NPTN FRZB ART3 RPGR OR1A2 SLC6A9 SLC5A1 MLNR CXXC4 MYPOP UNC93B1 ZFHX3 GMNN TJP2 RNF149 RSRC2 FDXR RAB27A TXNRD3 ERCC6L YAP1 EDN2 PSMD7 SSX2IP LRP4 ST8SIA2 RRAGD HLA-DQB2 ENC1 ORC6 BCL3 OR2F2 HIVEP3 SIX1 ZBTB44 PTP4A1 BMPR1B ZMAT4 CARF SHC4 MAP7D3 UBP1 TTPAL CST2 COL10A1 OAZ1 HIST2H2BF YIPF5 BCL11A TMEM254 HSD11B1 S100A7A NAA25 SV2C KDM5D CEMIP2 GMDS MIR22HG ZNF326 LRRC16A UBE2V2 COL5A1 C9ORF40 KIAA0947 PAF1 PLAC8 DERL1 FKBP9 TMEM2 C1orf94 TNFSF9 DCTD F3 EIF4G2 ZNF37A ROD1 CTSZ FICD IGFBP4 KIF21B UBE3C GMNC PDSS2 ATE1 CLIC2 ETNK1 KLF10 MFSD14A TEX30 TTPA ZNF12 sep.07 PEX5L FAM44B ZNF324B NMD3 WNK1 ARL17A LARP4B MED22 RFX7 TSHB SALL3 MFHAS1 NKX3-2 CSRNP2 LOC203547 VPS13C KPNA6 TRPS1 HMGA1 ACOX2 GEM SERBP1 CA1 MFSD9 STAG3L4 CYP4F11 AGMAT JUN SERPINI2 NAGK DHODH B3GALT1 ATP2A2 ONECUT2 ZKSCAN7 CAST ADGRG7 LGR4 POM121 TTK TGFBI SULT1B1 DDX3X TAOK1 CDH4 TMEM168 ZNF133 CISH GABRA3 GPR39 RNF128 PHTF2 PTPN13 SLC22A9 SNAP91 LRPAP1 RPL37 FAM160A1 DAPK1 RAB5IF NCOA4 CNPY1 RPS6KC1 CDH5 STXBP3 WHAMMP3 CYP24A1 SCML4 NDUFS7 SELENBP1 GGA2 MIB1 TRIB2 INPP5A COQ2 SMARCE1 GHSR SRF HIPK1 MDH1B KHDC1 ICAM5 MAT2A TRPC3 TMEM106B MARCH1 WAPAL SFRS11 NPTXR SLC10A3 SAR1B RNF6 BOLA2B DPYSL3 SCN9A SCN5A VAV1 ARMCX2 DPY19L1 PAK2 SEC22A ATP2B1 GATA4 TOB1 MNX1 C8orf44 TCEAL1 IFI44 PHF21A FAM49B LANCL1 WASF2 FGF23 ELAVL3 PCNT PATL1 EAF2 USP3 ST18 FAM55C FRMD4B FBLN5 HAPLN1 KRT32 TNNT1 KLHL24 PDE12 CNOT6L ARNT2 ATP5MC3 RAB8A TOP1 EXOC6 DCAF8 RNGTT SLC39A6 TP53TG5 ADIPOQ RXRB |
| hsa-miR-532-5p | 184 | RPS6KA6 RNF19A COL24A1 CCDC43 TRIM2 FAM116A PLAG1 OSBPL11 PCDH9 DEK HSF5 ZMYM4 NUAK1 IRS2 ADAM22 GRIA4 PRPF4B XPO1 FSTL5 RAVER2 MAP1B CPEB3 SLC39A8 BACH1 VDAC3 LCA5 ITGB8 CTNND2 SPRED2 HEBP1 PRKACB CYCS ZNF536 RPA1 FAM108B1 FZD7 ZC3H15 ZMYND11 TLL2 CHL1 TTN DCLK1 BCAT1 TIA1 SULF1 HMGB3 RBM25 RCOR1 OLA1 C1ORF9 GOLSYN NR5A2 HCN1 SMARCC1 CHD9 MACROD2 PAPOLA SLITRK1 GATAD2B MEX3C PDE1C PURG CAMK2N1 CIT BNC2 FGFR1 KIAA1486 EFNB2 MTSS1 EXOC5 PCSK1 HOXB3 MLLT3 RBBP7 GATA3 ZC3H12C ELAVL2 RAB11A CRIPT FAM160B1 RAI2 PLS3 ATXN1 TEAD1 TMEM57 LRRC4 CPEB1 MKL2 SLC25A14 ERO1L UBE2E3 SNAI2 RASSF5 CDV3 HNRNPA3 NDP SESTD1 CCDC64 PRSS23 KIAA1033 KRAS ARID2 BMPR2 ZFP91 SLITRK6 RUNX3 ADIPOR2 TWIST1 NTRK2 FOXN3 HMP19 SRRM2 TSHZ1 MAF GPR137C POU2F2 ARMC8 SUCLA2 SNX16 PPP1R3A KTN1 PAK7 PDIK1L CXXC4 NUDT3 SYT11 TMEM70 SLC8A1 GRK5 XIAP ZFHX3 MAP2 CTDSPL2 CAMK2G CLPX YAP1 ATP2C1 SC5DL MPP7 NFIB NEGR1 CDC42SE2 PCDH20 RERG USO1 COL12A1 DLGAP2 SEC24B SF3A3 CXCL2 NBEA PDLIM5 RPS6KA3 SLMO2 OXR1 MAP3K7IP3 LIN9 CHST9 CDC42 SLITRK5 PDS5B FAM113A PAPOLG EYA2 SERTAD2 THSD7B ALS2 ZNF608 CACHD1 MTMR4 SH3PXD2A TULP4 VAMP1 LYPD6 HECTD2 SIAH1 FRS2 SLC25A32 DHX9 PURA ZMPSTE24 KLF12 SGCD KDELR2 |
| hsa-miR-27b-3p | 1043 | PTGER3 ANKS1A PKNOX2 BTG2 ITGA5 ATRX MMGT1 RNF38 SOX7 GLTP IGF1 TMSB10 CYP39A1 GALNT3 EPB41L4A GARNL1 EHF MBNL2 FRAS1 MSL1 PELI2 AFF1 STYK1 AGFG1 CCDC46 SENP1 ID3 DEPDC1B FCRL6 MMP13 IGF2BP3 KBTBD8 ERG HMGB3 AP1G1 TOPBP1 ARRDC2 ZBTB34 CCDC28B RP11-35N6.1 BAK1 MET TRIM23 GOLM1 BBC3 C10ORF137 SERINC3 MED13L GIMAP8 KIAA1199 HOXA13 TEAD1 CEP135 LEP C7ORF42 DKK2 RPS6KA5 ATXN10 MRPS14 SFXN2 KIAA1033 C15ORF27 AIDA PCGF3 PSMA1 RXRA RNF141 GRK6 BMPR1A RELT EPHA2 PAIP2 SP6 SLC8A1 MAN2A2 ISL1 RAB33B SFRS1 DCP1A EML1 KPNA3 ATF3 TBX5 UBE2F PTGDR C1QL3 KIAA0409 MLL GFPT2 TSGA14 NEDD4 FAM98A GDF6 DVL2 BTG1 ZNF148 KIAA2018 GTF2H2 CHST1 ZNF280C GATA6 CABP1 PF4 VAT1L SOCS6 ST8SIA4 ING5 GRB2 GLRA2 HSPD1 MARK1 PRDM16 LCOR HBEGF PRPF19 TRIM2 OPHN1 ABCA1 PAK6 HYOU1 SEMA3E CSF1 GCC2 ALDH4A1 FAM105B MIER3 YWHAQ PPM1K MEIS2 CPEB3 PCDH7 ARL13B CAMTA1 GPR114 MAP3K14 WDR43 ALCAM FAM76B C20ORF12 PLCL2 SLC6A6 PDE6D DUSP5 PRKG2 NDUFS4 MUC7 NUP153 CDS1 TTYH3 ATP11C SLC12A6 AICDA ADAM19 FAM133B THRB PAX9 USP42 ZNF187 AGPAT3 MAGI3 CNOT1 EDNRA RAB14 GALNT5 CAMK2D IRS1 COL21A1 SGK269 HOXB3 THAP1 SNX25 APPBP2 LIF PRKD3 CCM2 SDC2 RAB11FIP2 RCOR3 NRIP1 ZNF395 ERO1L NRP2 RASSF5 H3F3B QKI NCAM1 SLC38A4 CASC3 ECE2 PALM2 FREQ LSM12 KRAS CUGBP2 ARL4C ITSN2 RCAN2 GNG12 FGF1 CHRM1 PPARG ARMC8 NFIL3 C7ORF41 APLNR TFAP2B CNN3 GCA PAPPA RNF144A EPHA4 INPP5J MARCH5 EVI5 PRKCD TP53INP2 STK40 ANTXR2 PRRG1 MED14 AGGF1 EFHA2 SPRY2 MAPT RET RUNX1 SUV420H1 ENAH HDHD2 NBPF3 NKTR FRYL ASAH1 ZNF350 EPB41L1 LITAF UBE2W CDR2 CDH24 FBXO36 KIAA1012 FN1 CNR1 PHLPPL RSU1 FAM126B MYT1 FBXO33 FAM171A1 RSPO2 MSI1 ZCCHC24 SERTAD2 UBE2N NFE2L2 NEK6 C10ORF114 ACVR2A PGM2L1 C17ORF85 C1ORF83 SCN1A C9ORF91 OPA1 PFN2 SNN PAXIP1 PPP1CC WNT3A ACCS TMEM25 OSBPL10 DCX PPM1H UBR5 RNF182 RALGPS1 SNAI1 EYA1 MAPK14 HOXB5 SCAMP3 CCDC120 TPT1 C1ORF52 CBLB MYPN SLC35F1 SATB2 CDK6 AKIRIN1 ZFHX4 VAPB MLL3 KIAA1787 HOXB8 SNAP25 ZFP36 ADCY3 FZD7 GPR126 LIN28B VANGL2 APBA2 AFF4 SAMD10 ASAP1 C15ORF29 NGFR C17ORF63 NGFRAP1 EDAR NHLH2 FYN ANK2 SH3GL3 RIMS3 LDLR VEGFC ELAVL2 RPN1 SLC1A2 FAM160B1 PRMT8 CPD MEPCE HOXA10 BRSK1 SERP1 JHDM1D SOLH EDNRB RAB20 RNF12 KIAA1147 MFSD2 PDPK1 SRGAP2 PHC2 PANK1 ST3GAL6 MYH10 EPS15 APAF1 THOC1 TMEM9B AQP11 SH3BGRL2 GNS MATN3 USP25 EMR2 MKLN1 PRKX CBX1 EBF3 EIF5A2 NF1 LRP5 NEGR1 INSM2 LYPD3 BCL7A ATXN3 HOXA5 NAV2 RABGAP1 ATP6V1A FGF12 TGFBR1 FAM78A CCNK CNOT7 FAM53C CDC42 FOXN2 RNASEN ACCN2 COL19A1 PDS5B C1GALT1 SFRS2IP HNF4G SIGLEC1 RS1 SEMA7A ACLY KLF12 ARHGAP12 SURF4 DPY19L3 MAPK7 NFAT5 TMED5 ALG9 CABLES2 GALNT7 MAN2A1 SH2D3C KLHL4 ANKRD40 RND3 NAT13 IL1RAP MTMR10 CSNK1D NR1D2 ITGB8 ERC2 TXNDC5 PTPRT PDE3A FRS3 MAP2K4 STX5 CDR2L WSB1 NXF1 SOCS4 FCHSD2 PCTK3 PLEKHH2 ZWINT SGMS1 SEMA3B MANEAL CDH11 SLC7A11 PITPNM2 ZBTB41 SFRS8 PPM1E ITGA2 VAV2 PTCH1 SHANK3 SLC5A3 GREM1 AK2 CHST2 SPATA2 NRXN1 BCOR H2AFZ CCNT2 UNKL BHLHB3 SLC35F3 ZBTB39 RUVBL2 SULT4A1 POU3F2 ARRDC4 PTPN9 ABCA12 CCNC CHKA E2F7 MAGI1 YES1 ARID2 MDFI LASP1 PLAA HUNK EGR3 GPD1L AMOTL2 ALS2CR4 C6ORF120 FOXO1 CTCF HEG1 MSTO1 sep.08 FUT8 C8ORF4 BEND4 FAM104A ST6GALNAC3 DOT1L RSBN1L RAP2B SEC61A1 EPHB1 ANXA5 ADAMTS6 COLEC12 FBXO30 PRICKLE2 HABP2 DLL4 SEMA6A PTGER4 STK39 SMARCA1 LIMK2 WEE1 NR2F2 SPAST RGS1 UBE4A PLXND1 SMURF2 SRL CCDC47 UBE2D1 FAM84B PDE7B NUP210 DTX4 TNRC6B FAM120C SEL1L CACNA2D3 PPP1CA ZNF608 CA7 YWHAB GRIN3A GLCCI1 PDIA5 KIAA1109 SMOC2 GZF1 AP1GBP1 ZDHHC17 PKN2 HMGCR IRF4 HORMAD2 C18ORF1 GLT8D3 SLCO4A1 KIAA1045 SLC25A25 PDPN KIAA1737 PDZK1IP1 ZNF329 FOXP4 FAM73B MDGA1 UBR1 DCTN4 C1ORF21 GPC4 KCNK2 UBE2NL SYT1 LSM11 GRB10 FNDC4 FAM126A FOXP2 NKAIN1 EPB41 NLGN4X RARA FAM108C1 SS18 C5ORF41 CSDC2 MKNK2 IKZF2 C1ORF9 C1ORF144 SMAD2 NOL4 CBFB RMND5A ZIC5 MARCKS COL11A2 PPARA F13A1 SHE GATA3 MN1 MOSPD3 CDC14B CAPZA1 KCNJ1 PLK2 KCTD4 KIAA0247 MOBKL1A NFASC PLCH1 ATP1A2 BACE1 STX1A NAP1L3 FAM102A BMPR2 NTRK2 FOXN3 TMEM182 MIER2 sep.11 MAP3K7IP2 AGRN IL10 USP31 HIVEP2 FAM65B P2RY5 NPEPPS CHD7 TSC1 MESDC1 RAP1B OTX2 WIPF2 RAD9B C1ORF115 SLC6A1 PPP3R1 USP46 CCNJ EIF5 STIM2 NFIB NLK NLN BCORL1 KIAA0182 SPTY2D1 FGF7 ZNF238 RPGRIP1L HNRNPF LHX4 EGFR GSPT2 LIFR SP1 TOX4 TNPO1 ENOX1 MAPKAPK3 MXI1 AFAP1 PGAP1 CLCN5 ARL4D PPME1 MAGI2 MTMR4 HTT NECAP1 TTC39A FADD DCUN1D4 ATP5G3 CREB1 ABCB9 NIN SRPK2 TSC22D2 SMAD1 NEO1 DDX3Y CFL2 STAG1 TBR1 IFNG NAP1L5 TBC1D1 PLAG1 CD28 BAZ2B TP53INP1 XPO1 KIAA0146 CAMK2A CCNG1 WDR7 ENPEP AMMECR1L SLC39A13 TROVE2 FNDC3A CA10 SFRS9 C9ORF80 ZNF346 DNAJC5B GABRP NR5A2 SLC27A4 PPIF EHD3 TMEM167A MED13 CPEB4 ACVR1 FAM134C PLEKHH1 EFNB2 PID1 TMEM35 STRBP C11ORF57 PATZ1 BAG2 MAP2K7 TMBIM6 DNAJC27 VIP ATPAF1 PLAGL2 SMPD3 MSI2 MBTD1 TMCC1 ELMO1 FAM184A MAP3K12 TGOLN2 DNAJB9 FAM172A ANK1 C6ORF218 DCP2 PMAIP1 SEMA6D GSPT1 RGL2 NCOA1 CNTNAP2 TRIM44 PPAP2B SUZ12 CDK5R1 DLGAP3 ZBTB10 KHSRP ADCY6 KLHL31 PRKY LHFP RNF111 KIAA0895 TARDBP ID2 TMEM189-UBE2V1 FGF5 SLC9A4 MED28 LOC440093 ADAR BAHD1 FLRT2 PPP4C CACNB2 EN2 CAB39 REPS1 STXBP4 ST14 MAP3K4 RIT1 ZEB2 PDHX FBXW7 CLCN3 SFRS12 GPAM MAP3K7IP3 JMJD1C RAB11FIP1 PHF15 CSNK1G1 NEDD8 SSTR1 NR2F6 ADORA2B CTDSPL SAP30BP CADM1 SOX6 PTPRO TFPI ZFAND3 TMUB1 CALM3 PKIA ZNF100 NRIP2 EIF2C2 GABRB3 OSBPL11 MS4A7 KTELC1 ZMYM4 GOSR2 MSTN RAP2A ACVR2B FLRT3 SYDE1 B4GALT3 POU2F3 CECR6 SFRS18 NHS RNF139 TP53 RAPGEF2 TMEM194A ATP6V1G2 MNT ARHGEF6 INSR GLT25D2 RGS6 CHIC1 SRGAP3 PDK1 FGF13 NCALD PHF13 NFX1 SLITRK1 PTGFRN PBXIP1 CIT SLC35A3 AMOTL1 C12ORF35 DYRK1A GTF2I NEUROD6 MMD LPL RBBP5 CLEC2D HCN4 BRPF3 UNC5D ATXN1 SLC39A11 C12ORF34 ARFGEF1 GPD2 SETD7 FBXL11 ZNF800 ITCH MYCBP SEMA4F JMJD1A UNC13C CSRP2 GATA2 N4BP1 CHERP CDC42BPB C3ORF65 GRP PTPN3 SV2A HOXC11 PDGFRA TET1 PLEKHJ1 MAPK8IP3 STAC C1ORF173 E2F6 NOVA1 TMEM126B ZFHX3 EFNA3 WDR37 RAB27A ARF3 EYA4 PON2 BMI1 SH3RF1 EIF2S2 RGS17 TAPT1 ENC1 ZHX1 ICOS SS18L1 ANK3 FOSB SLFN5 C2ORF55 BCL3 SOX11 SHC4 PDE8B C16ORF5 UBFD1 TRIM46 VGF PIP5K1B RNF8 SLCO5A1 UBE2V1 ADAMTS10 CALD1 C5ORF13 LARP4 BCL11A NARG1 HMGCS1 KITLG TLK2 ELL2 RPN2 RIMS4 RSPO3 TXN2 ACTA2 DERL1 GRIA4 F3 ZZZ3 ZNF385A SFXN4 PAQR9 STX16 OBFC2A BEND3 HOXC6 PHB SLC13A3 KPNB1 NRBF2 CCDC92 LPIN1 NCOA7 LONRF1 LIN7C NXT2 APC FAM108B1 HIP1 GALNT1 BCAT1 FZD4 WNK1 MUTED STX6 LIMK1 SEC24A MFHAS1 RELN TPR KPNA6 CAPN7 GEM SERBP1 UAP1 NRK ADAMTS5 CTTNBP2NL ONECUT2 ADAMTSL3 LGR4 POM121 LBH CLPP EPHB2 LPHN2 FAM81A PLEKHA6 SESN2 SNF1LK TAOK1 RUNDC3A UBE2Q1 SEPN1 ADAMTSL1 ZKSCAN2 FGD6 CCDC71 ANKRD43 KLHL3 LPCAT1 SRRM2 NOTCH1 DTNA G6PC3 SFRP1 VAV3 CDH5 GORASP1 ATP10B ZC3H12B GOLT1B EDEM3 AMD1 TBC1D8B SLC16A1 MAL2 TMEM110 SEMA4C PARD6B STARD7 YPEL3 SEC22A KAT2B CYP1B1 ATP2B1 SAV1 ORC5L SMAD5 SLC16A10 C10ORF26 TMEM178 YPEL2 HLX DNAJC13 RYBP PSD3 PNKD AXUD1 PRR3 FNBP1 ATRN FBLN5 HAPLN1 ZADH2 TMTC2 SGPP1 PDE3B MEF2C PROSAPIP1 ITIH5L RPS6KB1 CBFA2T3 SCN3B RNGTT CDYL MYB ZNF638 |
|  |  |  |
| hsa-miR-21-5p | 1077 | B3GNT5 SMNDC1 COBLL1 MMP2 LTV1 SLMAP DLG1 PIGX BTG2 OTUD1 ATRX EIF4EBP2 CNBP PDHA1 RTKN2 RNF38 BID SOX7 HERPUD2 SLC7A6 IRAK1 CRTAM WWC2 SLC10A7 TGFB2 GAS5 LCORL AHSA2 NUFIP2 ANGPTL5 PTEN ANXA1 FRMD3 GTF2A1 MALT1 TC2N SETD2 HIC2 MATR3 TPRG1L CENPQ UGGT1 ITIH5 IGF2BP3 SERPINB5 LGR6 ERG USP47 TRIM33 HMGB3 G2E3 TMX4 SYNE2 COX15 NETO2 ARL1 ING3 EXOC5 ZNF576 RNF185 TNS3 PIKFYVE PTPDC1 SMARCD1 NFIA TCEANC2 ATAD2B ATXN10 CCL22 ARHGAP21 CCDC34 BRCA1 AKAP7 BMP3 SLK BASP1 HNMT ARHGEF7 PKD2 UTRN TIMP3 BAZ1B VPS26A PALLD FUBP3 STAG2 BBS12 ZNF704 INTS6 PHF20 CPNE4 ARHGEF12 RNF32 GTPBP1 ACAT1 TXLNG2P RBM17 INTU DCP1A SPAG11B YOD1 TAF5 CD1A KCNA1 PDCD4 C15orf52 PPP3CA FAM63B DLX2 CDR1-AS WHSC1 RAPGEF6 SLC2A13 DICER1 KIAA1715 NPPB RTF1 ZBTB38 PGRMC2 ALX1 RAPH1 LPGAT1 RBPJ WHSC1L1 sep.02 THOC2 MYO6 CORO2A PIAS3 MSH6 SOCS6 FBXO11 FRS2 ST8SIA4 FAS KIFAP3 PPP1R3D LILRB4 PM20D2 LCOR SOD3 PTK2 TRIM2 IQCH RPA2 UBR3 ABCA1 DDAH1 ACBD5 REST C8orf44-SGK3 SLC8A3 USP7 DTX3L YME1L1 SPPL3 PPAP2A MUC1 GCLM EIF1AX CPEB3 SRSF11 SKP2 BCL2 NPAL2 CAMTA1 MRAP2 MYO5B TNFRSF11B CADM2 DUSP5 HSD17B4 SLC12A6 ZNF35 KBTBD6 RNF180 PAG1 KCTD18 SOS2 MYEF2 THRB MEF2A IPP LARS SCML2 ANKRD28 EDRF1 NIPBL TBX2 RAI14 FKBP14 FGD4 DYNC1LI2 SGCB DDHD2 E2F1 BDH2 MAPRE1 LTBP1 PHF16 GPATCH2L VSNL1 GPRASP2 RAB11FIP2 KIAA0355 SUV39H2 NRIP1 MAPK10 CNTFR BNIP2 ZNF728 C1ORF128 AIM1L GLIS2 ITSN2 PTPN20 GNG12 PRRG4 FGF1 IL6R PLD1 PIP5K3 SPG20 VPS36 JAG1 GLG1 EPHA4 FANCC MARCH5 SMC1A VPS41 ULBP3 DSC2 CNOT6 ZBTB20 STK40 AGGF1 TSN SPRY2 LZTFL1 ENAH TCF4 ZNF527 GNB4 NR2C2 GPR64 NKTR SFRS3 PCSK6 RASEF RNF103 LONRF2 SPG11 FUBP1 PTGFR TIAM1 IREB2 PBX1 KIAA1012 CCDC109A FAM126B ZFP36L2 PBRM1 GANC ELOVL7 SERTAD2 KLF6 RNF2 SETD8 ACVR2A CYP4V2 PIK3C2A ABCD2 NEK7 TMEM246 ZNF805 BTK TAGAP PROSER1 PFN2 AGO4 PPP1CC ZNF667 LEPR PRPF39 MAP10 KIN FMN1 UBR5 PRPF4B C17orf75 PDAP1 NCOA3 ARFRP1 STK38L PITHD1 PITX2 ZFPM2 SEC63 CETN1 CDK6 AKIRIN1 RDX FBXO46 MORC3 ZXDA TMEM170A ANKS1B PATE2 sep.10 CHL1 SIRT2 PRKAB2 MAP3K8 AIM1 TCF21 PCBP1 MEIS1 ZGRF1 GLDN C10ORF12 ANKRD46 PDGFD BRCC3 EFNA1 SPON1 AGO2 SYT15 MTMR12 PREX2 SCRN1 SMAD7 PI15 PPP1R3B COL5A2 DNAJC16 MOBKL3 SNX13 KLK2 VIM PGM1 SAMD8 UQCRB NAA50 DLGAP1 SET EXOC8 REV3L ZRANB1 TRAPPC2 SERP1 THPO JHDM1D EDNRB GABRB2 ENO4 DSE EIF2S1 OSBPL3 SGTB PIGG COX20 NKIRAS1 STAT3 ISCU CCL20 GID4 ST3GAL6 LRRIQ3 FDX1 APAF1 STXBP5 WNT5A NEK1 NSL1 OLR1 UBE2D3 NEGR1 KIAA1551 C17ORF39 BCL7A ARMCX5-GPRASP2 REV1 RABGAP1 FGF12 GRPEL2 SOX17 STK3 HNRNPK TGFBR1 ZNF217 FOXN2 NCSTN TPM1 ACVR1C GALNT12 CRIM1 GK5 TNKS SC5D MSR1 TSNAX RASSF9 MEGF9 ANP32A MEI4 PLEKHA1 CRYBG2 GCNT2 KLF12 FKBP5 ZCCHC3 PKNOX1 RRAGC NFAT5 DCAF7 DIRAS2 FBXO3 FMOD ZSWIM6 PTPN14 ZBTB47 DMTF1 CUL3 GRM1 TNS1 SPATS2L CLIC5 ASRGL1 FASTKD2 KCNJ10 ITGB8 C1orf147 PTX3 PER2 GNG2 HSD17B7 DSG1 PTPN4 GIMAP1-GIMAP5 GXYLT2 HIPK3 CASC2 BCL6 WSB1 SOCS4 HPGD VEGFA PFKM PER3 AIF1L SYT14L ZBTB41 TGFB1 DOCK4 CCDC14 MAST4 SLC5A3 DDX46 SGO1 VPS13A SNRNP48 MYC KAT6A SAR1A SPDYA PELI1 SPTLC3 BCL11B RP2 DAG1 PLOD3 NELL2 C7 NIPAL2 CSNK1A1 PTPN9 KLF9 FIGN LEMD3 MRPL9 MAP3K1 C5orf58 RDH11 PRRC1 C14ORF101 PLAA GPD1L ZNF292 SPIN1 TNFAIP3 PSRC1 IL12A ALS2CR4 PPP1R3A DUSP10 PANK3 CNKSR2 SNTB2 WWP1 SAMD5 LRRFIP1 ZBTB8A MAN1A2 RPRD2 FBXL2 ECI2 ELOVL4 PTAR1 BRD1 PRICKLE2 CSRNP3 LIX1L MTMR9 ATF7IP CASKIN1 CCR7 SSFA2 TOPORS RHO UBE4A SMURF2 SRL FAM13A GAPT FXR1 UBE2D1 CREBL2 CREB5 TNRC6B B3GAT2 WFS1 WIBG TRIP11 PARP1 GLCCI1 FGFRL1 ASPN DDX55 BEST3 ARMCX3 ZDHHC17 PHLDB1 FERMT2 CLOCK NSUN2 PRKCE C4ORF16 DIPK2A ABCD3 NEK11 VASH2 RTN4 FASLG IRAK1BP1 FAM20B GAPVD1 PRDM11 CTR9 FAM3C IVNS1ABP GPC4 WNT1 BTBD3 UBE2NL HNRNPH1 PACRG LUM ARHGAP32 MOXD1 LANCL3 PHIP MRPS10 TRPM7 EDIL3 PHF17 ATP11B LATS2 MKNK2 MYD88 DLG2 TMEM68 RMND5A ADGRE5 SLC35F5 MARCKS ZYG11B PPARA KRIT1 SASH1 FMR1 KDM7A CCR1 DNAJA2 USP15 HECTD1 SLC5A7 SLC31A1 HS3ST3B1 STK36 SFXN1 BMPR2 B3GALNT1 CBX4 MYO9A NTRK2 FOXN3 SP100 PFKFB2 PLCB1 FAM65B CHD7 TSC1 PHF6 NUBPL CERS5 SOX5 PCGF5 KIF6 FAM136A RASGRP1 ELAVL4 CALCB PTBP3 EIF5 SPRY1 NFIB SERPINI1 PLAT CAPRIN1 LPA FGF7 KHDC1L KLHL15 NBEA AKT2 TAT EGFR LIFR CDK2AP1 SESN1 TFDP1 SP1 TNPO1 GP9 DENND1B MGA RAB6A TAF1 BOLL CLCN5 ARL4D AKAP6 MAP3K2 CEP97 JMY SLC9A6 TRAPPC8 ZNF10 MBNL1 PURA DAZL ERBB2 FANCM NIN SRPK2 CASC5 FAM46A FAM177B CFL2 RASA2 OSR1 ANKFY1 SLC22A15 PLAG1 KCNA3 CNTRL MYCBP2 LPIN2 LIMCH1 C2ORF18 C2orf43 SMARCA4 TSHZ3 EML6 LATS1 RHOB GNAQ DOCK10 SECISBP2L PDZD8 CCNG1 AKAP12 WDR7 AMBN AMH CLU SLAIN2 GPR34 GNE ATF2 SKI ADGRG2 TMEM245 MSX1 AP3M1 ZNF460 TBL1XR1 PHACTR2 ST8SIA3 KLF5 RPRD1A SACM1L APOLD1 AKAP9 TOP2A KLHL8 SLC30A10 IGF1R PPIF PURG OSBPL1A SNX30 ICAM1 RECK ABAT THBS1 ALDH1A1 PID1 KIAA1333 MTAP ARMCX1 FGF18 STRBP FANCI PTPRG NAV1 PIGN USP34 KIF27 FAM156A ATPAF1 ZNF367 PRR5L ALX4 TRIM38 DAXX CALN1 GTPBP10 DNAJB9 MRPL49 PPM1L CCT6P1 RSBN1 RAB6D NOP14 RALGPS2 NTF3 EPM2A SNRK PDHA2 MXD3 MON2 RASGRP3 TNFRSF10B SUZ12 EXTL2 CCL1 BTN3A3 HIF1A MED21 MCMDC2 ELF2 WNK3 FBXL17 PDZD2 MPP5 RNF111 SLC26A2 ATP2B4 C8orf17 IST1 RAB22A GDF5 RB1 MINDY2 BAHD1 SERAC1 HOXA9 RHOQ PAN3 REPS1 MSH2 ERP44 RIT1 SETD1B MED9 MAP3K7IP3 MTPN GPAM SETD9 ESYT2 MGAT4A ZNF99 TMED10 CASTOR1 PHF14 S100A10 MBNL3 NAA30 KLHDC5 SOX6 ZFYVE16 CLIP4 PAIP2B RAD51AP1 E2F2 ARID4A MBLAC2 PLEKHA2 PHTF1 FILIP1L TLR4 RAB6C EIF2C2 TMEM163 CXCL10 IPO11 MSTN ZNF532 ACVR2B VCL COL4A1 HMGCLL1 SEZ6L RSPRY1 ADAMTS3 SMC5 ZNF587 SMN1 XKR6 VPS54 BRMS1L GLS BACH1 DOCK5 RBMS3 PRMT9 TM9SF3 DCAF10 TGIF1 TP53 ROBO2 CDH7 CREBRF MATN2 RSAD2 RAD51L3 TGFBR2 PLEKHA8 PURB CHIC1 ACAP2 NT5C2 UNC80 ZNF680 TGFBR3 ZMYM2 EHD1 RASAL2 GTF2I CDKN1A SLC2A4RG PHF20L1 LAMP2 RAB11A KDM1B SOWAHC RPS7 CNOT8 RASA1 APH1B GABRG2 CXCL5 GPD2 CDK19 BTBD7 ITCH CATSPERE KLHL42 TOR1AIP2 NCOA2 C20orf194 FAXDC2 PIK3R1 NRXN3 PTPN3 RASSF8 FAM217B EIF4A2 TET1 DMD FOXP1 PREPL CLDN8 CD47 SCN8A SPRY4 GIMAP5 SOCS5 RSRC2 BOC CUX1 YAP1 SC5DL CCDC121 RAD21 CKAP5 RUFY3 MYCL1 KCNMB2 NFKB1 HPS5 ATRNL1 MIA3 PCBP2 IL1B HAX1 CEP152 SREK1 GP5 ASF1A CFAP300 TUBGCP5 SEMA5A INPP4A ADNP RBM27 PTPRU RFFL AFTPH CALD1 NIPAL1 ST6GAL1 BCL11A MKX CLTRN SAMD9 S100A7A DNM1L TESK2 ORC4 DDR2 KCTD12 ZNF326 ZNF207 MPRIP ACTA2 DERL1 GRAMD2B ARHGAP24 JPH1 ZNF662 RIPOR2 TMEM2 ALMS1 LAMA4 ZNF592 LRRC57 MOAP1 RNF11 PPFIA4 CERS6 CLIC2 BBIP1 N4BP2L1 NXF5 LPIN1 RSF1 ETNK1 CD69 FOXO3 LIN7C TRIM59 PLP1 APC TLR2 OLFM3 BCAT1 WNK1 DOCK7 C9ORF100 GATAD2B SGK3 LYRM7 FBXO28 BNC2 ANXA4 CA1 TTC33 CYBRD1 RGS7BP PARP9 RETSAT AUTS2 POM121 STRN GAB1 KIAA1310 TGFBI UBE2D2 DDX3X DEPDC4 SCAF11 HNRNPU TENT5A MAP2K3 DYNLT3 TMEM56 SESTD1 E2F3 NCAPG EPM2AIP1 KLHL3 RASSF6 CHCHD4 APPL1 GOLGA4 DAAM1 FBXL13 FCHO2 MIB1 SLC17A5 TMEM147 HGF KHDC1 SATB1 ATMIN RNF6 GRAMD3 TP53BP2 SOX2 TP63 GLS2 COL12A1 LMBR1 VHL EIF2C4 AMER1 PDLIM5 RPS6KA3 KIAA0825 BRWD1 ZNF280D SLC16A10 LANCL1 KBTBD7 MDM4 AP1AR ODZ4 ZNF200 FNBP1 HAPLN1 ZADH2 KLHL24 DUSP8 ACTR2 MEF2C MMP9 CDIP1 POLR3B SPEF2 DCAF8 KLF3 CDC25A ANKRD37 CTSC |
| hsa-miR-103a-3p | 1277 | MMS19 TBC1D19 COBLL1 CHPT1 FEZF1 FAM219A ERBB4 AMER2 KCNJ2 GSKIP MAP4 ZHX3 BTG2 ITGA5 CNTNAP1 RTKN2 VPS4A MEF2D VAMP8 RNF38 PVRL1 SALL1 MACF1 WWC2 RAB1B MED20 NUFIP2 MBNL2 PELI2 PTEN DLEU7 SIDT1 SCAF8 AGFG1 EPB41L4B MITF ATXN7L1 HIC2 UGGT1 ZNF85 POU4F2 TAF9B BTLA SERPINB5 FNBP1L CACNA2D1 MTM1 SH3BP4 CDK14 SLC25A37 USP47 FAT4 SMARCC2 ZBTB34 ARL2 GNAI3 COX15 TOB2 FAM20A ADAM7 MAP3K3 GSTCD SSH2 EXOC5 COPS7A IRF2BP2 UNC5A RAB40B PROSC TNS3 IGSF3 ARF6 IPO9 PI4KB TEAD1 NFIA CHD1 RC3H1 STRADB CCDC50 LAPTM4A ARHGAP17 KIAA2022 RAB3GAP1 GFRA4 TAOK3 HERC2 KIAA1033 EXT2 C2orf91 ZNF506 ABL2 MECP2 C19ORF6 NOS1 CDC14A ACSBG2 TMEM86A USP6NL ROCK2 SNX16 ARL11 PHF20 EED RNF32 COL1A1 GLUD2 ATL2 AP1S2 MAN2A2 NT5M DACH1 KIAA0528 KCND1 DCP1A HISPPD1 KPNA3 TAF5 CEP350 TBC1D12 FAM73A KRTAP10-4 DBNDD2 C1QL3 WHSC1 SLC2A13 NEIL1 FAM192A ZDHHC16 GALNT13 MRPL19 FAM98A DCBLD2 RBM33 PPIL4 CACNA1E DICER1 SYT10 PCDHA13 KIAA2018 PGRMC2 GGA3 PCDHA4 CCNE1 RP5-1022P6.2 SSU72 CAPZA2 LRRC8D sep.02 THOC2 ATP11A PCMT1 VAMP1 RBM24 GRB7 SOCS6 AKIRIN2 RIBC1 ARMC1 CDK8 GLRA2 ARNT BDNF LCOR SLC15A4 USP49 TRIM2 BTRC RNF217 UBR3 DESI1 C18ORF34 GCC2 OTUD4 TNFSF10 REST BCLAF3 YWHAQ CHEK1 RFX3 KRTAP11-1 MBOAT1 EIF1AX CPEB3 BCL2 MMP16 SLC2A3 JAKMIP2 ATP13A3 ACOX1 ABHD2 USP24 KIF5B TRAM1 TWF1 DUSP5 PSMD3 CSDE1 MYH9 ZNF41 FAM110C SLC12A6 SFRS16 KIF18B FAM133B C12orf76 PAG1 PRPS2 CHST11 DCLK1 SNF1LK2 AQP2 ALAD USP42 GPCPD1 RGS4 PPP2R5A TRAF3 ADAM23 USP10 DHX33 CDC42EP3 MFN2 RAI14 CHD6 ESRRA FUCA2 C11ORF68 DYNC1LI2 DDHD2 PIK3AP1 MAPRE1 CDK17 MOBKL2B GATAD2A APPBP2 PRKD3 RAB11FIP2 INO80D NRIP1 POF1B NRP2 RASSF5 CACNB1 TMEM87B NACC2 QKI TMEM33 AHCYL2 IZUMO2 ARIH1 BCL2L2 DAPP1 SIPA1L2 ARL4C ATF7 ABCF2 UBQLNL RGPD5 LASS6 MFAP5 WDR20 CHRM1 PLD1 ARMC8 MYNN DCUN1D3 CORO2B PRDM4 C2ORF71 TTLL11 CNOT6 DLL1 MYO10 HLF SMYD5 SPRY2 OLFM1 RET RUNX1 GMFB ENAH C16ORF72 HSPA4L NR2C2 FAM54B NKTR SMIM10L1 TSHZ2 FRYL ESRRG SLC12A2 PALM2-AKAP2 OTX1 DNAJC3 EPB41L1 BST1 UBE2W FOXJ2 GPR12 ASH1L ZNF711 UHMK1 FAM171A1 NAA16 VAMP4 PPP4R3B MLIP BCAP29 PLAGL1 PIM1 PSMF1 DYRK2 ARIH2 SH3PXD2A MTF1 SH2D2A C5orf47 KLF4 ACVR2A PGM2L1 ANKRD52 DLX6 SCN1A FSTL4 RPS6KA6 ASPH AGO4 PPP1CC FAM189B WNT3A FLOT2 TFEC TMEM25 SCN3A NUAK1 DCX PLSCR4 PITPNA FMN1 STRIP1 CDC37L1 PRPF4B NRBP1 RALGPS1 CCDC6 KLHL6 EYA1 PRR14L ENTPD7 ZFPM2 CAMKK1 RPUSD1 SLC9A7 OAS3 SATB2 CDK6 YWHAH C6ORF201 LCN15 GABRB1 SMPD1 ITSN1 TMEM170A SVEP1 MRRF PATE2 LIN28B TMEM47 TNF PRKAB2 RAPGEF1 AFF4 DLG4 STC1 TTLL7 TMEM248 ZIC1 NAA20 STOM FURIN AMMECR1 JPH2 DLST HDGFL3 INSL5 ZYX ANK2 PAWR TLK1 GTF3C5 RIMS3 COPS2 EXOC3L2 FAM160B1 SLC1A2 SGK1 GPATCH8 KIAA0930 PRMT8 MOBKL3 RAB9B CPD SNRPC FGFR2 RCAN1 TMEM35A BAI1 FBXL20 DNAJB4 KIF21A THY1 PDPR PHKA1 CSNK1G2 SYDE2 EPHA7 JHDM1D LMAN2L MAP7 ASIC1 OSBPL3 LGR5 AKAP2 SPDYE3 SRGAP2 PPP2R5C SUSD6 LRP1B MCFD2 XPO4 DDX52 STXBP5 DNAJA1 AQP11 SPDL1 KALRN TRABD2B MPP2 GNS WIPI2 IFNAR1 NAA60 ITPR1 RAB11FIP4 NSL1 KIF5A NF1 SLC44A1 WBP11 SPRED1 LOC100130357 PFTK1 GALC NAV2 TBKBP1 PAK3 UBE2J1 ZNF217 TGM3 TGIF2 ACCN2 MARCH3 TPM1 DPH6 PDS5B SETD5 EBF1 RUNX1T1 LRRC61 SLC35G1 NOTCH2 SFRS2IP ATP1B2 ADD1 AP2B1 RS1 TECPR2 UBE2Q2 PLEKHA1 CRYBG2 ESR1 TRPC1 ANGPTL8 ZCCHC3 SMS RRAGC NFAT5 SCAMP5 NUDCD2 LRRC15 OSTM1 CXORF23 DVL1 GSTT2 ABCB5 IRS2 AHSA2P CCNYL1 GALNT7 TNFAIP8L1 SIK2 PCGF2 DMTF1 GRM1 PDE4D IL1RAP SPATS2L CNTN5 FAM122B TJP1 TSPYL5 ZKSCAN1 PRKAG3 NR1D2 ERC2 BSDC1 PTPRT ARFGAP2 TMED2 PTPN4 TBP VEGFA STAT6 PPP6R2 ZNRF2 ELL ENPP2 sep.05 PER3 TRIAP1 ARHGAP19 PPM1E ITGA2 GLI3 GRIN2A KANK4 TMEM20 GREM1 AK2 SUN2 OSBPL6 NAA15 NR4A3 FRMD4A MIGA1 ZNF223 GOPC CCNT2 ZC3H12C BHLHB3 BCL2L13 IL10RB PLS3 GPR124 JOSD1 ZBTB39 NUP50 POU3F2 CSNK1A1 PLEKHF2 KCNIP1 E2F7 RNF138 VCP MFSD14B RDH11 SRSF3 HSDL1 C14ORF105 C14ORF101 KIF1B SPIN1 GDPD1 ARL8A C15ORF57 KIF5C LIN28 PDK4 TSPAN5 sep.08 SNTB2 WDR82 KPNA1 PDIK1L NEK10 FAM104A FAF2 WWP1 FSD1 LAMC1 M6PR GRAMD2A PDCD10 ZNF90 CAMK2G SSBP2 WNT16 TK2 KIF3B EFCAB4A RAN LPHN3 GTPBP2 SEMA6A ELK4 KANSL1L HMGA2 ETF1 WEE1 MMP24 ADCY1 UBE4A ACER3 RNF165 AVL9 CASP3 TNRC6B PIGB EIF2C1 SEL1L CC2D1B HSD17B13 AMTN YWHAB DGKK ZNF461 KIF23 SIAH1 FAM60A FGFRL1 COPS7B EIF4B TMSB4Y DCAF12L2 FERMT2 CLOCK C2CD5 LOC26010 AGO1 THAP2 KATNBL1 TPD52 PRKCE ARHGAP20 GREM2 KIAA1045 PHF5A NPAS2 MYBL1 CNTN3 CACNA1C IHH C6ORF89 WASHC4 PRDM11 XKRY AMPH C1ORF21 KDR RGPD4 SOGA1 GLUD1 FOSL2 ST3GAL2 OGT SLC23A1 KIAA0831 SFRS2 ARHGAP32 SYNDIG1 KPNA4 WDR61 EPHA3 ODF2 NECTIN1 RBM12 D4S234E FEM1C EDIL3 B3GALT4 CARM1 C5ORF41 ANKRD57 LATS2 GADD45G ZC3H7B HTR4 DSC3 ZIC5 BTN1A1 SACS C13ORF1 SAP130 AFF2 PPARA NRN1 PLD2 IFIT1B SLC2A14 DUSP28 MN1 RORA ATP6V1B2 KIF2B ARHGAP18 PPP3CB KIAA0247 KDM7A UNC119B LRRC55 MAP3K21 BACE1 TBCEL GFOD1 SAPS2 KLF7 BLMH ADCYAP1 NTRK2 GCC1 GDAP2 YAF2 MAP3K7IP2 SH3TC2 PXMP4 MAP2K5 SLC36A1 PLCB1 UBE2R2 CPEB2 SDCBP SAG PDLIM1 G3BP2 ISLR CD80 HNRNPA2B1 TSC1 SYT6 LRP1 PCGF5 PRKG1 SLC8A2 FAM136A TACC1 SBF2 PPP3R1 CCNJ EIF5 IGSF23 UBL3 NFIB CAPRIN1 SNX18 COL4A4 FGF7 YRDC THSD7A RPGRIP1L SEC24B AKT2 PACSIN1 RASL12 PABPC1L ZDHHC3 CYP3A7 SESN1 AXIN2 C20ORF141 TNPO1 GPAT4 DENND1B DMBT1 RASGEF1B MYLK PLGLB2 PGAP1 HOXD10 CLCN5 ZDHHC9 SH3BP5 SEC22C PPME1 TRAPPC1 CHD2 MTMR4 HTT MBNL1 USF3 RAB40AL MCM7 TBPL1 DAZL DCUN1D4 TRAPPC11 NIN SLC1A4 TCAF2 POLDIP2 SPG21 PLA2G15 STAG1 CALU TDG ANKFY1 IQGAP1 ABHD13 PLAG1 EDA ENTPD4 CFL1 SCN2A CD28 ALDH8A1 BAZ2B WNT7A XPO1 TAGLN3 ZNRF3 ENSA ZNF449 GNPNAT1 NISCH BAZ2A PDZD8 RPL34 HOXC10 HTATIP2 TTL FGF2 KCNG3 PHYHIPL SLAIN2 HMG20A CEP85L RPA1 GLIS3 CCNT1 IARS MPP6 TBL1XR1 PHACTR2 C20ORF39 TMEM71 ST8SIA3 ZNF880 SINHCAF ADGRL3 TMEM221 MAPK8 ESS2 NELF MICAL2 CPNE3 BCAT2 ZNF589 XKRY2 EFNB2 SIX4 PID1 TAL1 NLGN1 ACSL1 RNASEL C6ORF204 TMEM260 SLC20A2 NAV1 MCHR2 RAMAC ANKRD44 SRGAP1 SHROOM3 MC2R MKL2 MSI2 TMCC1 THBS2 GRID2 CA2 GTPBP10 LRP2 ANK1 PAFAH1B2 SEMA6D TARBP2 IKZF4 SLC25A20 HSPE1 MYCN RSBN1 ANKRD29 APLN SNRK GPN1 SGMS2 CRELD1 SNCG GPR6 UBE4B SDHAF4 SUZ12 CDK5R1 TRIM71 SLC45A4 SCAMP1 WNK3 SEMA3D ZBTB10 C1QB SPTBN2 WDR22 BHLHE40 SLC26A2 CAMK4 PTH MAPKSP1 KIAA0895 ARHGAP5 ATXN1L SNX3 MINDY2 VSX1 HYKK CEACAM7 SPRY3 DYNC1I1 SLC35D1 FAT3 GRIK3 ERLIN1 EN2 CAB39 CCDC85C TMEM200B NCKIPSD FBXW7 CASR CLCN3 ZNF606 MAP3K7IP3 FBXW11 DKK1 MLLT10 PHF15 CSNK1G1 NRIP3 GABRA4 MBNL3 NAA30 SALL4 PCDH17 TRIM10 LRRN3 SOX6 ZFYVE16 PTPLB CDKN1C CBARA1 TSPAN9 HSPH1 ZSCAN31 SGCD ARID4A THUMPD1 GFAP DPP8 OTUD7B KCNC4 RAB30 C5orf60 KTELC1 GOSR2 IL16 HAUS5 VCL ACVR2B TCF3 IYD SUFU ISOC1 VCAN UBE2A SMC5 ZDHHC21 PANK2 CECR6 GLS STS C3ORF23 DSEL PEX5 MLH3 TMEM194A CSNK1G3 C2ORF42 PPP6C PLGLB1 ENTPD1 PDCD11 TM7SF3 NHSL1 OSCP1 TGFBR2 ORAI2 ZCCHC2 PURB CHIC1 SYPL1 CARNMT1 SLN PTCHD3 UNC80 SLC39A10 TGFBR3 SLITRK1 CDC23 ZMYM2 UMOD MAP3K7 SLC35A3 HERC4 MTSS1 MIPOL1 PPIP5K2 ARHGAP6 CDKN1A KCTD8 SYNJ1 MMD ANKRD53 PTPRM SH2D4B PDS5A CD8B ATXN1 NEDD9 PLEKHS1 SOWAHC GORASP2 JAK1 MYRIP GABRG2 C5ORF24 EVA1A CTNND1 CAMKV PATJ CDV3 N4BP1 PPP2R3C CELSR2 CUL4A PIK3R1 RASSF8 ZBED2 TMEM55A FAM217B HELZ RNF125 ANKIB1 TARP FOXP1 STAMBPL1 CISD1 KIAA1804 NEXMIF SCN8A HSD17B12 NOVA1 RAP2C DPY19L4 ASXL1 SLC9A8 HS6ST3 FZD6 DLG5 YAP1 CDC42SE2 SNX12 RNMT TTC17 MASP1 CD164L2 ELP1 MYCL1 ZHX1 MAP2K1 ICOS ZNF654 ANK3 BOLA3 AREL1 ST13 TMEM135 SPATA13 MED26 ZNF736 DUS1L JUB CLIP1 NSG1 RGPD8 NHLH1 SREK1 PDE8B UBFD1 SEMA5A MIF4GD ARPIN BMX UBE2V1 TLL1 TMEM132B ST6GAL1 C5ORF13 UNC84B sep.06 BCL11A NARG1 RAB10 SV2C TLE4 C2ORF69 CCDC198 SH3GL2 RSPO3 ANO3 ZNF326 KLF13 FAM117B TAF15 FBXL18 UPF2 ZNF662 ZNF592 RNF157 DCN CNIH3 PAQR9 POLD3 UBE3C MYST2 SLC13A3 RNF13 ETNK1 DCTN5 ZBTB5 LONRF1 TRIM59 FAM131B PSME3 PLP1 PSEN1 CNNM2 ZSWIM3 FAM70A GPC6 INADL KCNMB4 HIP1 SBNO1 ANKMY2 WNK1 LIN28A LARP4B ETV6 PDK3 SEC24A RELN TPR C18ORF25 GATAD2B SPTBN4 AKAP11 TRPS1 HMGA1 SERBP1 ZSCAN22 AMOT STAG3L4 YTHDC1 PCNXL2 USP28 TTC9 C15ORF41 STK38 SLC26A7 BACH2 ADAMTSL3 TBC1D9 PRRT2 STRN ROR1 PNN ESPN EPHB2 FAM81A ACTG1 MTMR3 KCNN4 DEPDC4 CRKL KDSR SCAF11 DYNLT3 GAD1 ITPRIP SLC4A4 UBE2Q1 GIT2 E2F3 RFC5 CRYBG1 APPL1 HACD2 MTFR1L DPP10 AAK1 DTNA C20ORF29 AKT3 RALBP1 LRRC14 VAV3 UBR4 POLA2 ZC3H12B KAT7 EDEM3 NPAS3 GATC MIB1 RBAK NDEL1 ATG14 SMARCE1 PISD CLIC6 MBD1 ACSL4 WAPAL SMEK2 FAM176A ARL8B DPYSL3 RGPD6 FBXO10 ARMCX2 GLS2 VDAC2 SAV1 EIF2C4 ATF7IP2 RPS6KA3 BRWD1 SMAD5 C15orf40 PCDHA6 FCHSD1 PNPLA2 MDM4 RYBP MAMLD1 S1PR3 HCLS1 ACTR2 CNOT6L TRIM68 PDE3B RPS6KB1 DRD1 RNGTT MYB CDC25A APBA1 |
| hsa-miR-30c-5p | 1149 | B3GNT5 PKNOX2 ITGA5 LPP HIVEP1 RTKN2 MMGT1 SLC5A11 RNF122 MEF2D ANKRA2 RCBTB1 SLC7A6 FAM178A NAGPA ADAM12 ITPK1 LCORL UBE2G1 GALNT3 NUFIP2 TOMM20 CTNND2 MBNL2 UBAC1 TAF4B EPB41L4B MEX3B HIC2 MATR3 TUBGCP3 POU4F2 TIA1 ERG USP47 HMGB3 FAM109A C7ORF60 ZBTB34 KLF11 CHD9 CCDC97 PAPOLA EPN2 GDA SLC16A14 DPYSL2 SSH2 SOX4 HOXA1 OSBPL8 NR4A2 MFSD6 ALPK3 RWDD4A PTPDC1 EPDR1 CXORF39 SCYL3 NRP1 SNX33 CHD1 CDH13 SLC25A14 GJA1 ATAD2B KIAA1949 GNAI2 FAHD1 ELOVL5 ARL4A HERC2 KIAA1033 ABL2 CLN8 ASCC3 MECP2 PCGF3 BSN MAP3K5 STX2 TIMP3 FUBP3 SNX16 STAG2 SDAD1 ENOX2 ZIC2 PHF20 EED PARP16 CECR2 SYNGR3 YOD1 EML1 C1ORF135 CTDSPL2 KPNA3 CEP350 TRDN TBX5 UBE2F PPP3CA PLCG1 MLL GFPT2 TSGA14 RTN4R BCLAF1 NEDD4 C21ORF66 NEK4 RAD23B PPIL4 YTHDF3 NCOR2 ZNF148 UBE2I CCNY RP5-1022P6.2 RAPH1 LPGAT1 CNTN4 LRRC8D GRAMD2 CHST1 CEP170 GAN HECTD2 VAT1L HDAC5 SOCS6 FRS2 ST8SIA4 LMLN SLC6A15 REEP3 MARK1 BDNF CCDC43 SEMA3A GNPDA1 DDAH1 OTUD4 EDC3 PLXNA1 MFSD11 MIER3 MEIS2 CPEB3 BCL2 CAMTA1 JAKMIP2 ARID3A WDR43 STOML1 ABHD2 SON SLC6A6 TWF1 CHFR ARF4 MYH9 LRRC17 ZPBP2 PPP1R9A TNXB FAM152A OTUD6B SLC12A6 ADAM19 MYEF2 ADRA2A NEFM BCL9 ADAMTS9 SEC23IP TASP1 MAGI3 EDNRA FAM40A SNX6 ZBTB11 CAMK2D MARCH8 CNGB3 IRS1 SGK269 RAI14 PIP4K2B SGCB PHF16 ZMYND8 RGL1 DLGAP4 KIF16B ACTC1 KIAA0355 INO80D NRIP1 SUV39H2 NRP2 QKI C13ORF18 NCAM1 ACP2 PALM2 ARID5B FREQ TMEM33 KRAS CUGBP2 ARL4C ACTR1A GALR1 FUT4 CSDA DCUN1D3 DHX40 MARCH5 ARL6IP6 EVI5 UBXN7 RARB CNOT6 PICALM CXCL11 PRRG1 MYBL2 HLF HEPHL1 RUNX1 ASB3 CARS SNX10 NAALADL2 NUPL1 DBF4 MTTP KCTD5 PTPN2 ESRRG PPTC7 HDAC9 GRHL2 AHNAK STOX2 FOXJ2 CNR1 PBRM1 ZNF711 PPP1R12A SSR3 CHMP2B ZCCHC24 DGKD GOLGA1 SORBS2 VAPA SH3PXD2A NR3C1 PGM2L1 YWHAZ SCN1A GNAO1 PFN2 ZC3H6 GOT2 RRAS2 LEPR AASDHPPT RAB38 ARPC5 SCN3A NUAK1 DCX PPARGC1A RRAD CDC37L1 NRBP1 RALGPS1 CCDC6 SNAI1 CCDC120 SOX9 CBLB CUL4B SLC35F1 SATB2 PITX1 LARGE PDGFRB ADRB1 MLL3 CELSR3 AP2A1 GABRB1 HOXB8 ITSN1 LIN28B TMEM47 TLL2 CHL1 FLJ45983 AFF4 DIXDC1 SUB1 AKAP10 STC1 ASAP1 TTLL7 PDSS1 LYST SOX12 C15ORF29 GRHL1 ARL10 SMARCA5 NEDD4L ATP2B2 NHLH2 FRMD6 IL1A PLS1 DGKZ TCP11L1 YPEL5 PAWR RAB23 C14ORF129 ELAVL2 SLC1A2 RIMBP2 FAM160B1 ZNF649 DNAJC16 RNF7 PGM1 GOLGA8A CADPS CPD CSMD3 KLHL28 CLDND1 TANK RCAN1 FBXL20 ARHGEF3 REV3L B4GALT6 ATG5 DOLPP1 SUPT3H EDNRB RHEBL1 SPAG9 ZNF518A TTBK1 GM2A SLC38A2 C1ORF174 CD2AP RAB15 MYH10 ICA1L ADAM9 ZSCAN29 BDP1 MYH11 UBA3 EBF3 NSL1 EIF5A2 NF1 LPPR4 UBE2D3 NEGR1 FAM49A PLEKHA7 MYO5A STX7 NEFL C4ORF34 SPEN REV1 GALNT2 ADO OXR1 ANKRD17 UBE2J1 TSPAN33 ELF1 CCNK DNMT3A LHX8 NT5E COL19A1 PDS5B SETD5 ITGA6 NCK2 ZNF644 EAF1 TNKS LRFN2 SNX2 HNF4G ABI3BP TULP4 MEGF9 ZBTB7A EIF2C3 SLC30A4 TOX KLF12 GCNT2 PGGT1B PLEKHO2 NFAT5 NEUROD1 GIGYF2 CALB2 TMOD2 PLCXD3 USP45 IRS2 JPH4 ALG9 GALNT7 KIAA2026 BTBD10 HERC3 MXRA5 RND3 CYYR1 PDE4D IL1RAP ITK CCNJL TXNDC5 RALGDS PER2 PAPD4 TMED2 MAP2K4 RASGEF1A PRUNE2 ADRB2 TRIM9 BCL6 SLC4A7 ELL MFAP3L PIK3CD SLC7A11 PITPNM2 ZBTB41 PPM1E DOCK4 VAT1 COL25A1 SLC7A10 SLC5A3 CAMK2N1 DDX46 CHST2 WDR26 BCOR FRMD4A PELI1 BCL11B NTRK3 CCNT2 SOCS3 GLCE DAG1 SLC35F3 GPR124 JOSD1 ZBTB39 ELMOD2 CSNK1A1 ARRDC4 KLF9 FIGN PYROXD1 CHKA TSPAN2 E2F7 RNF138 YES1 RIPK5 FAM91A1 SP4 RAB4B GNA13 EGR3 AMOTL2 SEH1L KCTD9 RAPGEF4 LIN28 sep.08 ZBTB6 WDR82 C8ORF4 FAF2 FAM104A ZRANB2 WWP1 CLTC IHPK3 ZNF521 GCLC CBX2 MAN1A2 PDCD10 RAP2B COL13A1 USP37 SSBP2 ADAMTS6 PTAR1 BRD1 C19ORF50 FAM123B CDC7 SNAPIN LPHN3 REEP1 DLL4 SETD3 SEMA6A GMEB2 GPR110 STK39 ADCY1 NR2F2 SPAST FHOD3 HNRNPC PRDM1 PSTPIP2 RPS6KA2 SMAP1 RNF165 ZCCHC14 FXR1 UBE2D1 AVL9 TGDS ICK CASP3 TNRC6B FOXG1 BNC1 EIF2C1 ZNF608 SLC22A5 GLCCI1 SYN2 COPS7B C9ORF72 GZF1 WDR47 ZFX ZDHHC17 XPR1 ABCC9 PRPF40A SEC23A CLOCK YBX1 IRF4 CPNE8 MCF2L JAG2 DACH2 GLT8D3 RGS2 NPAS2 NR3C2 PTPRK VPS26B EMCN CACNA1C FOXP4 DOC2A FAM73B DCTN4 UHRF1BP1 PPP1R2 IL1RAPL2 GRB10 GRM3 TBL1X EPHA3 P4HA2 TRPM7 RBM12 D4S234E EPB41 INSIG2 PRKRIR FBXO34 RNF220 RCOR1 IKZF2 SMAD2 ODZ3 CBFB HTR4 MARCKS PAPD5 SACS C20ORF108 PLXNA2 ANO4 PTPRD IL2RA PIK3C2B SLC22A23 FAP CAPZA1 CCDC117 SCARA5 PRG4 PPP3CB KIAA0247 DDIT4 USP15 NUS1 GTF2H1 PTGER2 UBN1 A2BP1 VKORC1L1 STX17 LYN BCL2L11 USP6 AFAP1L2 NAP1L3 SFXN1 CNTD1 YAF2 MIER2 SLC36A1 IRX4 CPEB2 PAK7 CHD7 SLC30A8 CUL5 SNX27 TSC1 PHF6 TNRC4 SBF1 PCGF5 RAP1B TNRC6A BIRC6 SMARCD2 DGKI RCHY1 TACC1 RAD9B ADRA2B PPP3R1 EPC2 DMXL2 PDCL STIM2 NFIB MBD6 DIP2B SNX18 NLK PCDH20 PIGA ZNF238 RASL12 KREMEN1 LRCH2 TRIM13 MAML1 LIFR TFDP1 PIP4K2A TNPO1 DENND1B ZNF706 SLC35D3 UNC5C PGAP1 MAGI2 RNF146 MAP3K2 PCDH19 NECAP1 LEPROTL1 TBPL1 ZFC3H1 CSGALNACT1 STX12 C14ORF28 CREB1 SMAD1 FAM46A CFL2 PTPN21 STAG1 CALU TDG PLAG1 CCPG1 SORCS3 HSPA5 PDGFA LIMCH1 ANKHD1 JUNB PDP2 SCN2A KIAA1370 TP53INP1 C12ORF30 BAZ2B XPO1 CPSF6 RHOB GCOM1 GBP1 SAP30 DOCK10 MARCH6 WDR7 MEOX2 GRM5 EML4 PEX3 FKBP3 TUSC3 FNDC3A AP1B1 CA10 AP3M1 CEP76 LIPG PHACTR2 UBE2O RPRD1A LGI1 PDE5A NR5A2 NECAB1 IGF1R KCNJ12 CPNE3 SNX30 ATP8A1 CPEB4 ACVR1 FAM179B ESCO1 SIX4 NLGN1 ABL1 PIK3R2 NAV1 JARID2 VIP CCNE2 CALCR PLAGL2 PCDH10 MKL2 MSI2 TMCC1 LRRC40 THBS2 ELMO1 DIO2 LYCAT MTDH MAP3K12 DCP2 SEMA6D LMBR1L IER5 KCNJ3 CMTM4 POLR1D RBM5 WDR44 NID2 VAMP3 GLDC SH2B3 FRK C22ORF28 EXTL2 CDC2L6 FUCA1 ALS2CR8 RASD1 SOCS1 SCAMP1 C13ORF23 FBXL17 SLC41A2 RAB32 CAMK4 RAB22A PRLR STAU1 AP3S1 ZFYVE26 FGF5 FAM43A ZNF280B BAHD1 LYSMD3 GAS2 ERLIN1 CACNB2 METAP2 MSH2 ZEB2 SHOC2 NTNG1 MTA1 SBK1 MAP3K7IP3 TFAP2A PGR RUNX2 INTS2 STK35 ZNF507 USP48 CAPN5 TMED10 PHF15 CSNK1G1 MBNL3 TMEM121 C7ORF43 SAP30BP PCDH17 RFX6 TMEM87A ARID4A FAM83F ZSWIM5 NADK PCDH9 MCF2 DET1 KTELC1 MKRN3 P4HA1 MAN1B1 GPR85 SLC30A5 AZIN1 SFRS7 SERPINE1 ADAMTS3 ZDHHC21 NKAIN2 RARG RANBP10 CECR6 BACH1 NHS STS GLI2 DSEL ZFAND5 RAPGEF2 MAFG MNT KIAA1024 ARHGEF6 CBX3 LRRFIP2 ZCCHC2 RGS6 CHIC1 SRGAP3 ACAP2 SYPL1 TBC1D10B NCALD PHF13 KIF3A SLC39A10 KIAA1211 PTGFRN CIT AMOTL1 MTSS1 DAGLA KCTD8 NEUROD6 MMD SAPS3 ORC2L KCTD7 C10ORF76 CAT RAB11A ZNF746 TMEM30A CUL2 MIS12 PDS5A SNX1 UNC5D ATXN1 CTRL MARCH4 C3ORF57 RASA1 TBC1D15 JAK1 SLC39A11 SETD7 BTBD7 VANGL1 HNRNPA3 KIAA1328 JMJD1A S100PBP BNIP3L GABRA5 LCP1 NRXN3 MAF STXBP1 FRMPD1 TET1 DMD FRZB SLC6A9 TMEFF1 GPR125 KIAA1804 KLHL20 STAC SCN8A NFYB NOVA1 ZFHX3 FZD3 RAP2C EFNA3 ASXL1 IDH1 CAMKK2 SLC9A8 DLG5 CFDP1 PAX3 FBXO45 PON2 PSMD7 C9ORF86 BMI1 SSX2IP SH3RF1 FNDC3B RNMT DLGAP2 MICAL1 KCNMB2 ARL15 SIRT1 ATRNL1 C2ORF55 MIA3 SOX11 SPHKAP ZBTB44 TMEM135 PTP4A1 ITGB3 FAM46C CPE WIPF1 IGF2R PIP5K1B RNF8 UST RFFL CALD1 RBM15B PPP1R14C FOXD1 C5ORF13 FLJ36031 HOXA11 BCL11A sep.06 KITLG RAB10 ARID4B SOBP ELL2 SH3GL2 IL28RA ANO3 KLF13 UBE2V2 PPP2R5E BECN1 GPT2 ADAM22 MAP4K4 ROD1 SUCLG2 UCP3 UBE3C DEXI ACTN1 IFNAR2 R3HDM1 KPNB1 PRICKLE1 NRBF2 SEMA6B RSF1 FOXO3 KLF10 KCTD3 LIN7C JDP2 ERRFI1 NKX2-2 PSME3 FAM131B sep.07 MGEA5 GALNT1 DOCK7 STX6 RFX7 SEC24A B4GALT5 MFHAS1 SGK3 TRPS1 KPNA6 BNC2 ZSCAN22 YTHDC1 NRK ADAMTS5 ATP2A2 ONECUT2 BACH2 FAM110B ME1 PRRT2 PNN ZFY ESPN EPHB2 PPARGC1B UBE2D2 FAM81A AR SNAI2 CALM1 CRKL GATM TAOK1 AP4E1 PLXDC1 KIF3C DYNLT3 EPB41L5 OMG SLC38A7 CLCF1 E2F3 PHTF2 TADA2B FGD6 ABHD6 USP44 GOLGA4 NOTCH1 PTPN13 PPP4R4 EEA1 VAV3 C13ORF15 GIGYF1 CYP24A1 MAB21L1 EDEM3 MIB1 FAM13A1 NDEL1 HIPK1 ATP8B1 MAT2A PAPOLB URM1 ARID1A RNF44 ARL8B SATB1 IL7 SCN9A FLVCR2 ZBTB40 DPY19L1 GRIA2 COL12A1 ATP2B1 LMBR1 EIF2C4 BRWD1 MAST3 C10ORF26 YPEL2 DACT1 CAMK2N2 SLC6A3 HLX ELAVL3 DNAJC13 ZNRF1 PSD3 PNKD EYA2 ATRN KLHL24 HECW1 RAB7A C4ORF19 GOLGA8B RAB8A AFF3 RNF169 |

**Table S7.** DAPs from a project using the the Tromsø study. 35 DAPs were identified.

| Number of genes | Gene symbols |
| --- | --- |
| 35 | TTR PROZ PARK7 PFN1 SMC5 CFL1 CD93 CFHR2 TFRC TALDO1 TMSB4X ADA2 ARHGDIB H1-5 SERPING1 PZP TPI1 RYR2 MMP2 H1-2 LDHB SERPINA10 S100A8 CAP1 CDH13 HSP90B1 C8B LGALS3BP GAPDH S100A6 F9 TKT AGT H1-4 S100A9 |
